# A Systematic Survey of Reversibly Covalent Dipeptidyl Inhibitors of the SARS-CoV-2 Main Protease

**DOI:** 10.1021/acs.jmedchem.3c00221

**Published:** 2023-08-10

**Authors:** Zhi Zachary Geng, Sandeep Atla, Namir Shaabani, Veerabhadra Vulupala, Kai S. Yang, Yugendar R. Alugubelli, Kaustav Khatua, Peng-Hsun Chen, Jing Xiao, Lauren R. Blankenship, Xinyu R. Ma, Erol C. Vatansever, Chia-Chuan D. Cho, Yuying Ma, Robert Allen, Henry Ji, Shiqing Xu, Wenshe Ray Liu

**Affiliations:** † Department of Chemistry, Texas A&M Drug Discovery Laboratory, Texas A&M University, College Station, Texas 77843, United States; ‡ Sorrento Therapeutics, Inc. San Diego, California 92121, United States; § Department of Pharmaceutical Sciences, Irma Lerma Rangel College of Pharmacy, Texas A&M University, College Station, Texas 77843, United States; ∥ Department of Biochemistry and Biophysics, Texas A&M University, College Station, Texas 77843, United States; ⊥ Institute of Biosciences and Technology and Department of Translational Medical Sciences, College of Medicine, Texas A&M University, Houston, Texas 77030, United States; # Department of Molecular and Cellular Medicine, College of Medicine, Texas A&M University, College Station, Texas 77843, United States

## Abstract

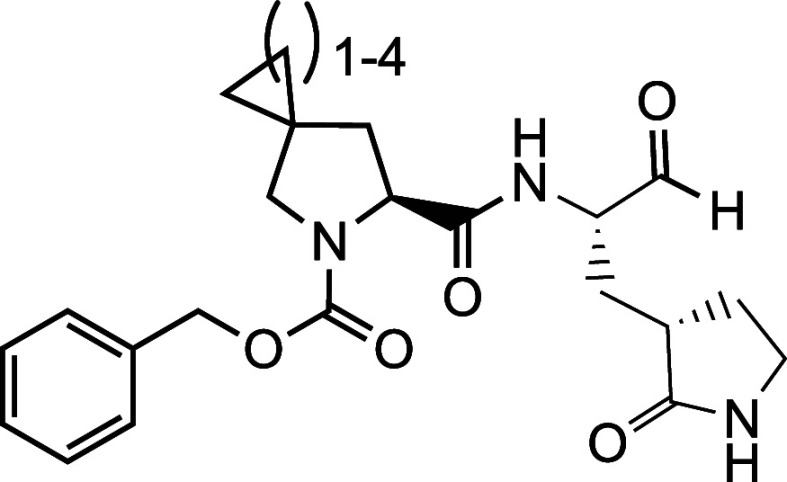

SARS-CoV-2, the COVID-19 pathogen, relies on its main protease (M^Pro^) for replication and pathogenesis. M^Pro^ is a demonstrated target for the development of antivirals for SARS-CoV-2. Past studies have systematically explored tripeptidyl inhibitors such as nirmatrelvir as M^Pro^ inhibitors. However, dipeptidyl inhibitors especially those with a spiro residue at their P2 position have not been systematically investigated. In this work, we synthesized about 30 dipeptidyl M^Pro^ inhibitors and characterized them on enzymatic inhibition potency, structures of their complexes with M^Pro^, cellular M^Pro^ inhibition potency, antiviral potency, cytotoxicity, and *in vitro* metabolic stability. Our results indicated that M^Pro^ has a flexible S2 pocket to accommodate inhibitors with a large P2 residue and revealed that dipeptidyl inhibitors with a large P2 spiro residue such as (*S*)-2-azaspiro [4,4]nonane-3-carboxylate and (*S*)-2-azaspiro[4,5]decane-3-carboxylate have favorable characteristics. One compound, MPI60, containing a P2 (*S*)-2-azaspiro[4,4]nonane-3-carboxylate displayed high antiviral potency, low cellular cytotoxicity, and high *in vitro* metabolic stability.

## Introduction

Coronaviruses (CoVs) are RNA pathogens that infect vertebrates including humans. Although mildly pathogenic CoVs were discovered in the 1960s, the first pandemic CoV, severe acute respiratory syndrome (SARS)-CoV, was yet to emerge until 2002.^1,2^ Since then, within 20 years, two more pandemic CoVs, Middle East respiratory syndrome (MERS)-CoV and SARS-CoV-2,^3−5^ appeared, with the latter wreaking havoc across the globe. All three pandemic CoVs were believed to have originated from animals and spread to humans during close human–animal interactions. The high outbreak frequency of CoV pandemics in the past two decades and the ever-increasing close human–animal interactions in modern society combinedly portend the future pandemic CoV outbreaks. With COVID-19 remaining and future CoV pandemics looming, it is paramount to develop orally available small-molecule drugs that can be easily distributed as CoV antivirals for both treatment and prevention. So far, three orally available medications including remdesivir, molnupiravir, and PAXLOVID have been approved for the treatment of COVID-19 patients.^6−8^ Both remdesivir and molnupiravir are nucleotide analogues. Remdesivir is an RNA replication inhibitor and known to have low efficacy in inhibiting SARS-CoV-2.^6^ On the contrary, molnupiravir is an RNA mutagen. Clinical tests showed that molnupiravir reduced the risk of hospitalization and death by 50% compared to placebo for patients with mild and moderate COVID-19.^9^ However, its mutagen nature that drives SARS-CoV-2 to undergo mutagenesis warrants use with caution. Unlike remdesivir and molnupiravir, PAXLOVID is a combination therapy of nirmatrelvir and ritonavir. Nirmatrelvir is a reversible covalent inhibitor of the SARS-CoV-2 main protease (M^Pro^) that serves an essential role in the viral pathogenesis and replication.^8^ Ritonavir is a human cytochrome P450 3A4 inhibitor that improves the metabolic stability of nirmatrelvir.^10^ The potential toxicity of PAXLOVID requires its stop of use after 5 days, and it has failed as a preventative for COVID-19 in clinical tests. The current published results have shown that nirmatrelvir is a substrate of P-glycoprotein multidrug transporter (P-pg) that continuously pumps various and structurally unrelated compounds to the outside of human cells.^8^ P-gp is known to have varied expression levels in different tissues. Although ritonavir is a P-gp inhibitor as well, the expression variation of P-gp in different tissues likely causes different inhibition efficacies of PAXLOVID.^11^ This may explain why many patients had COVID-19 rebound after stopping taking PAXLOVID and SARS-CoV-2 from these patients after COVID rebound did not show resistance to PAXLOVID. Because of concerns related to existing small-molecule SARS-CoV-2 antivirals, the research of developing other alternative SARS-CoV-2 antivirals is still needed.

**Figure 1:**
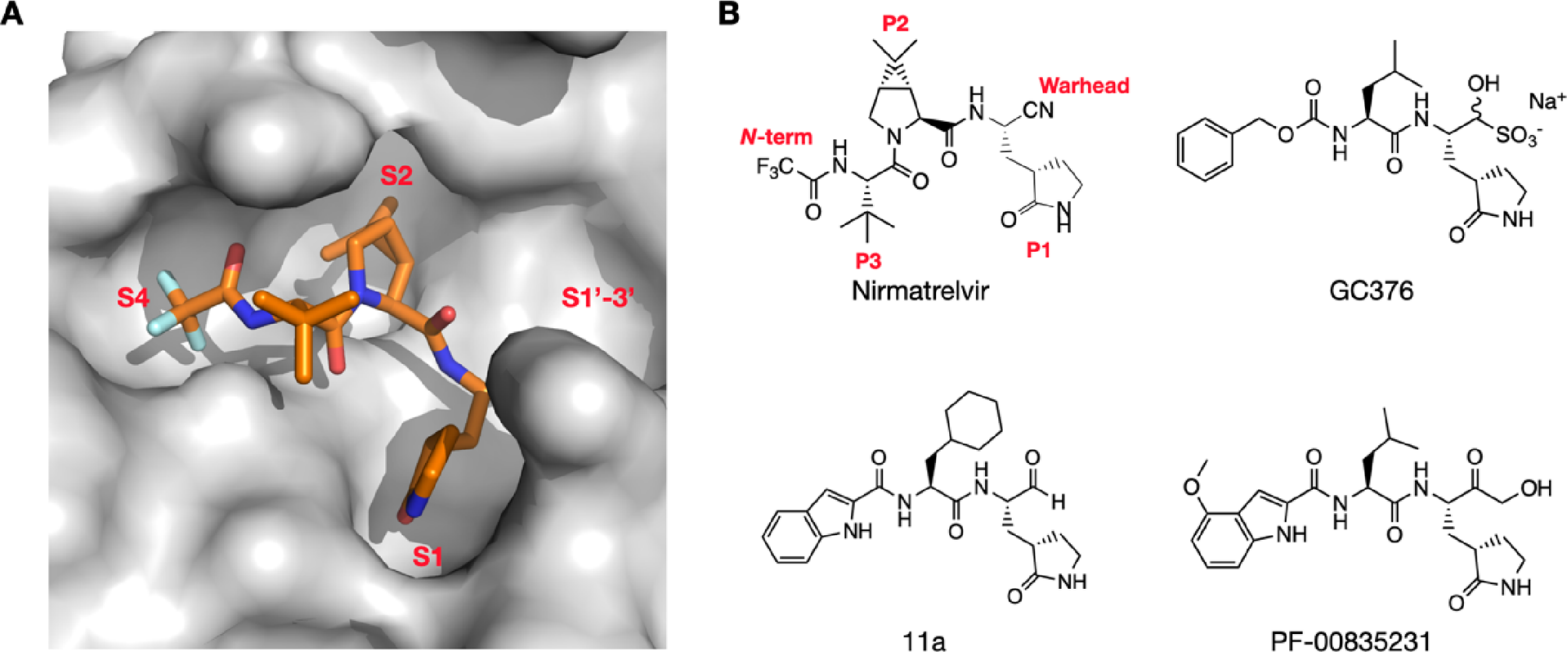
(A) The M^Pro^-nirmatrelvir complex. The structure is based on the PDB entry 7TE0.^12^ The contoured surface of M^Pro^ is shown. Four substrate binding pockets in M^Pro^ are labeled. (B) The structures of nirmatrelvir, GC376, 11a, and PF-00835231. Chemical positions in nirmatrelvir are labeled.

M^Pro^ is a cysteine protease that uses four binding pockets, S1, S2, S4, and S1′–3′, in the active site to engage P1, P2, P4 and P1′–3′ residues in a protein substrate for binding (Figure 1A).^13^ Nirmatrelvir can be classified as a tripeptidyl inhibitor that uses its P1 and P2 residues and *N*-terminal trifluoroacetyl group to bind S1, S2, and S4 pockets, respectively, in M^Pro^ and an activated nitrile warhead to covalently engage C145, the catalytic cysteine of M^Pro^ (Figure 1B).^8^ Similar to that in a protein substrate, the P3 side chain of nirmatrelvir does not directly interact with M^Pro^. Because P3 is not necessary for an inhibitor to engage M^Pro^ for binding, multiple potent dipeptidyl inhibitors that use their P1 and P2 residues and *N*-terminal group to bind S1, S2, and S4 pockets in M^Pro^ and a covalent warhead to engage C145 of M^Pro^ have also been reported. Representative dipeptidyl M^Pro^ inhibitors include GC376, 11a, and PF-00835231 (Figure 1B).^14−19^ However, a systematic study of dipeptidyl M^Pro^ inhibitors on how different chemical identities in P1 and P2 residues, *N*-terminal groups, and warheads influence M^Pro^ inhibition, structural aspects in binding M^Pro^, cellular and antiviral potency, and metabolic stability has not been reported. In this work, we wish to report a systematic survey of dipeptidyl M^Pro^ inhibitors of M^Pro^ and their potential use as SARS-CoV-2 antivirals.

## Results and Discussion

### The Design and Synthesis of Dipeptidyl M^Pro^ Inhibitors

**Figure 2:**
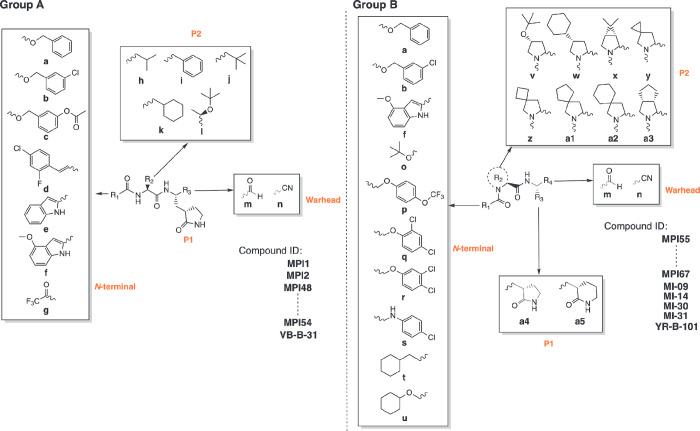
A diagram showing all dipeptidyl compounds that have been synthesized.

We followed two general designs shown in Figure 2 for the design and synthesis of dipeptidyl M^Pro^ inhibitors. Group A compounds were developed during the early phase of the pandemic.^20^ They were primary amino acid-based and contained a 3-methylpyrrolidin-2-one side chain at the P1 site due to the demonstrated high affinity of this side chain toward the S1 pocket of M^Pro^. Isopropyl (**h**), benzyl (**i**), *t*-butyl (**j**), and cyclohexylmethyl (**k**) as a side chain at the P2 site were previously tested in tripeptidyl M^Pro^ inhibitors and were included in the Group A compounds.^21^ Both P1 and P2 residues are in the L configuration. Our previous works showed that *O-t-*butyl-l-threonine (**l**) as the P3 residue in tripeptidyl M^Pro^ inhibitors led to high cellular and antiviral potency.^21,22^ We included this residue at the P2 site as well hoping to observe a similar effect. The *N*-terminal groups were chosen among carboxybenzyl (CBZ, **a**), 3-chloro-CBZ (**b**), 3-acetoxy-CBZ (**c**), 4-chloro-2-fluorocinnamoyl (**d**), 1*H*-indole-2-carbonyl (**e**), 4-methoxyl-1*H*-indole-2-carbonyl (**f**), and trifluoroacetyl (**g**). Some of these groups were used in inhibitors for either SARS-CoV or SARS-CoV-2 inhibitors.^15,23−32^ The warhead was chosen between aldehyde (**m**) and nitrile (**n**) that reversibly react with C145 of M^Pro^ to form hemithioacetal and thioimidate, respectively. Group B compounds were developed later, and all contained a modified proline at the P2 site. For this group of dipeptidyl M^Pro^ inhibitors, the P1 side chain was primarily 3-methylpyrrolidin-2-one (**a4**), and one inhibitor had a 3-methylpiperidin-2-one (**a5**) side chain due to the demonstrated high potency of some inhibitors with this side chain.^27^ Proline-based P2 residues in Group B compounds included (*R*)-3-*t*-butyloxyl-l-proline (**v**), (*R*)-3-cyclohexyl-l-proline (**w**), (1*S*,2*S*,5*R*)-6,6-dimethyl-3-azabicyclo[3,1,0]hexane-2-carboxylate (**x**) that is the P2 residue in nirmatrelvir, (*S*)-5-azaspiro[2,4]heptane-6-carboxylate (**y**), (*S*)-6-azaspiro[3,4]octane-7-carboxylate (**z**), (*S*)-2-azaspiro[4,4]nonane-3-carboxylate (**a1**), (*S*)-2-azaspiro[4,5]decane-3-carboxylate (**a2**), and (1*S*,2*S*,5*R*)-3-azabicyclo[3,3,0]octane-2-carboxylate (**a3**). A survey of multiple M^Pro^-inhibitor complex structures revealed that the peptide region aa46–51 in M^Pro^ that caps the S2 pocket is highly flexible, and this structural flexibility allows even the flipping of C44 close to 180 °C to form a Y-shaped, S-O-N-O-S-bridged cross-link with two other residues, C22 and K61, in M^Pro^.^33,34^ This structural flexibility and the cross-link formation leave a much more open, large S2 pocket that accommodates potentially a large P2 residue in a peptidyl inhibitor. Proline-based P2 residues in Group B compounds were designed for this reason to test how deep and bulky the S2 pocket can turn to be. **V** and **w** are 3-substituted prolines, **x** and **a4** are bicyclic compounds, and **y**–**a2** are spiro compounds. They were selected for the synthetic accessibility. The *N*-terminal groups for Group B compounds were more diverse than those in Group A. Besides several moieties used in Group A, other *N*-terminal groups included *t*-butyloxycarbonyl (Boc, **o**), 4-trifluoromethoxyphenoxycarbonyl (**p**), 2,4-dichlorophenoxycarbonyl (**q**), 3,4-dichlorophenoxycarbonyl (**r**), 4-chlorophenylcarbamoyl (**s**), 3-cyclohexylpropanoyl (**f**), and 2-cyclohexyloxyacetyl (**u**). Some of these *N*-terminal groups were previously used in dipeptidyl M^Pro^ inhibitors.^15,23−32^ Others were designed to explore different interactions with the S4 pocket of M^Pro^. The warhead was chosen between aldehyde (**m**) and nitrile (**n**) as well.

**Scheme 1:**
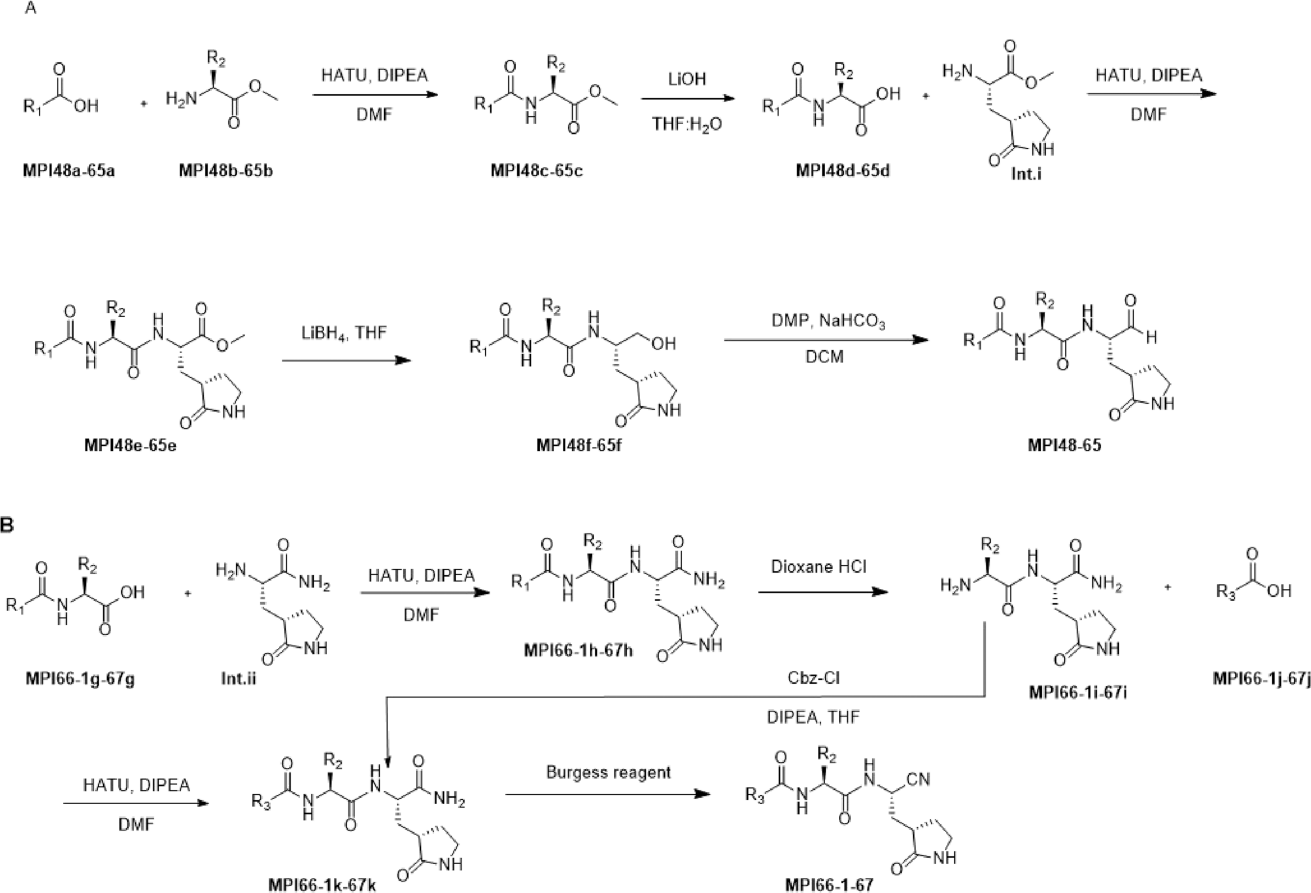
Synthetic Routes

We followed two synthetic routes shown in Scheme 1 for the synthesis of aldehyde and nitrile-based dipeptidyl M^Pro^ inhibitors. In total, 29 dipeptidyl M^Pro^ inhibitors were synthesized including MI-09, MI-14, MI-30, and MI-31, four compounds that were previously developed by a different lab and included as comparison.^28^ MPI1, MPI2, and GC376 are three dipeptidyl M^Pro^ inhibitors that were previously characterized. They are included for comparison as well. All inhibitors have their compositions shown in Table 1, and their chemical structures are presented in Figure S1 as well.

**Table 1:**
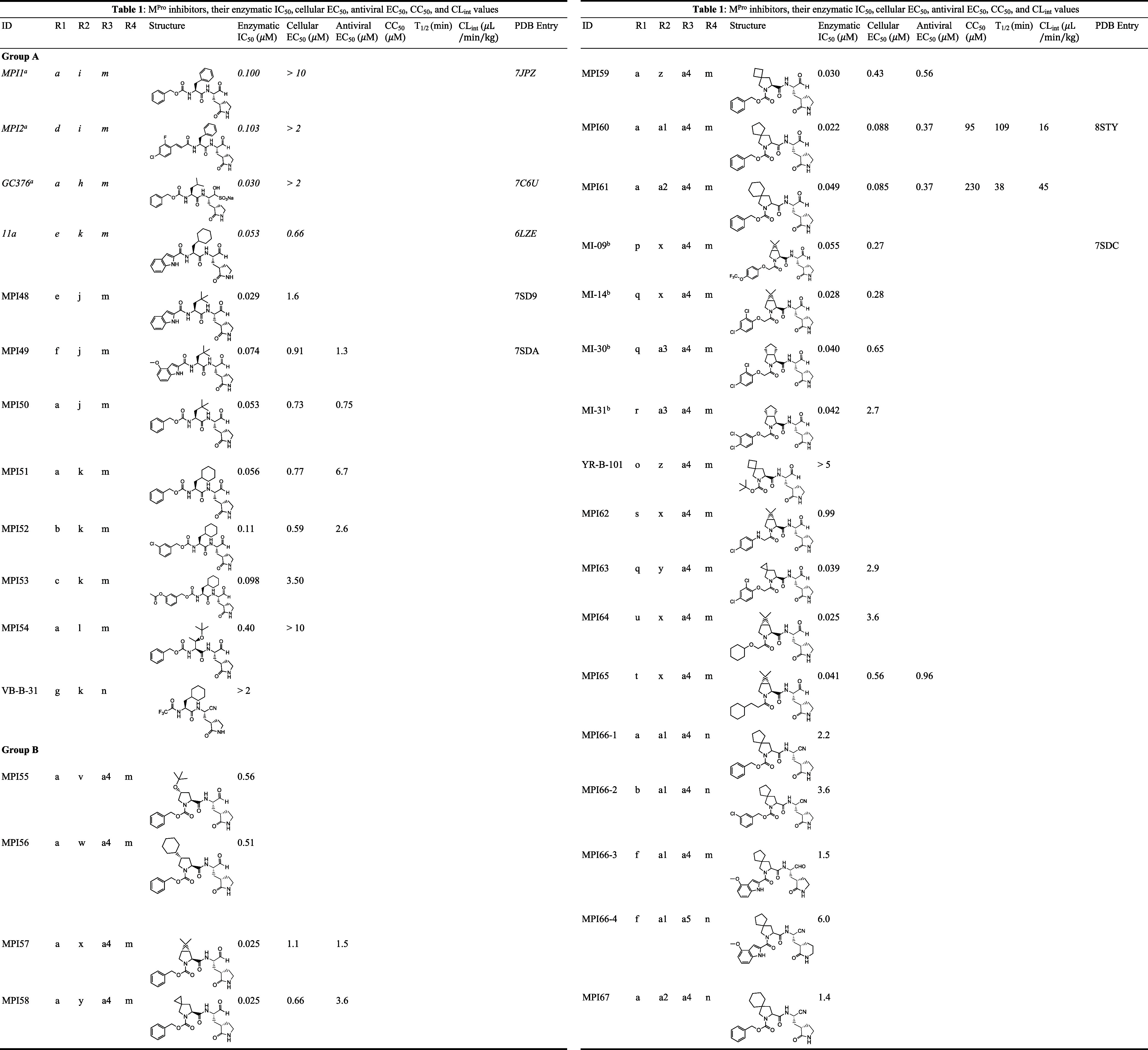
M^Pro^ Inhibitors and Their Enzymatic IC_50_, Cellular EC_50_, Antiviral EC_50_, CC_50_, and CL_int_ Values

a Data were taken from Cao *et al.*(35)

b Recharacterized compounds from Qiao *et al.*(28)

### The Enzymatic Inhibition Potency of Dipeptidyl M^Pro^ Inhibitors

**Figure 3:**
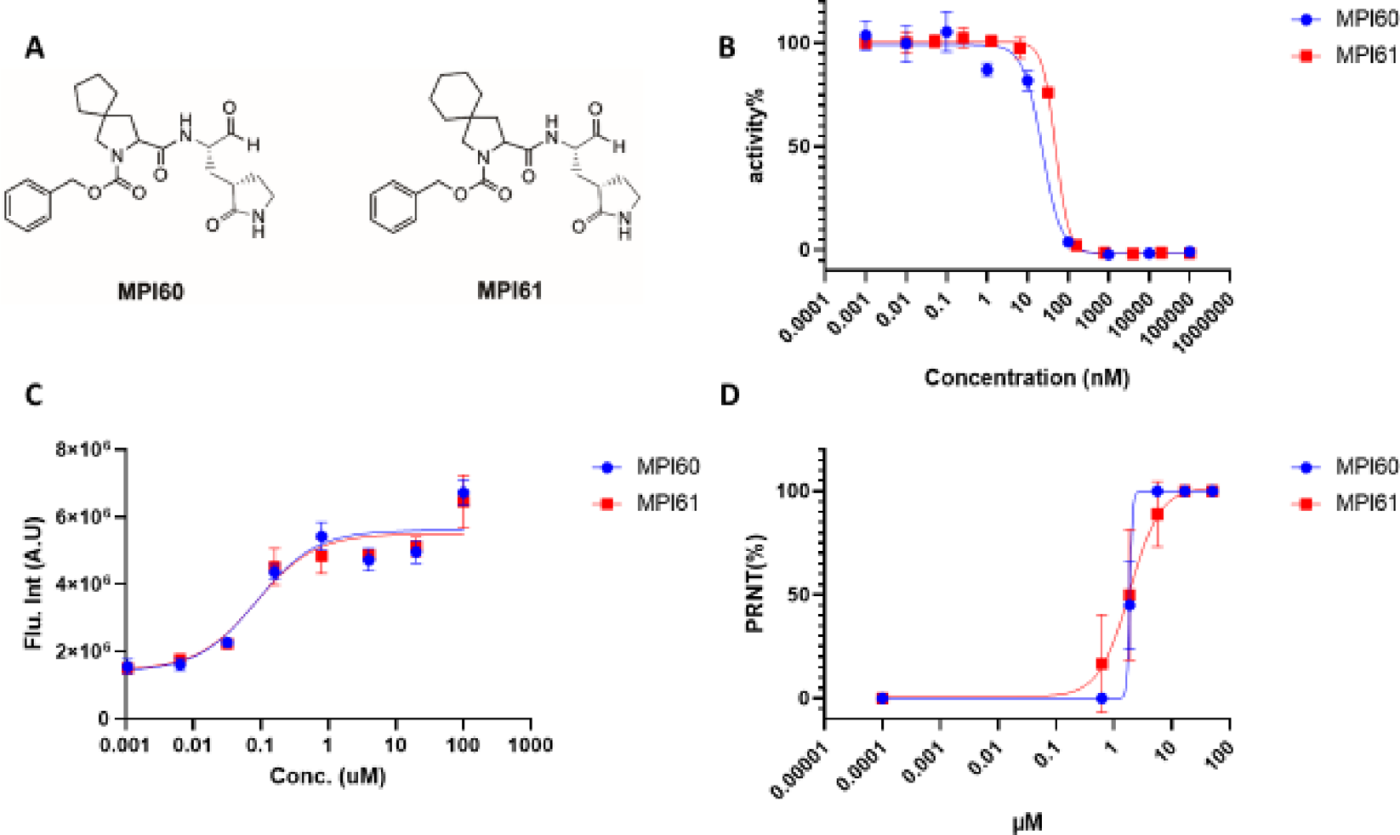
(A) Structures of MPI60 and MPI61. (B) *In vitro* M^Pro^ inhibition curves of MPI60 and MPI61. (C) Cellular M^Pro^ inhibition curves of MPI60 and MPI61 in 293T cells transiently expressing M^Pro^-eGFP. (D) SARS-CoV-2 (USA-WA1/2020) inhibition curves of MPI60 and MPI61 in Vero E6 cells.

We followed a previously established protocol that uses Sub3 (Dabcyl-KTSAVLQSGFRKME-Edans), a fluorogenic peptide substrate of M^Pro^, to determine IC_50_ values for all synthesized compounds.^36^ In this assay, we incubated M^Pro^ with a compound for 30 min before Sub3 was added, and the fluorescent product formation (Ex: 336 nm/Em: 455 nm) was recorded and analyzed to determine IC_50_. The 30 min incubation time is a standard procedure that has been used by multiple labs in the determination of IC_50_ values for M^Pro^ inhibitors.^19,37,38^ Because all synthesized compounds are reversible covalent inhibitors, their incubation times with M^Pro^ are not expected to significantly influence their determined IC_50_ values. A previous test of a reversible covalent inhibitor in three different incubation times, 15, 30, and 60 min, led to very similar determined IC_50_ values.^21^ Determined IC_50_ values for all compounds are presented in Table 1. The M^Pro^ inhibition curves for MPI60 and MPI61 are shown in Figure 3B, and those for other compounds are shown in Figure S2. For Group A compounds, except MPI54 and VB-B-31, all others have an IC_50_ value around or below 100 nM, comparable to that for MPI1, MPI2, and GC376. MPI54 has *O-t-*butyl-threonine at the P2 site. The S2 pocket of M^Pro^ is known to prefer leucine, phenylalanine, and their analogues at the P2 site of substrates and inhibitors. It was not surprising that the installation of *O-t-*butyl-threonine at the P2 site in MPI54 led to weaker binding. However, it was intriguing to observe that *O-t-*butyl-threonine at the P2 site in MPI54 that is structurally very different from leucine and phenylalanine did not significantly distort the binding to M^Pro^ compared to MPI1. This observation indicates that the high structural plasticity of the S2 pocket can potentially accommodate a large variety of structurally unique and bulky P2 residues and inspired us to design compounds in Group B.^33^ VB-B-31 has a small *N*-terminal group and nitrile warhead. Because the nitrile warhead is demonstrated to engage the catalytic cysteine very efficiently, the low enzymatic inhibition potency of VB-B-31 is likely due to the relatively small trifluoroacetyl *N*-terminal group not being able to engage the S4 pocket. MPI1, GC376, MPI50, and MPI51 are structurally different only at the P2 site with a leucine, phenylalanine, or analogue. They have similar IC_50_ values, indicating that the M^Pro^ S2 pocket has similar binding preference toward leucine, phenylalanine, and their derivatives with a similar size. MPI48, MPI49, and MPI50 differ at the *N*-terminal group and have similar IC_50_ values. Compared to CBZ (**a**), 1*H*-indole-2-carbonyl (**e**) and 4-methoxyl-1*H*-indole-2-carbonyl (**f**) are more structurally rigid. Previous works have already shown that these three groups involve different interactions with M^Pro^.^14,15^ Their contributions to similar binding toward M^Pro^ are likely accidental. MPI51, MPI52, and MPI53 are structurally similar compounds. MPI52 and MPI53 have a 3-substituted benzene on the *N*-terminal CBZ group. Both 3-chloro and 3-acetoxy groups in MPI52 and MPI53, respectively, did not significantly influence the binding to M^Pro^. Previously determined structures of M^Pro^ complexed with dipeptidyl inhibitors showed a loosely bound *N*-terminal CBZ.^20^ This relatively weak engagement of CBZ to M^Pro^ may explain the relatively weak influence of a substitution in CBZ toward binding to M^Pro^. Although this information is not useful in the design of more potent inhibitors, it is helpful in the design of metabolically stable compounds because adding substitutions to CBZ can significantly change its metabolic stability.

Group B compounds were designed and synthesized based on information learned from Group A compounds and other developments by exploring bicyclic side chains in the P2 residue of M^Pro^ inhibitors such as nirmatrelvir, MI-09, MI-14, and MPI29-MPI47.^8,22,28^ MPI55 and MPI56 are two inhibitors with a large 3-substitution at its P2 proline. The P2 (*R*)-4-cyclohexyl-L-proline (**w**) in MPI56 is also a highly rigid residue. Although both inhibitors showed relatively mild inhibition potency compared to other inhibitors, with IC_50_ values a little higher than that for MPI54, their IC_50_ values of around 0.5 μM still indicate that they can engage M^Pro^ for binding efficiently. (*R*)-4-Cyclohexyl-l-proline (**w**) is probably the largest P2 residue that has been tested so far. It is obvious that the S2 pocket of M^Pro^ can rearrange to accommodate large and bulky P2 residues in inhibitors, corroborating the discovery made previously in multiple M^Pro^ crystal structures.^33,34^ Therefore, it is possible that other large P2 residues with strong M^Pro^ binding can be developed. This potential needs further exploration. MPI57–MPI61 are structurally similar compounds with variation at the P2 site. They all have an *N*-terminal CBZ (**a**), P1 3-methylpyrrolidin-2-one side chain, and aldehyde warhead. Their P2 side chain varied among bicyclic and spiro moieties **x**–**a2**. All five compounds displayed very high M^Pro^ inhibition potency with an IC_50_ value below 50 nM. Four compounds, MPI57–MPI60, have an IC_50_ value at or below 30 nM. And MPI60 has the lowest IC_50_ value at 22 nM among all compounds that were tested in this series and is one of the most potent M^Pro^ inhibitors that have been developed so far. From the structural perspective, **w**–**a2** can be considered as leucine and phenylalanine analogues with higher structural rigidity by forming a proline ring. This high structural rigidity likely contributes to the strong binding of these moieties to the M^Pro^ pocket. From MPI57 to MPI61, there is a significant increase of the size of P2 side chain. The surprisingly similar IC_50_ values determined for all five compounds prove the high structural plasticity of the M^Pro^ S2 pocket.^33^ MI-09, MI-14, MI-30, and MI-31 are four previously reported dipeptidyl M^Pro^ inhibitors.^28^ They were synthesized and recharacterized for comparison with all other dipeptidyl M^Pro^ inhibitors. All four molecules have a determined IC_50_ value around or below 50 nM. MI-09 and MI-14 have a P2 **x** residue but two different *N*-terminal groups, **p** and **q**, respectively. These two compounds are structurally similar to MPI57 but with the *N*-terminal carbamate oxygen in MPI57 moved one position and additional substituent(s) added to the *N*-terminal phenyl group. Both MI-09 and MI-14 have a similar and slightly higher IC_50_ value than MPI57. This is likely due to the relatively loosely bound *N*-terminal CBZ like group to M^Pro^. MI-30 and MI-31 have a P2 **a3** residue and **q** and **r**, respectively, as their *N*-terminal groups. Both have an IC_50_ value similar to other compounds that have a bicyclic or spiro residue at the P2 site. Given that the size of **a3** is similar to **x**, this is expected. A compound, YR-B-101, that is structurally similar to MPI59 but with an *N*-terminal BOC (**o**) group was also made. This compound showed very weak enzymatic inhibition potency. Because compound 11a has an *N*-terminal indole that uses its indole imine to form a hydrogen bond with the backbone carbonyl oxygen of E66 in M^Pro^ that potentially contributes to the strong binding of 11a to M^Pro^, we tried to recapitulate this interaction by introducing **s** as an *N*-terminal group for MPI62 that is structurally similar to MPI57, MI-09, and MI-14. Unfortunately, the determined IC_50_ value is drastically higher than those for the other three compounds. Two possibilities may contribute to the affinity decrease. The introduction of a hydrogen bond donor likely makes the molecule more favorable to be dissolved in water. The proposed hydrogen bond may not form as well. Because MI-14 that has an *N*-terminal **q** group has a lower IC_50_ value than MI-09, we grafted this moiety into MPI58 to afford MPI63. Compared to MPI58, MPI63 has a higher determined IC_50_ value. On the basis of all collected data so far, we could conclude that the CBZ (**a**) group is the best *N*-terminal group for dipeptidyl M^Pro^ inhibitors to achieve high potency. Because the *N*-terminal CBZ (**a**) group only loosely binds to M^Pro^, we thought that changing it to **t** and **u** that have a saturated cyclohexane might introduce better interactions with M^Pro^. Replacing the CBZ (**a**) group in MPI57 with **u** and **t** afforded MPI64 and MPI65, respectively. MPI64 has a determined IC_50_ value the same as MPI57. MPI65 has a slightly higher IC_50_ value. Although using **t** and **u** did not lead to more potent inhibitors, the results demonstrated that *N*-terminal groups other than the CBZ (**a**) group can lead to equal enzymatic inhibition potency. On the basis of all discussed compounds, optimal P2 residues are two primary amino acids, **h** and **j**, and all tested bicyclic and spiro amino acids, **x**–**a3**. Because they led to very similar enzymatic inhibition potency, it is difficult to conclude which one is the best, although MPI60 that has a P2 **a1** residue has the lowest IC_50_ value among all tested compounds.

Previous works with tripeptidyl and dipeptidyl M^Pro^ inhibitors showed that replacing the aldehyde (**m**) warhead with nitrile (**n**) can still lead to potent inhibitors.^8^ To recapitulate this observation, we synthesized MPI66-1 that contained **n** and was structurally different from MPI60 only at the warhead and MPI66-2 that had an additional 3-chloro substituent on the *N*-terminal group. However, both compounds showed much lower potency than MPI60 and had an IC_50_ value above 2 μM. It is possible that **a2** at the P2 site introduces unique M^Pro^-inhibitor interactions that make the covalent interaction between M^Pro^ C145 and nitrile (**n**) less favorable than that in the M^Pro^-nirmatrelvir complex.^12^ Replacing the *N*-terminal CBZ (**a**) group in MPI60 with **f** to afford MPI66-3 to recapitulate strong binding that was observed in the M^Pro^-11a complex also failed. MPI66-3 had a determined IC_50_ value above 1 μM, drastically higher than that for 11a.^15^ This observation corroborated the possible unique interactions with M^Pro^ induced by **a2** at the P2 site. A previous work showed that a 3-methylpiperidin-2-one (**a5**) side chain led to better enzymatic inhibition potency in a dipeptidyl inhibitor than its corresponding 3-methylpyrrolidin-2-one (**a4**) inhibitor.^27^ To rescue the potency of MPI66-1, we replaced its P1 **a4** residue with **a5** to afford MPI66-4. However, MPI66-4 exhibited even lower inhibition potency with a determined IC_50_ value of 6.0 μM. As discussed below, MPI61 that contains **a2** at its P2 site showed high cellular and antiviral potency. On the basis of this information, we synthesized MPI67, a nitrile (**b**)-containing MPI61 equivalent. However, this molecule showed low enzymatic inhibition potency with a determined IC_50_ value of 1.4 μM. This is similar to other nitrile-based dipeptidyl inhibitors that were developed in our series. As discussed above, we suspect that the spiro residue at the P2 position might involve unique interactions with M^Pro^ that disfavor the covalent interaction between the nitrile warhead and the M^Pro^ C145. In our series of dipeptidyl M^Pro^ inhibitors, we concluded that the aldehyde (**m**) warhead is better than the nitrile (**n**) warhead in achieving high enzymatic inhibition potency in dipeptidyl M^Pro^ inhibitors.

### Cellular M^Pro^ Inhibition and Antiviral Potency of Dipeptidyl Inhibitors

M^Pro^ showed acute toxicity to human cells when it was recombinantly expressed. On the basis of this observation, we developed a cell-based assay to characterize cellular potency of M^Pro^ inhibitors.^35^ In this assay, an inhibitor with cellular potency inhibits cytotoxicity from an M^Pro^-eGFP (enhanced green fluorescent protein) fusion protein that is transiently expressed in 293T cells and consequently leads to host cell survival and enhanced overall expression of M^Pro^-eGFP that can be quantified by flow cytometry. This assay allows the quick assessment of M^Pro^ inhibitors in cells by avoiding tedious characterizations of cellular permeability and stability of developed compounds. This assay is also more accurate in assessing M^Pro^ inhibition in cells than a direct antiviral assay because a compound may block functions of host proteases such as TMPRSS2, furin, and cathepsin L that are critical for SARS-CoV-2 infection and therefore provide false-positive results about M^Pro^ inhibition in cells.^39−41^ Using this cellular assay system, we previously characterized a number of repurposed SARS-CoV-2 inhibitors and pointed out that some repurposed inhibitors inhibit SARS-CoV-2 via mechanisms different from M^Pro^ inhibition.^35^ Compounds that showed potency in this cellular assay displayed roughly similar potency in antiviral tests. Using this cellular assay, we characterized all synthesized inhibitors in this work that displayed an enzymatic inhibition IC_50_ value below 0.5 μM. The characterized cellular M^Pro^ inhibition EC_50_ values are presented in Table 1. Inhibition curves for MPI60 and MPI61 are presented in Figure 3C, and the rest are shown in Figure S3. For Group A compounds with *t*-butylalanine (**j**) and cyclohexylalanine (**k**) at the P2 site, they showed measurable cellular EC_50_ values. Four compounds, MPI49–MPI52, had determined cellular EC_50_ values below 1 μM. In comparison to other peptidyl inhibitors with leucine (**h**) and phenylalanine (**i**) at the P2 site, these compounds showed generally better cellular M^Pro^ inhibition potency, indicating that both **j** and **k** are favorable residues at the P2 site for improved cellular M^Pro^ inhibition potency. **j** and **k** at the P2 site likely improve the cellular permeability or stability of their containing compounds in cells. MPI54 that has a P2 *O*-*t*-butylthreonine (**l**) showed very weak cellular potency. In a previous work, we showed that a P3 *O*-*t*-butylthreonine (**l**) generally improves the cellular potency of tripeptidyl M^Pro^ inhibitors. The low cellular potency of MPI52 indicated that a similar effect cannot be achieved by moving *O*-*t*-butylthreonine (**l**) from P3 to the P2 site.

All Group B compounds with an IC_50_ value below 0.5 μM had measurable cellular M^pro^ inhibition potency. The two most potent compounds are MPI60 and MPI61 that showed determined cellular EC_50_ values below 100 nM. One interesting observation was that among MPI57–MPI61, all spiro compounds performed better than MPI57 that has a P2 bicyclic residue as in nirmatrelvir. Among all spiro compounds MPI58–MPI61, cellular potency was positively correlated with the size of the P2 spiro structure. The large size of the P2 residue likely improves the cellular permeability of their corresponding compounds. For the four previously reported compounds, MI-09, MI-14, MI-30, and MI-31, they displayed mild (>0.5 μM) to high (<0.5 μM) cellular potency, but none of their potency reached the level of MPI60 and MPI61. Compared to MPI57, **q** as the *N*-terminal group in MI-14 led to better cellular potency. However, replacing the *N*-terminal group in MPI58 with **q** to afford MPI63 led to worse cellular potency, indicating that the *N*-terminal group effects in cellular potency cannot be generalized. Replacing the *N*-terminal CBZ (**a**) group of MPI57 with **u** in MPI64 and **t** in MPI65 had opposite effects with worse cellular potency for MPI64 and better cellular potency for MPI65. However, the cellular potency of MPI65 was still significantly lower than those of MPI60 and MPI61. Among all inhibitors in both Group A and Group B, MPI60 and MPI61 had the highest cellular potency. Peptidyl M^Pro^ inhibitors with a P2 residue that has either an aliphatic or bicycle side chain have been extensively explored. However, peptidyl M^Pro^ inhibitors with a spiro P2 residue have not been studied much. As the first of its kind, the current work revealed that peptidyl M^Pro^ inhibitors with a spiro **a1** or **a2** residue at the P2 position significantly outperform other inhibitors.

For newly developed inhibitors that showed cellular potency with an EC_50_ value below 1 μM, we went further to characterize their antiviral potency. MPI57 was included for comparison with MPI58–MPI61. For four previously developed compounds, MI-09, MI-14, MI-30, and MI-31, their antiviral tests were not conducted because they were characterized in a previously reported paper^28^ and their detected cellular potency was lower than those of MPI60 and MPI61. To quantify antiviral EC_50_, we conducted plaque reduction neutralization tests of SARS-CoV-2 (USA-WA1/2020) in Vero E6 cells for all four inhibitors. We infected Vero E6 cells by virus in the presence of an inhibitor at various concentrations for 3 days and then quantified viral plaque reduction. On the basis of viral plaque reduction data, we determined antiviral EC_50_ values for all tested inhibitors. The determined antiviral EC_50_ values are presented in Table 1. The antiviral curves for MPI60 and MPI61 are shown in Figure 3D, and the rest are shown in Figure S4. They all had measurable antiviral EC_30_ values below 10 μM. Five compounds, MPI50, MPI59, MPI60, MPI61, and MPI65, had antiviral EC_50_ values below 1 μM. The same as shown in the cellular potency tests, MPI60 and MPI61 had the highest antiviral potency with the same EC_50_ value of 0.37 μM. So, we can conclude that a spiro **a1** or **a2** residue at the P2 position in a dipeptidyl M^Pro^ inhibitor leads to favorable antiviral potency and performs better than **x** that has been used in nirmatrelvir.

### Cytotoxicity and *In Vitro* Metabolic Stability of MPI60 and MPI61

Because of their high antiviral potency, MPI60 and MPI61 were advanced to characterizations of cytotoxicity and *in vitro* metabolic stability. For the cytotoxicity characterization, we used 293T cells and the MTT assay.^42^ Determined CC_50_ values for MPI60 and MPI61 were 95 and 230 μM, respectively (Table 1). The cytotoxicity curves for both compounds are presented in Figure S5 as well. Both MPI60 and MPI61 showed low toxicity. These CC_50_ values are similar to that of nirmatrelvir and lead to high calculated selectivity indices (CC_50_/antiviral EC_50_) for both compounds. The *in vitro* metabolic stability analysis for MPI60 and MPI61 was conducted using human liver microsomes. Their determined CL_int_ values were 16 (*t*_1/2_: 109 min) and 45 (*t*_1/2_: 38 min) μL/min/kg, respectively. The *in vitro* 109 min half-life makes MPI60 one of the most stable M^Pro^ inhibitors that have been developed so far. All collected data combined showed that MPI60 has the most favorable characteristics among all synthesized dipeptidyl inhibitors for advanced studies.

### X-ray Crystallography Analysis of M^Pro^ Bound with MPI48, MPI49, MI-09, and MPI60

**Figure 4:**
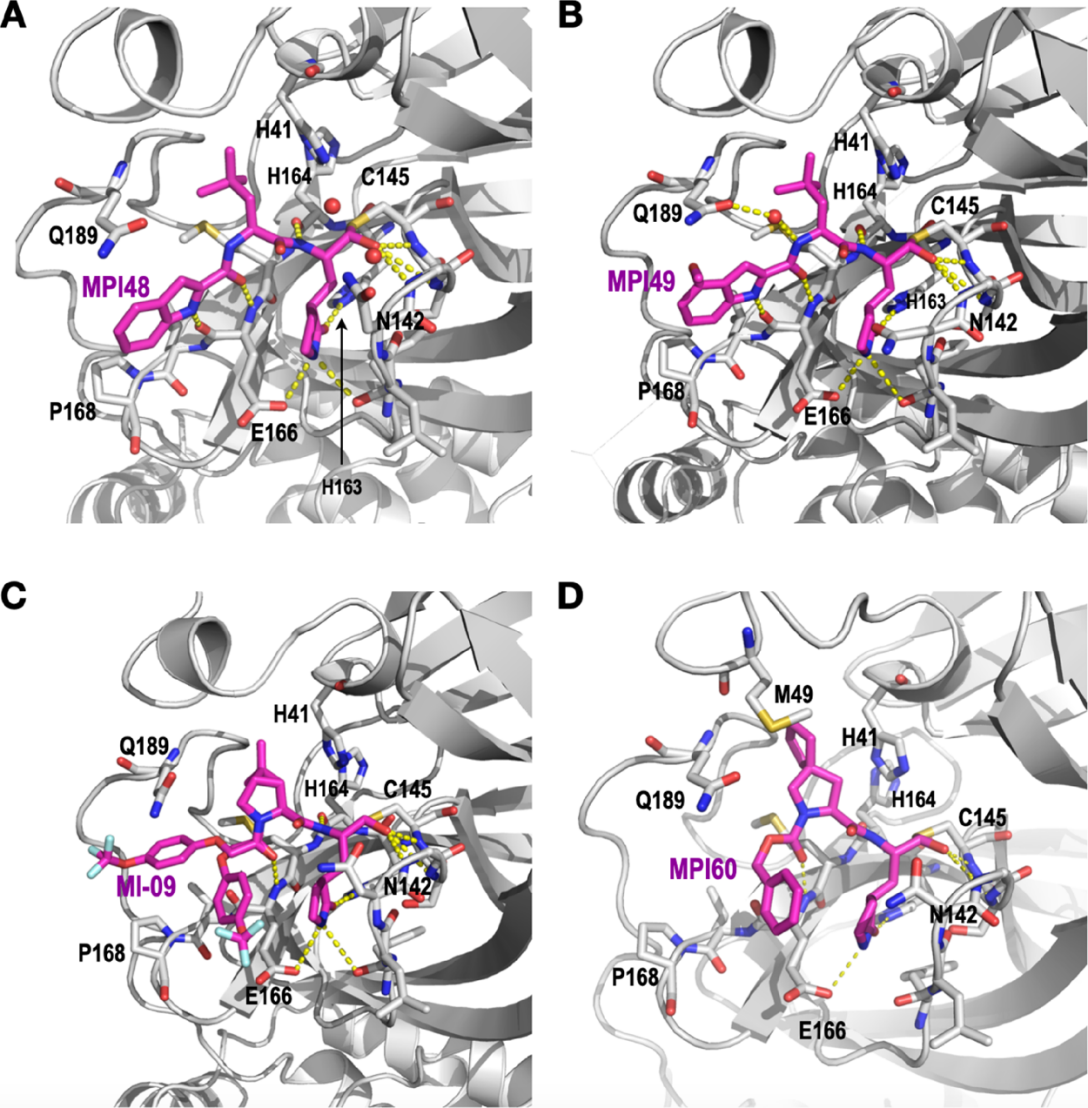
The crystal structures of (A) M^Pro^-MPI48 (PDB ID: 7SD9), (B) M^Pro^-MPI49 (PDB ID: 7SDA), (C) M^Pro^-MI-09 (PDB ID: 7SDC), and (D) M^Pro^-MPI60 (PDB ID: 8STY).

We previously determined the crystal structure of M^Pro^ bound with MPI1.^20^ In this structure (PDB: 7JPZ), the *N*-terminal CBZ (**a**) group of MPI1 did not show strong defined electron density around it. Because the *N*-terminal group of MPI1 is similar to that in most Group A compounds, we chose to conduct X-ray crystallography analysis of M^Pro^ bound with MPI48 and MPI49 in Group A because both have an *N*-terminal group not based on CBZ. We followed a previously established procedure to conduct the X-ray crystallography analysis of M^Pro^ bound with MPI48 and MPI49, respectively.^20^ We crystalized M^Pro^ in its apo form and then soaked obtained crystals with MPI48 or MPI49 before these crystals were mounted on an X-ray diffractometer for X-ray diffraction data collection. Collected data were then used to refine structures for M^Pro^ bound with MPI48 and MPI49, respectively. For M^Pro^-MPI48, the structure was determined with a resolution of 1.85 Å. As shown in Figure S6A, the electron density at the active site of M^Pro^-MPI48 clearly showed the bound inhibitor and allowed the unambiguous refinement of all nonhydrogen atoms. The three methyl groups at the P2 *t*-butyl group were clearly observable. There was continuous electron density that connected the thiolate of M^Pro^ C145 with the P1 Cα atom of MPI48, indicating a covalent bond formation. Interactions between MPI48 and M^Pro^ in the active sites are shown in Figure 4A. The electron density around the P1 Cα of MPI48 allowed the refinement of a hemithioacetal hydroxyl group that had an *S* confirmation and pointed exactly at the anion hole with a hydrogen bond distance to three backbone α-amines from M^Pro^ residues G143, S144, and C145. This strict *S* confirmation of hemithioacetal hydroxide has been observed in M^Pro^ bound with other aldehyde-based inhibitors.^20−22^ The P1 side chain lactam used its amide oxygen to form a hydrogen bond with the H163 imidazole nitrogen and its amide nitrogen to form two hydrogen bonds with the E166 side chain carboxylate and the F140 backbone α-amide oxygen. The P1 α-amine engaged the H164 backbone α-amide oxygen to form a hydrogen bond. The P2 *t*-butyl group fit neatly to the S2 pocket and was in close distance to side chains of H41, M165, and E189. M49 is a residue in the aa45–51 region that caps the S2 pocket. Its side chain was observed to fold into the S2 pocket in apo-M^Pro^ but typically flipped its position to open the S2 pocket to bind a peptidyl inhibitor. In determined M^Pro^-inhibitor complexes that were co-crystalized and had a closed active site due to protein packing in crystals, the M49 side chain was usually observed to cap the S2 pocket. However, our M^Pro^ crystals were obtained with an open active site allowing structural rearrangement around the active site, and soaking them with peptidyl inhibitors always led to a flexible aa45–51 region whose structure could not be refined. In M^Pro^-MPI48, there was also no strong electron density at this region to allow refining its structure, indicating a high structural flexibility of aa45–51. MPI48 has an *N*-terminal 1*H*-indole-2-carbonyl (**e**) group. It used its carbonyl oxygen and indole nitrogen to form a hydrogen bond with the E166 α-amine and α-carbonyl oxygen, respectively. The hydrogen bond between the MPI48 indole nitrogen and the E166 α-carbonyl oxygen is unique to MPI48 and other dipeptidyl inhibitors with an *N*-terminal 1*H*-indole-2-carbonyl (**e**) group or analogue. The same hydrogen bond has been observed in M^Pro^ bound with other similar dipeptidyl inhibitors such as 11a as well.^15^ The M^Pro^-MPI49 complex structure was refined to a resolution of 1.85 Å. As shown in Figure S6B, the active site electron density allowed the structural refinement of all chemical compositions of MPI49 except the *O*-methyl moiety of the *N*-terminal group. In the structure, there were no defined interactions with M^Pro^ that could make this *O*-methyl moiety adopt a fixed confirmation. As shown in Figure 4B, MPI49 involved interactions that are both covalent and other types with M^Pro^ that were mostly observed in the M^Pro^-MPI48 complex. One additional hydrogen bond that formed between the P2 α-amine and a water molecule was observed in M^Pro^-MPI49. This water molecule was also within a hydrogen bond distance to the Q189 side chain amide.

Most Group B compounds are structurally similar. We chose MI-09 and MPI60 as representatives for the structural characterization due to their high potency. MI-09 was a previously published compound that showed antiviral potency in mice.^28^ However, the structure of its complex with M^Pro^ was not reported. We used our established soaking method to determine and refine the structure of M^Pro^ bound with MI-09 to a resolution of 1.85 Å. As shown in Figure S6C, the electron density at the active site of M^Pro^-MI-09 showed a defined confirmation for the P1 and P2 residues and hemithioacetal hydroxide that was generated after the covalent interaction between MI-09 and C145 of M^Pro^. However, the MI-09 *N*-terminal groups adopted two confirmations that were clearly observable. The collected data allowed the refinement only for the *N*-terminal phenyl group but not its 4-trifluoromethoxy substituent. Except at the *N*-terminal group, MI-09 involved hydrogen bonds formed with M^Pro^ the same as MPI48 (Figure 4C). The P2 side chain of MI-09 has a rigid confirmation that fit nicely to the S2 pocket of M^Pro^. The P2 proline backbone in MI-09 pushed the side chain of Q189 to adopt a confirmation different from that in M^Pro^-MPI48. As observed in the other two M^Pro^-inhibitor structures, the aa45–51 region had an undefined confirmation. The structure of M^Pro^-MPI60 was refined to a resolution of 1.90 Å. The electron density at the active site as shown in Figure S6D allowed unambiguous fitting of MPI60 at its warhead that covalently interacted with C145 to generate a thiohemiacetal with an *S* configuration, the P1 residue, and the P2 residue. Although there was strong electron density that allowed the assignment of the *N*-terminal CBZ group capping on top of the P1 residue, there was also residual electron density within the M^Pro^ pocket as shown in Figure S6D indicating partial occupancy of the S4 pocket by the MPI60 *N*-terminal CBZ group. Inhibitors that adopted a similar conformation after binding to M^Pro^ have been reported.^14,19^ It is possible that the *N*-terminal CBZ group does not fit into the M^Pro^ S4 pocket well but involves strong van der Waals interactions with the P1 residue. Hydrogen bonding interactions with M^Pro^ that involve the MPI60 P1 and P2 residues as shown in Figure 4D are similar to those observed in all the other three M^Pro^-inhibitor complexes. However, unlike in the other three M^Pro^-inhibitor complexes, the aa45–51 region was partially observable. The side chain of M49 that was partially defined made van der Waals interactions with the P2 spiro side chain of MPI60. From the structure, it does look like that the MPI60 P2 spiro **a1** residue fits neatly to the M^Pro^ S2 pocket, explaining the high potency of MPI60.

In all four determined structures, the inhibitors did not fully engage the S4 pocket. MPI48 and MPI49 had an *N*-terminal group that was structurally defined in their M^Pro^ complexes due to its rigidity. But the *N*-terminal group in either molecule did not interact with the S4 pocket. MI-09 showed two conformations for its *N*-terminal group at the active site of M^Pro^ indicating no strong interactions with the S4 pocket. MPI60 showed also two possible confirmations at its *N*-terminal group but preferentially adopted a conformation not bound in the M^Pro^ S4 pocket. A similar flexible *N*-terminal group has been observed in other M^Pro^-dipeptidyl inhibitor complexes as well.^14,19^ For future designs of dipeptidyl inhibitors, novel *N*-terminal groups that can better engage the S4 pocket will likely improve the binding affinity to M^Pro^ and require innovative inputs.

## Conclusions

We have systematically surveyed reversibly covalent dipeptidyl M^Pro^ inhibitors on their characteristics including enzymatic inhibition, crystal structures of their complexes with M^Pro^, cellular and antiviral potency, cytotoxicity, and *in vitro* metabolic stability. Our results showed that the M^Pro^ S2 pocket is flexible in accommodating large P2 residues in dipeptidyl M^Pro^ inhibitors and inhibitors with two large P2 spiro residues, (*S*)-2-azaspiro[4,4]nonane-3-carboxylate (**a1**) and (*S*)-2-azaspiro[4,5]decane-3-carboxylate (**a2**), are favorable on most characteristics. One compound, MPI60, displayed the most favorable characteristics, suggesting that it can be considered for the next level of preclinical assessment.

## Experimental Section

### Materials

HEK293T/17 cells were from ATCC; DMEM with high glucose with GlutaMAX supplement, fetal bovine serum, 0.25% trypsin–EDTA, phenol red, and dimethyl sulfoxide were purchased from Thermo Fisher Scientific; linear polyethylenimine MW 25000 was from Polysciences. Spiro residues were purchased from Enamine (cat# EN300-1722865).

### *In Vitro* M^Pro^ Inhibition Potency Characterizations of Inhibitors

For most inhibitors, the assay was conducted using 20 nM M^Pro^ and 10 μM Sub3. We dissolved all inhibitors in DMSO as 10 mM stock solutions. Sub3 was dissolved in DMSO as a 1 mM stock solution and diluted 100 times in the final assay buffer containing 10 mM Na_*x*_H_*y*_PO_4_, 10 mM NaCl, 0.5 mM EDTA, and 1.25% DMSO at pH 7.6. M^Pro^ and an inhibitor were incubated in the final assay buffer for 30 min before adding the substrate to initiate the reaction catalyzed by M^Pro^. The production format was monitored in a fluorescence plate reader with excitation at 336 nm and emission at 490 nm. More assay details can be found in a previous study.^36^

### Cellular M^Pro^ Inhibition Potency Characterizations of Inhibitors

Cellular M^Pro^ inhibition potency for all tested inhibitors was characterized according to the protocol shown in a previous report.^35^ HEK 293T/17 cells were grown in high-glucose DMEM with GlutaMAX supplement and 10% fetal bovine serum in 10 cm culture plates under 37 °C and 5% CO_2_ to ∼80–90% and then transfected with the pLVX-MProeGFP-2 plasmid. For each transfection, 30 mg/mL polyethylenimine and a total of 8 μg of the plasmid in 500 μL of the Opti-MEM medium were used. Cells were incubated with transfecting reagents overnight. On the second day, the medium was removed, and cells were washed with a PBS buffer and then digested with 0.05% trypsin–EDTA. Cells were collected by centrifugation and then resuspended in the original growth medium to a cell density of 5 × 10^5^ cells/mL in 500 μL in a 48-well plate. A compound solution of 100 μL was then added to the growth medium. These cells were incubated under 37 °C and 5% CO_2_ for 3 days before their flow cytometry analysis.

### Recombinant M^Pro^ Protein Expression and Purification

The expression and purification were conducted according to the procedure in one previous report.^22^ The expression plasmid pET28a-His-SUMO-MPro was constructed in a previous study. We used this construct to transform *E. coli* BL21(DE3) cells. A single colony grown on an LB plate containing 50 μg/mL kanamycin was picked and grown in 5 mL LB medium supplemented with 50 μg/mL kanamycin overnight. We inoculated this overnight culture to 6 L 2YT medium with 50 μg/mL kanamycin. Cells were grown to OD_600_ as 0.8. At this point, we added 1 mM IPTG to induce the expression of His-SUMO-M^Pro^. Induced cells were allowed to grow for 3 h and then harvested by centrifugation at 12,000 rpm at 4 °C for 30 min. We resuspended cell pellets in 150 mL lysis buffer (20 mM Tris–HCl, 100 mM NaCl, 10 mM imidazole, pH 8.0) and lysed the cells by sonication on ice. We clarified the lysate by centrifugation at 16,000 rpm at 4 °C for 30 min. We decanted the supernatant and mixed it with Ni-NTA resins (GenScript). We loaded the resins to a column, washed the resins with 10 vol of lysis buffer, and eluted the bound protein using elution buffer (20 mM Tris–HCl, 100 mM NaCl, 250 mM imidazole, pH 8.0). We then exchanged the buffer of the elute to another buffer (20 mM Tris–HCl, 100 mM NaCl, 10 mM imidazole, 1 mM DTT, pH 8.0) using a HiPrep 26/10 desalting column (Cytiva) and digested the elute using 10 units SUMO protease overnight at 4 °C. The digested elute was subjected to Ni-NTA resins in a column to remove His-tagged SUMO protease, His-tagged SUMO tag, and undigested His-SUMO-M^Pro^. We then loaded the flow-through onto a Q-Sepharose column and purified M^Pro^ using FPLC by running a linear gradient from 0 to 500 mM NaCl in a buffer (20 mM Tris–HCl, 1 mM DTT, pH 8.0). M^Pro^ fractions eluted from the Qsepharose column were concentrated and loaded onto a HiPrep 16/60 Sephacryl S-100 HR column and exchanged with a buffer containing 20 mM Tris–HCl, 100 mM NaCl, 1 mM DTT, and 1 mM EDTA at pH 7.8. The final purified M^Pro^ was concentrated and stored in a −80 °C freezer.

### Cytotoxicity Assay of M^Pro^ Inhibitors

To assess the half-maximal cytotoxic concentration (CC50), stock solutions of the tested compounds were dissolved in DMSO to a final concentration of 10 mM and diluted further to the working concentrations in DMEM. HEK293T cells were seeded in 96-well plates and incubated at 37 °C and 5% CO_2_ for 24 h. The cells were then treated with different concentrations (200, 100, 50, 25, 12.5, 6.25, 3.125, 1.5625, 0.78125, and 0 μM) of the tested compound in triplicate for 48 h. Cell viability was assessed by the MTT assay to determine CC_50_. For the MTT assay, 20 μL MTT (5 mg/mL) was added to each well and incubated with cells for 4 h. After that, the supernatant was removed, and 200 μL DMSO was added per well. The absorbance was recorded at 490 nm. The CC50 value was determined by plotting the normalized cell viability (%) against the compound concentration.

### *In Vitro* Metabolic Stability in Human Liver Microsomes

The metabolic stability parameters of an inhibitor, including CL_int_ and half-life (*t*_1/2_), were determined by the estimation of the remaining compound levels after different time periods of incubation with human liver microsome, NADPH (cofactor), EDTA, and MgCl_2_ in a 0.1 M phosphate buffer (pH 7.4). Each inhibitor (5 μM) was preincubated with 40 μL of human liver microsome (0.5 mg/mL) in 0.1 M phosphate buffer (pH 7.4) at 37 °C for 5 min. After preincubation, NADPH (5 mM, 10 μL) or 0.1 M phosphate buffer (10 μL) was added to initiate metabolic reactions at 37 °C. All reactions were conducted in triplicate. At 0, 5, 15, 30, 45, and 60 min, 200 μL acetonitrile (with internal standard diclofenac, 10 μg/mL) was added to quench the metabolic reactions. The samples were subjected to centrifugation at 4 °C for 20 min at 4000 rpm. Then, 50 μL of clear supernatants was analyzed by HPLC-MS/MS. The percentage of the remaining compound with respect to the initial added level was determined by the following formula: % remaining = (area at *t_x_*/average area at *t*_0_) × 100. The half-life (*t*_1/2_) was calculated using the slope (*k*) of the log-linear regression from the % remaining of the parent compound versus time (min): *t*_1/2_ (min) = −ln 2/*k*. CL_int_ (mL/min/kg) was calculated using the formula CL_int_ = (0.693/*t*_1/2_) × (1/(microsomal protein concentration (0.5 mg/mL)) × scaling factor (1254.16 for human liver microsome).

### X-ray Crystallography Analysis

The crystallography analysis of M^Pro^ bound with three inhibitors was conducted according to a previous report.^20^ The sitting drop method was used to crystalize 14 mg/mL M^Pro^. One microliter of M^Pro^ in a buffer containing 20 mM Tris–HCl, 100 mM NaCl, 1 mM DTT, and 1 mM EDTA at pH 7.8 was mixed with 1 μL of a reservoir solution containing 0.2 M dibasic ammonium phosphate and 17% w/v PEG3350 at pH 8.0. Protein crystals appeared overnight. Soaking was performed to produce M^Pro^-inhibitor complex crystals. Overnight-grown M^Pro^ crystals were washed with the reservoir solution three times in situ. Subsequently, the crystals were washed three times with the reservoir solution plus 0.8 mM inhibitor and 2% DMSO (inhibitors were dissolved to 40 mM in 100% DMSO). The mixture was incubated at 25 °C for 48 h. The cryoprotectant solution contained the mother liquor plus 30% glycerol, 0.8 mM inhibitor, and 2% DMSO. Cryoprotected crystals were fished for data collection. The data of M^pro^ with MPI48, MPI49, MI-09, and MPI60 were collected on a Bruker Photon II detector. The diffraction data were indexed, integrated, and scaled using PROTEUM3. All the structures were determined by molecular replacement using the apo M^pro^ (PDB ID: 7JPY) as the search model by Phaser in the Phenix package. Jligand and Sketcher from the CCP4 suite were employed for the generation of structural coordinates and geometric restraints for the inhibitors. The inhibitors were built into the 2Fo-Fc density by using Coot. Refinement of all the structures was performed with Real-space Refinement in Phenix. Details of data quality and structure refinement are summarized in Table S1. All structural figures were generated with PyMOL.

### Compound Synthesis

All compounds were synthesized according to the synthetic routes presented in Scheme 1 by following the procedures described below.

#### General Procedure A

To a solution of **a** (e.g., MPI48a shown in Scheme 1 and all following **b–k** are named in a same way, 1 equiv) and **b** (1 equiv) in anhydrous DMF was added DIPEA (4 equiv), and the solution was cooled to 0 °C. HATU (1.2 equiv) was added to the solution under 0 °C and then stirred at rt overnight. The reaction mixture was then diluted with ethyl acetate and washed with saturated NaHCO_3_ solution (two times), 1 M HCl solution (two times), and saturated brine solution (two times) sequentially. The organic layer was dried over anhydrous Na_2_SO_4_ and then concentrated *in vacuo*. The residue was then purified with flash chromatography (50–100% EtOAc in hexanes as the eluent) to afford **c** as a white solid/gummy solid.

#### General Procedure B

The compound **c** (1 equiv) was dissolved in THF/H_2_O (1:1). LiOH (2.5 equiv) was added at 0 °C. The mixture was stirred at rt overnight. Then, THF was removed *in vacuo*, and the aqueous layer was acidified with 1 M HCl and extracted with dichloromethane (three times). The organic layer was dried over anhydrous Na_2_SO_4_ and concentrated to yield **d** as a white solid/gummy solid that was proceeded to the next step without further purification.

#### General Procedure C

To a solution of **d** (1 equiv) and **Int.i** (1 equiv) in anhydrous DMF was added DIPEA (4 equiv), and the mixture was cooled to 0 °C. HATU (1.2 equiv) was added to the solution under 0 °C and then stirred at rt overnight. The reaction mixture was then diluted with ethyl acetate and washed with saturated NaHCO_3_ solution (two times), 1 M HCl solution (two times), and saturated brine solution (two times) sequentially. The organic layer was dried over anhydrous Na_2_SO_4_ and then concentrated *in vacuo*. The residue was then purified with flash chromatography (0–10% MeOH in dichloromethane as the eluent) to afford **e** as a white solid/gummy solid.

#### General Procedure D

To a stirred solution of compound **e** (1 equiv) in THF was added LiBH_4_ (2.0 M in THF, 5 equiv) in several portions at 0 °C under a nitrogen atmosphere. The reaction mixture was stirred at 0 °C for 1 h, allowed to warm up to rt, and then stirred for an additional 2 h. The reaction was quenched by the dropwise addition of 1.0 M HCl (aq.) (1.2 mL) with cooling in an ice bath. The solution was diluted with ethyl acetate and H_2_O. The phases were separated, and the aqueous layer was extracted with ethyl acetate (three times). The organic phases were combined, dried over MgSO_4_, filtered, and concentrated on a rotorvap to give a yellow oily residue. Column chromatographic purification of the residue (2–10% MeOH in CH_2_Cl_2_ as the eluent) afforded **f** as a white solid/gummy solid.

#### General Procedure E

To a solution of **f** in CH_2_Cl_2_ was added NaHCO_3_ (4 equiv) and the Dess–Martin reagent (3 equiv). The resulting mixture was stirred at rt for 12 h. Then, the reaction was quenched with a saturated NaHCO_3_ solution containing 10% Na_2_S_2_O_3_. The layers were separated. The organic layer was then washed with saturated brine solution, dried over anhydrous Na_2_SO_4_, and concentrated *in vacuo*. The residue was then purified with flash chromatography to afford a final inhibitor compound as a white solid.

#### General Procedure F

Intermediate **h** was synthesized according to general procedure C from **g** and **int.ii**. **h** was used to make **i**. To a stirred solution of **h** (1 equiv) in 1,4-dioxane at 0 °C was added 4 N HCl (10 equiv). The reaction mixture was stirred at rt for 3 h. After completion of the reaction, solvent was concentrated in a vacuum. The residue **I** was used in the next step without further purification.

#### General Procedure G

There were two routes used to generate intermediate **k**. One was to follow general procedure A to synthesize **k** from **i** and **j**. The other was to follow the procedure described below. To a stirred solution of **i** (1 equiv) in THF at 0 °C was added DIPEA (2 equiv). After 15 min, Cbz-Cl (1.2 equiv) was added, and the mixture was stirred at rt for 3 h. The reaction was quenched with water (5 mL), and the mixture was concentrated in a vacuum. The residue was partitioned between EtOAc (10 mL) and H_2_O (5 mL). The aqueous layer was extracted with EtOAc (two times). The combined organic layer was washed with brine, dried over MgSO_4_, and concentrated in a vacuum. The residue was then purified with flash chromatography (0–10% MeOH in CH_2_Cl_2_ as the eluent) to afford **k** as a yellow liquid.

#### General Procedure H

To a stirred solution of **K** (1 equiv) in DCM (10 mL) at 0 °C was added Burgess reagent (2.5 equiv), and the mixture was stirred at rt for 2 h. The reaction was quenched with saturated NaHCO_3_ solution (5 mL) and extracted with DCM (2 × 10 mL). The combined organic layer was washed with brine, dried over MgSO_4_, and concentrated in a vacuum. The residue was then purified with flash chromatography (0–10% MeOH in dichloromethane as the eluent) to afford MPI66-1-67 as a white solid.

Characterizations of final inhibitors are presented below. The purity of the final compounds was assessed by LC–MS to confirm >95% purity. Data related to intermediates are in the Supplementary Information.

##### *N*-((*S*)-4,4-Dimethyl-1-oxo-1-(((*S*)-1-oxo-3-((*S*)-2-oxopyrrolidin-3-yl)propan-2-yl)amino)pentan-2-yl)-1*H*-indole-2-carboxamide (**MPI48**)

**MPI48** was prepared as a white solid following general procedure **E** (yield 45%). ^1^H NMR (400 MHz, DMSO-*d*_6_) δ 11.60 (s, 1H), 9.42 (s, 1H), 8.71–8.44 (m, 2H), 7.62 (d, *J* = 9.5 Hz, 2H), 7.42 (d, *J* = 8.3 Hz, 1H), 7.25 (s, 1H), 7.18 (t, *J* = 7.5 Hz, 1H), 7.03 (t, *J* = 7.5 Hz, 1H), 4.62 (td, *J* = 9.0, 3.3 Hz, 1H), 4.20 (ddt, *J* = 14.9, 11.4, 5.6 Hz, 1H), 3.19–2.95 (m, 2H), 2.39–2.21 (m, 1H), 2.21–2.06 (m, 1H), 1.97–1.88 (m, 1H), 1.88–1.72 (m, 2H), 1.72–1.55 (m, 2H), 0.94 (s, 9H). ^13^C NMR (101 MHz, DMSO) δ 201.3, 178.8, 173.8, 161.2, 136.9, 131.9, 127.5, 123.9, 122.0, 120.2, 112.7, 103.9, 60.2, 56.9, 51.1, 45.1, 37.8, 30.9, 30.1, 29.8, 27.8. MS (ESI): *m*/*z* = 425.23 [M – H]^−^.

##### *N*-((*S*)-4,4-Dimethyl-1-oxo-1-(((*S*)-1-oxo-3-((*S*)-2-oxopyrrolidin-3-yl)propan-2-yl)amino)pentan-2-yl)-4-methoxy-1*H*-indole-2-carboxamide (**MPI49**)

**MPI49** was prepared as a white solid following general procedure **E** (yield 72%). ^1^H NMR (400 MHz, chloroform-*d*) δ 10.85 (s, 1H), 9.69–9.50 (m, 1H), 9.41 (s, 1H), 8.41 (d, *J* = 6.1 Hz, 1H), 7.15–7.09 (m, 1H), 7.07–7.00 (m, 1H), 6.97–6.92 (m, 1H), 6.73 (dd, *J* = 18.8, 8.3 Hz, 1H), 6.42 (dd, *J* = 7.7, 3.6 Hz, 1H), 5.96 (s, 1H), 4.90–4.71 (m, 1H), 4.22 (ddt, *J* = 23.4, 11.1, 5.5 Hz, 1H), 3.87 (s, 3H), 3.29–2.90 (m, 2H), 2.44–2.17 (m, 2H), 2.03–1.79 (m, 3H), 1.72–1.63 (m, 2H), 0.94 (s, 9H). ^13^C NMR (101 MHz, CDCl3): δ 199.82, 180.19, 173.89, 161.58, 154.11, 138.50, 138.12, 128.96, 125.66, 118.81, 108.03, 99.61, 57.77, 55.30, 51.84, 50.84, 40.99, 37.63, 30.66, 29.86, 29.79, 29.72, 28.35. MS (ESI): *m*/*z* = 457.24 [M + H]^+^.

##### Benzyl ((*S*)-4,4-Dimethyl-1-oxo-1-(((*S*)-1-oxo-3-((*S*)-2-oxopyrrolidin-3-yl)propan-2-yl)amino)pentan-2-yl)carbamate (**MPI50**)

**MPI50** was prepared as a white solid following general procedure **E** (yield 25%). ^1^H NMR (400 MHz, chloroform-*d*) δ 9.46 (s, 1H), 8.01 (d, *J* = 7.7 Hz, 1H), 7.28 (d, *J* = 4.1 Hz, 5H), 6.71 (d, *J* = 7.4 Hz, 1H), 6.28–6.15 (m, 1H), 5.41 (s, 1H), 5.08–4.92 (m, 2H), 4.45–4.33 (m, 1H), 4.17 (d, *J* = 10.3 Hz, 1H), 3.26–3.07 (m, 2H), 2.28 (d, *J* = 16.7 Hz, 2H), 2.01 (p, *J* = 7.4, 6.7 Hz, 2H), 1.89 (d, *J* = 14.1 Hz, 1H), 1.74 (dd, *J* = 45.0, 9.9 Hz, 2H), 1.65 (s, 1H), 0.88 (s, 9H). ^13^C NMR (100 MHz, chloroform-*d*) δ 200.61, 173.84, 173.18, 156.24, 136.07, 128.63, 128.35, 128.17, 67.27, 63.36, 57.13, 51.84, 45.79, 40.34, 34.88, 30.66, 30.24, 29.67, 28.12, 17.37, 17.10. MS (ESI): *m*/*z* = 418.23 [M + H]^+^.

##### Benzyl ((*S*)-3-Cyclohexyl-1-oxo-1-(((*S*)-1-oxo-3-((*S*)-2-oxopyrrolidin-3-yl)propan-2-yl)amino)propan-2-yl)carbamate (**MPI51**)

**MPI51** was prepared as a white solid following general procedures **D** and **E** (yield 47%). ^1^H NMR (400 MHz, DMSO-*d*_6_) δ 9.40 (s, 1H), 8.48 (d, *J* = 7.6 Hz, 1H), 7.63 (s, 1H), 7.47 (d, *J* = 8.1 Hz, 1H), 7.38–7.27 (m, 5H), 5.03 (s, 2H), 4.18 (ddd, *J* = 11.4, 7.4, 4.0 Hz, 0H), 4.10 (q, *J* = 7.8 Hz, 1H), 3.24–2.96 (m, 3H), 2.36–2.21 (m, 1H), 2.21–2.04 (m, 1H), 1.95–1.83 (m, 1H), 1.77–1.53 (m, 6H), 1.53–1.40 (m, 2H), 1.39–1.27 (m, 1H), 1.25–1.01 (m, 4H), 0.98–0.76 (m, 2H). ^13^C NMR (101 MHz, CDCl3) δ 199.9, 180.3, 173.9, 156.3, 136.5, 128.6, 128.3, 128.1, 67.0, 57.9, 40.8, 40.7, 38.3, 34.2, 33.8, 32.6, 29.8, 28.8, 26.5, 26.3, 26.2.

##### 3-Chlorobenzyl ((*S*)-3-Cyclohexyl-1-oxo-1-(((*S*)-1-oxo-3-((*S*)-2-oxopyrrolidin-3-yl)propan-2-yl)amino)propan-2-yl)carbamate (**MPI52**)

**MPI52** was prepared as a white solid following general procedure **E** (yield 80%). ^1^H NMR (400 MHz, chloroform-*d*) δ 9.41 (s, 1H), 8.33 (d, *J* = 5.9 Hz, 1H), 7.42–7.03 (m, 4H), 6.27 (s, 1H), 5.47 (d, *J* = 8.5 Hz, 1H), 5.01 (s, 2H), 4.42–4.18 (m, 2H), 3.40–3.14 (m, 2H), 2.45–2.27 (m, 2H), 1.94–1.70 (m, 4H), 1.65–1.55 (m, 5H), 1.51–1.42 (m, 1H), 1.35–1.29 (m, 1H), 1.19–1.04 (m, 3H), 0.96–0.80 (m, 2H).^13^C NMR (101 MHz, CDCl_3_) δ 199.74, 180.22, 173.77, 155.88, 138.55, 134.36, 129.82, 128.19, 127.80, 125.83, 67.98, 65.92, 57.94, 40.77, 40.68, 38.33, 34.09, 33.67, 32.52, 29.67, 28.73, 26.38, 26.21, 26.03. MS (ESI): *m*/*z* = 478.21 [M + H]^+^.

##### 3-(((((*S*)-3-Cyclohexyl-1-oxo-1-(((*S*)-1-oxo-3-((*S*)-2-oxopyrrolidin-3-yl)propan-2-yl)amino)propan-2-yl)carbamoyl)oxy)methyl)phenyl Acetate (**MPI53**)

**MPI53** was prepared as a white solid following general procedure **E** (yield 80%). ^1^H NMR (400 MHz, chloroform-*d*) δ 9.47 (s, 1H), 8.36 (d, *J* = 5.9 Hz, 1H), 7.34 (t, *J* = 7.9 Hz, 1H), 7.20 (d, *J* = 7.7 Hz, 1H), 7.10 (s, 1H), 7.02 (ddd, *J* = 8.1, 2.4, 1.0 Hz, 1H), 6.25 (d, *J* = 14.9 Hz, 1H), 5.47 (d, *J* = 8.5 Hz, 1H), 5.10 (s, 2H), 4.43–4.25 (m, 2H), 3.37–3.23 (m, 2H), 2.50–2.32 (m, 2H), 2.29 (s, 3H), 2.04–1.77 (m, 4H), 1.75–1.61 (m, 5H), 1.53 (ddd, *J* = 14.0, 9.2, 5.3 Hz, 1H), 1.38 (s, 1H), 1.26–1.11 (m, 3H), 0.94 (dd, *J* = 23.0, 11.7 Hz, 2H). MS (ESI): *m*/*z* = 502.25 [M + H]^+^.

##### Benzyl ((2*S*,3*S*)-3-(*tert*-Butoxy)-1-oxo-1-(((*S*)-1-oxo-3-((*S*)-2-oxopyrrolidin-3-yl)propan-2-yl)amino)butan-2-yl)carbamate (**MPI54**)

**MPI54** was prepared as a white solid following general procedure **E** (yield 91%). ^1^H NMR (400 MHz, chloroform-*d*) δ 9.4 (s, 1H), 8.1 (d, *J* = 6.6 Hz, 1H), 7.3–7.2 (m, 5H), 6.7–6.5 (m, 1H), 6.1–5.9 (m, 1H), 5.1–4.9 (m, 2H), 4.3 (s, 2H), 4.0–3.9 (m, 1H), 3.2 (d, *J* = 8.3 Hz, 2H), 2.5–2.3 (m, 1H), 2.2 (dd, *J* = 14.5, 6.6 Hz, 1H), 2.0–1.9 (m, 1H), 1.9–1.6 (m, 2H), 1.1 (s, 12H). ^13^C NMR (100 MHz, chloroform-*d*) δ 199.8, 180.1, 171.2, 156.4, 136.2, 128.6, 128.3, 128.2, 74.7, 67.9, 67.3, 61.4, 55.1, 50.8, 40.6, 38.1, 29.9, 28.3, 19.6. MS (ESI): *m*/*z* = 448.24 [M + H]^+^.

##### Synthesis of (1*R*,2*S*,5*S*)-3-(2-(2,4-Dichlorophenoxy)acetyl)-6,6-dimethyl-*N*-((*S*)-1-oxo-3-((*S*)-2-oxopyrrolidin-3-yl)propan-2-yl)-3-azabicyclo[3.1.0]hexane-2-carboxamide (**MPI55**)

**MPI55** was prepared as a white solid following general procedure **E** (yield 56%). ^1^H NMR (400 MHz, chloroform-*d*) δ 9.40 (dd, *J* = 5.3, 1.0 Hz, 1H), 7.27 (t, *J* = 2.7 Hz, 1H), 7.10–7.00 (m, 1H), 6.80 (dd, *J* = 8.9, 6.3 Hz, 1H), 4.64 (s, 2H), 4.22–4.09 (m, 1H), 3.95–3.77 (m, 1H), 3.59 (d, *J* = 10.4 Hz, 1H), 3.35–3.18 (m, 2H), 2.49–2.36 (m, 1H), 2.36–2.19 (m, 1H), 2.00–1.63 (m, 3H), 1.55–1.37 (m, 6H), 0.98 (d, *J* = 2.0 Hz, 3H), 0.82 (d, *J* = 14.1 Hz, 3H). MS (ESI): *m*/*z* = 460.24 [M + H]^+^.

##### (2*S*,4*S*)-Benzyl 4-Cyclohexyl-2-(((*S*)-1-oxo-3-((*S*)-2-oxopyrrolidin-3-yl)propan-2-yl)carbamoyl)pyrrolidine-1-carboxylate (**MPI56**)

**MPI56** was prepared as a white solid following general procedure **E** (yield 65%). ^1^H NMR (400 MHz, chloroform-*d*) δ 9.50 (s, 0.5H), 9.16 (s, 0.5H), 8.65–8.45 (m, 0.5H), 8.12 (d, *J* = 6.3 Hz, 0.5H), 7.43–7.27 (m, 5H), 6.39 (s, 0.5H), 6.11 (s, 0.5H), 5.32–5.23 (m, 0.5H), 5.14 (t, *J* = 3.7 Hz, 1H), 5.01 (d, *J* = 12.4 Hz, 0.5H), 4.50–4.27 (m, 2H), 4.13 (d, *J* = 6.7 Hz, 0.5H), 3.75 (tdd, *J* = 11.8, 9.0, 8.1, 4.5 Hz, 1H), 3.39–3.15 (m, 3.5H), 3.06 (dt, *J* = 29.1, 10.0 Hz, 1H), 2.53–2.16 (m, 3H), 2.16–1.54 (m, 11H), 1.28–1.06 (m, 5H), 1.00–0.83 (m, 2H). ^13^C NMR (101 MHz, CDCl_3_) δ 200.09, 199.79, 179.99, 179.88, 173.91, 173.24, 155.68, 154.81, 136.61, 128.47, 128.05, 127.93, 67.05, 61.05, 57.12, 55.08, 51.16, 50.80, 43.71, 41.71, 40.52, 37.99, 31.89, 31.41, 26.29, 26.02.

##### Synthesis of Benzyl (1*R*,2*S*,5*S*)-6,6-Dimethyl-2-((1-oxo-3-(2-oxopyrrolidin-3-yl)propan-2-yl)carbamoyl)-3-azabicyclo[3.1.0]hexane-3-carboxylate (**MPI57**)

**MPI57** was prepared as a white solid following general procedure **E** (yield 58%). ^1^H NMR (400 MHz, chloroform-*d*) δ 9.44 (d, *J* = 0.8 Hz, 0H), 9.05 (d, *J* = 1.5 Hz, 0H), 7.37–7.15 (m, 5H), 5.26–5.13 (m, 1H), 5.12–5.00 (m, 1H), 4.44–4.24 (m, 1H), 4.14 (d, *J* = 6.7 Hz, 1H), 4.08–3.95 (m, 1H), 3.74–3.66 (m, 1H), 3.56–3.44 (m, 1H), 3.30–3.21 (m, 2H), 2.47–2.09 (m, 2H), 2.00–1.85 (m, 1H), 1.85–1.60 (m, 3H), 1.52–1.16 (m, 3H), 0.97 (d, *J* = 2.7 Hz, 3H), 0.86 (s, 3H). ^13^C NMR (100 MHz, chloroform-*d*) δ 200.16, 199.86, 180.09, 173.51, 172.89, 154.55, 153.94, 136.65, 136.58, 128.46, 128.45, 128.05, 128.00, 127.96, 127.59, 67.13, 67.05, 61.45, 61.40, 58.49, 57.93, 50.85, 47.26, 40.73, 40.59, 38.67, 38.09, 32.96, 31.51, 29.55, 29.08, 26.32, 26.24, 26.16, 19.33, 19.22, 12.63, 12.55. MS (ESI): *m*/*z* = 428.21 [M + H]^+^.

##### Synthesis of Benzyl (*S*)-6-(((*S*)-1-oxo-3-((*S*)-2-oxopyrrolidin-3-yl)propan-2yl)carbamoyl)-5-azaspiro[2,4]heptane-5-carboxylate (**MPI58**)

**MPI58** was prepared as a white solid following general procedure **E** (yield 66%). ^1^H NMR (400 MHz, chloroform-*d*) δ 9.57 (d, *J* = 25.9 Hz, 1H), 9.20 (s, 0H), 8.61–8.37 (m, 0H), 8.23–7.98 (m, 1H), 7.34 (dt, *J* = 12.7, 6.5 Hz, 6H), 6.13–5.81 (m, 1H), 5.34–5.01 (m, 2H), 4.52–4.22 (m, 3H), 3.78 (ddd, *J* = 17.3, 10.6, 6.0 Hz, 1H), 3.47–3.20 (m, 3H), 2.62–1.63 (m, 9H), 1.19 (s, 9H). ^13^C NMR (100 MHz, chloroform-*d*) δ 199.67, 179.99, 173.03, 136.60, 128.47, 127.99, 127.72, 74.05, 69.47, 59.36, 37.45, 28.27. MS (ESI): *m*/*z* = 414.20 [M + H]^+^.

##### Benzyl (*S*)-7-(((*S*)-1-Oxo-3-((*S*)-2-oxopyrrolidin-3-yl)propan-2-yl)carbamoyl)-6-azaspiro[3.4]octane-6-carboxylate (**MPI59**) (Diastereomers)

**MPI59** was prepared as a white solid following general procedure **E** (yield 67%). ^1^H NMR (400 MHz, CDCl3) δ 9.44 (s, 0.5H), 9.02 (s, 0.5H), 8.69–8.63 (m, 0.5H), 8.17 (d, *J* = 6.4 Hz, 0.5H), 7.33–7.14 (m, 5H), 6.47–6.42 (m, 0.5H), 6.06–6.00 (m, 0.5H), 5.34–4.86 (m, 2H), 4.28 (dd, *J* = 7.8, 5.9 Hz, 1.5H), 4.05–3.93 (m, 0.5H), 3.55–3.44 (m, 2H), 3.26 (p, *J* = 8.1 Hz, 2H), 2.57–2.04 (m, 8H), 1.95–1.60 (m, 9H).

##### Benzyl 3-(((*S*)-1-Oxo-3-((*S*)-2-oxopyrrolidin-3-yl)propan-2-yl)carbamoyl)-2-azaspiro[4.4]nonane-2-carboxylate (**MPI60**)(1:1 Diastereomers)

**MPI60** was prepared as a white solid following general procedure **E** (yield 60%). ^1^H NMR (400 MHz, CDCl3) δ 9.44 (s, 0.5H), 9.04 (s, 0.5H), 8.58 (s, 0.5H), 8.14 (s, 0.5H), 7.31–7.18 (m, 5H), 6.23 (s, 0.5H), 5.94 (s, 0.5H), 5.34–4.88 (m, H), 4.28 (t, *J* = 7.7 Hz, 1.5H), 4.03–3.97 (m, 0.5H), 3.57–3.34 (m, 1H), 3.31–3.23 (m, 3H), 2.58–2.27 (m, 1H), 2.23–2.07 (m, 1H), 1.94–1.36 (m, 13H). HRMS (ESI) *m*/*z*: calcd for C_24_H_32_N_3_O_5_ [M + H]^+^ 442.23, found 442.23.

##### Benzyl 3-(((*S*)-1-Oxo-3-((*S*)-2-oxopyrrolidin-3-yl)propan-2-yl)carbamoyl)-2-azaspiro[4.5]decane-2-carboxylate (**MPI61**)

**MPI61** was prepared as a white solid following general procedure **E** (yield 58%). ^1^H NMR (400 MHz, DMSO) δ 9.55–8.92 (m, 1H), 8.49 (dd, *J* = 12.5, 7.2 Hz, 1H), 7.56 (d, *J* = 10.1 Hz, 1H), 7.42–6.98 (m, 5H), 5.08–4.81 (m, 2H), 4.30–3.95 (m, 2H), 3.38 (t, *J* = 12.6 Hz, 1H), 3.14–2.76 (m, 3H), 2.32–0.96 (m, 17H). HRMS (ESI) *m*/*z*: calcd for C_25_H_34_N_3_O_5_ [M + H]^+^ 456.25, found 456.25.

##### Synthesis of (1*R*,2*S*,5*S*)-3-((4-Chlorophenyl)glycyl)-6,6-dimethyl-*N*-((*S*)-1-oxo-3-((*S*)-2-oxopyrrolidin-3-yl)propan-2-yl)-3-azabicyclo[3.1.0]hexane-2-carboxamide (**MPI62**)

**MPI62** was prepared with **MPI62d** and (*S*)-2-amino-3-((*S*)-2-oxopyrrolidin-3-yl)propanal as a white solid following general procedure **C** (yield 35%).

##### Synthesis of (*S*)-5-(2-(2,4-Dichlorophenoxy)acetyl)-*N*-((*S*)-1-oxo-3-((*S*)-2-oxopyrrolidin-3-yl)propan-2-yl)-5-azaspiro[2.4]heptane-6-carboxamide (**MPI63**)

**MPI63** was prepared as a white solid following general procedure **E** (yield 54%). ^1^H NMR (400 MHz, chloroform-*d*) δ 9.44–9.32 (m, 1H), 8.29–8.14 (m, 1H), 7.99–7.76 (m, 2H), 7.66 (dt, *J* = 10.4, 7.4 Hz, 1H), 7.28 (dd, *J* = 6.6, 2.7 Hz, 1H), 7.13–7.01 (m, 1H), 7.01–6.77 (m, 2H), 4.80–4.70 (m, 2H), 4.70–4.63 (m, 1H), 4.25–4.04 (m, 1H), 3.70–3.49 (m, 2H), 3.43 (d, *J* = 9.7 Hz, 1H), 3.36–3.21 (m, 3H), 3.09–2.99 (m, 1H), 2.48–2.39 (m, 1H), 2.39–2.25 (m, 2H), 1.93–1.73 (m, 4H), 1.39 (dd, *J* = 19.1, 7.0 Hz, 2H), 0.80 (q, *J* = 7.2, 6.8 Hz, 2H), 0.68–0.37 (m, 5H). MS (ESI): *m*/*z* = 482.12 [M + H]^+^.

##### (1*R*,2*S*,5*S*)-3-(2-(Cyclohexyloxy)acetyl)-6,6-dimethyl-*N*-((*S*)-1-oxo-3-((*S*)-2-oxopyrrolidin-3-yl)propan-2-yl)-3-azabicyclo[3.1.0]hexane-2-carboxamide (**MPI64**)

**MPI64** was prepared as a white solid following general procedures **D** and **E** (yield 60%). ^1^H NMR (400 MHz, CDCl_3_) δ 9.44 (s, 1H), 8.12 (d, *J* = 5.9 Hz, 1H), 5.84 (s, 1H), 4.32 (s, 1H), 4.26–4.12 (m, 1H), 4.09–3.95 (m, 2H), 3.82–3.69 (m, 1H), 3.50 (d, *J* = 10.5 Hz, 1H), 3.34–3.25 (m, 3H), 2.52–2.31 (m, 2H), 1.95–1.82 (m, 4H), 1.50–1.40 (m, 3H), 1.31–1.11 (m, 6H), 0.98 (s, 3H), 0.86 (s, 3H). MS (ESI): *m*/*z* = 434.26 [M + H]^+^.

##### (1*R*,2*S*,5*S*)-3-(3-Cyclohexylpropanoyl)-6,6-dimethyl-*N*-((*S*)-1-oxo-3-((*S*)-2-oxopyrrolidin-3-yl)propan-2-yl)-3-azabicyclo[3.1.0]hexane-2-carboxamide (**MPI65**)

**MPI65** was prepared as a white solid following general procedure **E** (yield 80%). ^1^H NMR (400 MHz, CDCl_3_) δ 9.45 (d, *J* = 0.8 Hz, 1H), 8.13 (d, *J* = 6.2 Hz, 1H), 6.09 (s, 1H), 4.29 (d, *J* = 9.5 Hz, 2H), 3.80 (dd, *J* = 10.3, 5.2 Hz, 1H), 3.42 (d, *J* = 10.3 Hz, 1H), 3.38–3.22 (m, 3H), 2.48 (ddd, *J* = 13.5, 6.5, 4.4 Hz, 1H), 2.33 (dddd, *J* = 12.1, 8.8, 6.0, 3.1 Hz, 1H), 2.27–2.04 (m, 3H), 2.01–1.69 (m, 3H), 1.67–1.51 (m, 4H), 1.49–1.38 (m, 4H), 1.21–1.05 (m, 4H), 0.99 (s, 3H), 0.87 (s, 3H), 0.79 (d, *J* = 10.8 Hz, 2H).

##### Benzyl 3-(((*S*)-1-Cyano-2-((*S*)-2-oxopyrrolidin-3-yl)ethyl)carbamoyl)-2-azaspiro[4.4]nonane-2-carboxylate (**MPI66-1**)

**MPI66-1** was prepared as a white solid following general procedure **H** (yield 52%). ^1^H NMR (400 MHz, DMSO) δ 8.98–8.75 (m, 1H), 7.70 (d, *J* = 3.9 Hz, 1H), 7.45–7.20 (m, 5H), 5.09–5.00 (m, 2H), 4.99–4.92 (m, 1H), 4.27–4.10 (m, 1H), 3.39 (d, *J* = 10.4 Hz, 1H), 3.25 (t, *J* = 10.4 Hz, 1H), 3.19–2.97 (m, 2H), 2.24–2.02 (m, 3H), 1.82–1.69 (m, 2H), 1.67–1.40 (m, 9H). HRMS (ESI) *m*/*z*: calcd for C_24_H_31_N_4_O_4_ [M + H]^+^ 439.23, found 439.23.

##### 3-Chlorobenzyl 3-(((*S*)-1-Cyano-2-((*S*)-2-oxopyrrolidin-3-yl)ethyl)carbamoyl)-2-azaspiro[4.4]nonane-2-carboxylate (**MPI66-2**)

**MPI66-2** was prepared as a white solid following general procedure **H** (yield 48%). ^1^H NMR (400 MHz, CDCl_3_) δ 8.59 (dd, *J* = 18.8, 6.1 Hz, 0.5H), 8.27 (dd, *J* = 75.9, 7.0 Hz, 0.5H), 7.29–7.10 (m, 3H), 6.57–6.07 (m, 1H), 5.13–4.88 (m, 2H), 4.82–4.54 (m, 1H), 4.31–4.09 (m, 1H), 3.50–3.35 (m, 1H), 3.35–3.06 (m, 3H), 2.57–1.64 (m, 7H), 1.63–1.26 (m, 8H). HRMS (ESI) *m*/*z*: calcd for C_24_H_30_ClN_4_O_4_ [M + H]^+^ 473.19, found 473.19.

##### 2-(4-Methoxy-1*H*-indole-2-carbonyl)-*N*-((*S*)-1-oxo-3-((*S*)-2-oxopyrrolidin-3-yl)propan-2-yl)-2-azaspiro[4.4]nonane-3-carboxamide (**MPI66-3**)

**MPI66-3** was prepared as a white solid following general procedure **E** (yield 48%). ^1^H NMR (400 MHz, CDCl_3_) δ 9.46 (s, 1H), 8.38–8.20 (m, 1H), 7.17–6.86 (m, 3H), 6.44 (d, *J* = 7.7 Hz, 1H), 5.57 (s, 0.5H), 5.10 (s, 0.5H), 4.77 (t, *J* = 7.7 Hz, 0.5H), 4.66 (t, *J* = 8.3 Hz, 0.5H), 4.31 (dd, *J* = 16.3, 9.3 Hz, 1H), 3.94–3.72 (m, 5H), 3.31–2.90 (m, 2H), 2.55–2.46 (m, 1H), 2.39–2.08 (m, 2H), 2.02–1.86 (m, 1H), 1.82–1.59 (m, 6H), 1.49 (d, *J* = 30.5 Hz, 5H).

##### *N*-((*S*)-1-Cyano-2-((*S*)-2-oxopiperidin-3-yl)ethyl)-2-(4-methoxy-1*H*-indole-2-carbonyl)-2-azaspiro[4.4]nonane-3-carboxamide (**MPI66-4**)

**MPI66-4** was prepared as a white solid following general procedure **H** (yield 59%). ^1^H NMR (400 MHz, DMSO) δ 11.57 (d, *J* = 2.3 Hz, 1H), 8.87 (d, *J* = 8.1 Hz, 1H), 7.53 (s, 1H), 7.12 (q, *J* = 8.0 Hz, 1H), 7.04 (d, *J* = 8.2 Hz, 1H), 6.94 (d, *J* = 2.3 Hz, 1H), 6.53 (d, *J* = 7.6 Hz, 1H), 5.05 (q, *J* = 7.9 Hz, 1H), 4.48 (dd, *J* = 9.2, 7.5 Hz, 1H), 3.90 (s, 3H), 3.82 (d, *J* = 5.3 Hz, 2H), 3.11 (qd, *J* = 7.3, 4.8 Hz, 2H), 2.35–2.22 (m, 2H), 2.13 (dd, *J* = 12.2, 7.4 Hz, 1H), 1.93–1.85 (m, 1H), 1.77 (dd, *J* = 9.3, 2.7 Hz, 3H), 1.67–1.55 (m, 7H), 1.49–1.33 (m, 3H). HRMS (ESI) *m*/*z*: calcd for C_27_H_34_N_5_O_4_ [M + H]^+^ 492.26, found 492.26.

##### Benzyl 3-(((*S*)-1-Cyano-2-((*S*)-2-oxopyrrolidin-3-yl)ethyl)carbamoyl)-2-azaspiro[4.5]decane-2-carboxylate (**MPI67**)

**MPI67** was prepared as a white solid following general procedure **H** (yield 62%). ^1^H NMR (400 MHz, DMSO) δ 8.80 (t, *J* = 7.8 Hz, 1H), 7.63 (s, 1H), 7.32–7.18 (m, 5H), 5.02–4.91 (m, 2H), 4.86 (dd, *J* = 15.2, 7.5 Hz, 1H), 4.12 (dt, *J* = 29.4, 8.1 Hz, 1H), 3.46–3.34 (m, 1H), 3.10–2.99 (m, 3H), 2.12–2.00 (m, 2H), 1.70–1.61 (m, 1H), 1.56–1.44 (m, 1H), 1.42–1.21 (m, 10H). HRMS (ESI) *m*/*z*: calcd for C_25_H_33_N_4_O_4_ [M + H]^+^ 453.25, found 453.25.

##### (*S*)-*N*-((*S*)-1-Cyano-2-((*S*)-2-oxopyrrolidin-3-yl)ethyl)-3-cyclohexyl-2-(2,2,2-trifluoroethanethioamido)propenamide (**VB-B-31**)

**VB-B-31** was prepared as a white solid following general procedure **H** (yield 62%). ^1^H NMR (400 MHz, CDCl_3_) δ 8.98 (d, *J* = 5.7 Hz, 1H), 6.23 (s, 1H), 4.98–4.79 (m, 1H), 4.66 (dt, *J* = 11.0, 5.3 Hz, 1H), 3.40–3.30 (m, 2H), 2.51–2.21 (m, 3H), 2.05–1.97 (m, 1H), 1.89–1.74 (m, 3H), 1.72–1.54 (m, 5H), 1.23–0.82 (m, 7H). MS (ESI): *m*/*z* = 403.19 [M + H]^+^.

##### *tert*-Butyl-(*S*)-7-(((*S*)-1-oxo-3-((*S*)-2-oxopyrrolidin-3-yl)propan-2-yl)carbamoyl)-6-azaspiro[3.4]octane-6-carboxylate (**YR-C-101**)

YR-B-101 was prepared as a white solid following general procedure **H** (yield 54%) (35 mg, 54%). ^1^H NMR (400 MHz, CDCl_3_) δ 9.51 (s, 1H), 4.28 (t, *J* = 6.8 Hz, 2H), 3.56–3.28 (m, 4H), 2.39 (s, 2H), 2.26–2.16 (m, 2H), 2.08–1.76 (m, 9H), 1.44 (s, 9H).

##### (1*R*,2*S*,5*S*)-6,6-Dimethyl-*N*-((*S*)-1-oxo-3-((*S*)-2-oxopyrrolidin-3-yl)propan-2-yl)-3-(2-(4-(trifluoromethoxy)phenoxy)acetyl)-3-azabicyclo[3.1.0]hexane-2-carboxamide (**MI09**)

**MI-09** was synthesized according to the literature. ^1^H NMR (400 MHz, chloroform-*d*) δ 9.48 (s, 1H), 8.39 (d, *J* = 5.8 Hz, 1H), 7.17–7.04 (m, 2H), 6.90 (d, *J* = 9.2 Hz, 2H), 6.08 (s, 1H), 4.69–4.56 (m, 2H), 4.42 (s, 1H), 4.34–4.25 (m, 1H), 3.94 (dd, *J* = 10.3, 4.6 Hz, 1H), 3.56 (d, *J* = 10.3 Hz, 1H), 3.35–3.21 (m, 2H), 2.57–2.47 (m, 1H), 2.38–2.27 (m, 1H), 1.96–1.86 (m, 2H), 1.83–1.73 (m, 1H), 1.63–1.52 (m, 2H), 1.06 (s, 3H), 0.90 (s, 3H). ^13^C NMR (101 MHz, CDCl_3_): δ 199.91, 180.39, 172.15, 166.45, 156.47, 143.32, 143.30, 122.44, 115.62, 67.38, 61.35, 58.16, 46.42, 40.63, 38.26, 30.76, 29.61, 28.84, 27.60, 26.17, 19.42, 12.61. MS (ESI): *m*/*z* = 512.20 [M + H]^+^.

##### (1*R*,2*S*,5*S*)-3-(2-(2,4-Dichlorophenoxy)acetyl)-6,6-dimethyl-*N*-((*S*)-1-oxo-3-((*S*)-2-oxopyrrolidin-3-yl)propan-2-yl)-3-azabicyclo[3.1.0]hexane-2-carboxamide (**MI-14**)

**MI-14** was synthesized according to the literature. ^1^H NMR (400 MHz, chloroform-*d*) δ 9.40 (dd, *J* = 5.3, 1.0 Hz, 1H), 7.27 (t, *J* = 2.7 Hz, 1H), 7.10–7.00 (m, 1H), 6.80 (dd, *J* = 8.9, 6.3 Hz, 1H), 4.64 (s, 2H), 4.22–4.09 (m, 1H), 3.95–3.77 (m, 1H), 3.59 (d, *J* = 10.4 Hz, 1H), 3.35–3.18 (m, 2H), 2.49–2.36 (m, 1H), 2.36–2.19 (m, 1H), 2.00–1.63 (m, 3H), 1.55–1.37 (m, 6H), 0.98 (d, *J* = 2.0 Hz, 3H), 0.82 (d, *J* = 14.1 Hz, 3H). MS (ESI): *m*/*z* = 496.13 [M + H]^+^.

##### (1*S*,3a*R*,6a*S*)-2-(2-(2,4-Dichlorophenoxy)acetyl)-*N*-((*S*)-1-oxo-3-((*S*)-2-oxopyrrolidin-3-yl)propan-2-yl)octahydrocyclopenta[*c*]pyrrole-1-carboxamide (**MI30**)

**MI-30** was synthesized according to the literature. ^1^H NMR (400 MHz, chloroform-*d*) δ 9.41 (d, *J* = 0.9 Hz, 1H), 8.24 (d, *J* = 6.0 Hz, 1H), 7.29 (d, *J* = 2.5 Hz, 1H), 7.07 (dd, *J* = 8.8, 2.6 Hz, 1H), 6.81 (d, *J* = 8.9 Hz, 1H), 6.14 (s, 1H), 4.76–4.63 (m, 2H), 4.33 (d, *J* = 3.0 Hz, 1H), 4.21 (ddd, *J* = 8.9, 7.3, 5.9 Hz, 1H), 3.81 (dd, *J* = 10.6, 7.9 Hz, 1H), 3.42 (dd, *J* = 10.4, 4.0 Hz, 1H), 3.22 (ddd, *J* = 15.9, 9.6, 7.2 Hz, 2H), 2.85–2.67 (m, 2H), 2.48–2.37 (m, 1H), 2.30–2.22 (m, 1H), 1.99–1.90 (m, 1H), 1.87–1.78 (m, 3H), 1.74–1.62 (m, 2H), 1.60–1.46 (m, 2H), 1.43–1.34 (m, 1H). ^13^C NMR (101 MHz, CDCl_3_): δ 199.85, 180.28, 172.70, 166.44, 152.46, 68.25, 67.08, 57.95, 52.66, 47.16, 43.25, 40.62, 38.19, 32.57, 31.99, 29.65, 28.79, 25.43. MS (ESI): *m*/*z* = 496.13 [M + H]^+^.

##### (1*S*,3a*R*,6a*S*)-2-(2-(3,4-Dichlorophenoxy)acetyl)-*N*-((*S*)-1-oxo-3-((*S*)-2-oxopyrrolidin-3-yl)propan-2-yl)octahydrocyclopenta[*c*]pyrrole-1-carboxamide (**MI-31**)

**MI-31** was synthesized according to the literature. ^1^H NMR (400 MHz, chloroform-*d*) δ 9.4 (s, 1H), 8.4 (d, *J* = 5.7 Hz, 1H), 7.3–7.2 (m, 1H), 7.0 (d, *J* = 3.0 Hz, 1H), 6.8 (td, *J* = 8.8, 2.9 Hz, 1H), 6.2 (s, 1H), 4.6 (d, *J* = 4.0 Hz, 2H), 4.4 (d, *J* = 2.9 Hz, 1H), 4.3–4.1 (m, 1H), 3.8 (dd, *J* = 10.4, 8.0 Hz, 1H), 3.3–3.1 (m, 3H), 2.8 (dp, *J* = 12.2, 4.3, 3.9 Hz, 1H), 2.7 (tdd, *J* = 8.0, 5.6, 2.9 Hz, 1H), 2.4 (dt, *J* = 16.1, 8.0 Hz, 1H), 2.3 (ddd, *J* = 12.3, 6.5, 2.3 Hz, 1H), 2.0–1.9 (m, 1H), 1.8 (tt, *J* = 13.6, 6.4 Hz, 3H), 1.8–1.6 (m, 2H), 1.5 (ddd, *J* = 22.4, 12.8, 6.0 Hz, 2H), 1.4 (tt, *J* = 12.6, 5.6 Hz, 1H). ^13^C NMR (100 MHz, chloroform-*d*) δ 200.0, 180.3, 172.8, 166.6, 157.1, 132.8, 130.7, 124.8, 116.7, 114.9, 67.1, 67.0, 58.1, 55.0, 52.6, 47.3, 43.2, 40.6, 32.5, 32.0, 29.7, 28.8, 25.4. MS (ESI): *m*/*z* = 496.13 [M + H]^+^.

## Supporting Information Available

Additional figures, tables, experimental procedures, compound characterizations, and NMR spectra (PDF)
Molecular formula strings (CSV)

## Abbreviations

Boc: *t*-butyloxycarbonyl
CBZ: carboxybenzyl
CoV: coronavirus
COVID-19: coronavirus disease 2019
DCM: dichloromethane
DMEM: Dulbecco’s modified Eagle medium
DMF: dimethylformamide
DMSO: dimethylsulfoxide
DTT: dithiothreitol
EDTA: ethylenediaminetetraacetic acid
eGFP: enhanced green fluorescent protein
M^Pro^: main protease
SARS-CoV-2: severe acute respiratory syndrome coronavirus 2
THF: tetrahydrofuran
Tris: tris(hydroxymethyl)aminomethane

## References

[ref1] Lee N.; Hui D.; Wu A.; Chan P.; Cameron P.; Joynt G. M.; Ahuja A.; Yung M. Y.; Leung C. B.; To K. F.; Lui S. F.; Szeto C. C.; Chung S.; Sung J. J. Y. A major outbreak of severe acute respiratory syndrome in Hong Kong. N. Engl. J. Med. 2003, 348, 1986–1994. 10.1056/NEJMoa030685. 12682352

[ref2] Rota P. A.; Oberste M. S.; Monroe S. S.; Nix W. A.; Campagnoli R.; Icenogle J. P.; Peñaranda S.; Bankamp B.; Maher K.; Chen M.-H.; Tong S.; Tamin A.; Lowe L.; Frace M.; DeRisi J. L.; Chen Q.; Wang D.; Erdman D. D.; Peret T. C.; Burns C.; Ksiazek T. G.; Rollin P. E.; Sanchez A.; Liffick S.; Holloway B.; Limor J.; McCaustland K.; Olsen-Rasmussen M.; Fouchier R.; Günther S.; Osterhaus A. D.; Drosten C.; Pallansch M. A.; Anderson L. J.; Bellini W. J. Characterization of a novel coronavirus associated with severe acute respiratory syndrome. Science 2003, 300, 1394–1399. 10.1126/science.1085952. 12730500

[ref3] Memish Z. A.; Mishra N.; Olival K. J.; Fagbo S. F.; Kapoor V.; Epstein J. H.; Alhakeem R.; Durosinloun A.; Al Asmari M.; Islam A.; Kapoor A.; Briese T.; Daszak P.; Al Rabeeah A. A.; Lipkin W. I. Middle East respiratory syndrome coronavirus in bats, Saudi Arabia. Emerg. Infect. Dis. 2013, 19, 1819–1823. 10.3201/eid1911.131172. 24206838 PMC3837665

[ref4] Holshue M. L.; DeBolt C.; Lindquist S.; Lofy K. H.; Wiesman J.; Bruce H.; Spitters C.; Ericson K.; Wilkerson S.; Tural A.; Diaz G.; Cohn A.; Fox L.; Patel A.; Gerber S. I.; Kim L.; Tong S.; Lu X.; Lindstrom S.; Pallansch M. A.; Weldon W. C.; Biggs H. M.; Uyeki T. M.; Pillai S. K. Novel Coronavirus in the United States. N. Engl. J. Med. 2020, 382, 929–936. 10.1056/NEJMoa2001191. 32004427 PMC7092802

[ref5] Huang C.; Wang Y.; Li X.; Ren L.; Zhao J.; Hu Y.; Zhang L.; Fan G.; Xu J.; Gu X.; Cheng Z.; Yu T.; Xia J.; Wei Y.; Wu W.; Xie X.; Yin W.; Li H.; Liu M.; Xiao Y.; Gao H.; Guo L.; Xie J.; Wang G.; Jiang R.; Gao Z.; Jin Q.; Wang J.; Cao B. Clinical features of patients infected with 2019 novel coronavirus in Wuhan, China. Lancet 2020, 395, 497–506. 10.1016/S0140-6736(20)30183-5. 31986264 PMC7159299

[ref6] Beigel J. H.; Tomashek K. M.; Dodd L. E.; Mehta A. K.; Zingman B. S.; Kalil A. C.; Hohmann E.; Chu H. Y.; Luetkemeyer A.; Kline S.; Lopez de Castilla D.; Finberg R. W.; Dierberg K.; Tapson V.; Hsieh L.; Patterson T. F.; Paredes R.; Sweeney D. A.; Short W. R.; Touloumi G.; Lye D. C.; Ohmagari N.; Oh M. D.; Ruiz-Palacios G. M.; Benfield T.; Fatkenheuer G.; Kortepeter M. G.; Atmar R. L.; Creech C. B.; Lundgren J.; Babiker A. G.; Pett S.; Neaton J. D.; Burgess T. H.; Bonnett T.; Green M.; Makowski M.; Osinusi A.; Nayak S.; Lane H. C.; Members A.-S. G. Remdesivir for the Treatment of Covid-19 - Final Report. N. Engl. J. Med. 2020, 383, 1813–1826. 10.1056/NEJMoa2007764. 32445440 PMC7262788

[ref7] Fischer W. A. II; Eron J. J. Jr.; Holman W.; Cohen M. S.; Fang L.; Szewczyk L. J.; Sheahan T. P.; Baric R.; Mollan K. R.; Wolfe C. R.; Duke E. R.; Azizad M. M.; Borroto-Esoda K.; Wohl D. A.; Coombs R. W.; James Loftis A.; Alabanza P.; Lipansky F.; Painter W. P. A phase 2a clinical trial of molnupiravir in patients with COVID-19 shows accelerated SARS-CoV-2 RNA clearance and elimination of infectious virus. Sci. Transl. Med. 2022, 14, eabl7430 10.1126/scitranslmed.abl7430. 34941423 PMC10763622

[ref8] Owen D. R.; Allerton C. M. N.; Anderson A. S.; Aschenbrenner L.; Avery M.; Berritt S.; Boras B.; Cardin R. D.; Carlo A.; Coffman K. J.; Dantonio A.; Di L.; Eng H.; Ferre R.; Gajiwala K. S.; Gibson S. A.; Greasley S. E.; Hurst B. L.; Kadar E. P.; Kalgutkar A. S.; Lee J. C.; Lee J.; Liu W.; Mason S. W.; Noell S.; Novak J. J.; Obach R. S.; Ogilvie K.; Patel N. C.; Pettersson M.; Rai D. K.; Reese M. R.; Sammons M. F.; Sathish J. G.; Singh R. S. P.; Steppan C. M.; Stewart A. E.; Tuttle J. B.; Updyke L.; Verhoest P. R.; Wei L.; Yang Q.; Zhu Y. An oral SARS-CoV-2 M(pro) inhibitor clinical candidate for the treatment of COVID-19. Science 2021, 374, 1586–1593. 10.1126/science.abl4784. 34726479

[ref9] Jayk Bernal A.; Gomes da Silva M. M.; Musungaie D. B.; Kovalchuk E.; Gonzalez A.; Delos Reyes V.; Martin-Quiros A.; Caraco Y.; Williams-Diaz A.; Brown M. L.; Du J.; Pedley A.; Assaid C.; Strizki J.; Grobler J. A.; Shamsuddin H. H.; Tipping R.; Wan H.; Paschke A.; Butterton J. R.; Johnson M. G.; De Anda C. Molnupiravir for Oral Treatment of Covid-19 in Nonhospitalized Patients. N. Engl. J. Med. 2022, 386, 509–520. 10.1056/NEJMoa2116044. 34914868 PMC8693688

[ref10] Rock B. M.; Hengel S. M.; Rock D. A.; Wienkers L. C.; Kunze K. L. Characterization of ritonavir-mediated inactivation of cytochrome P450 3A4. Mol. Pharmacol. 2014, 86, 665–674. 10.1124/mol.114.094862. 25274602

[ref11] Staud F.; Ceckova M.; Micuda S.; Pavek P. Expression and function of p-glycoprotein in normal tissues: effect on pharmacokinetics. Methods Mol. Biol. 2010, 596, 199–222. 10.1007/978-1-60761-416-6_10. 19949926

[ref12] Yang K. S.; Leeuwon S. Z.; Xu S.; Liu W. R. Evolutionary and Structural Insights about Potential SARS-CoV-2 Evasion of Nirmatrelvir. J. Med. Chem. 2022, 65, 8686–8698. 10.1021/acs.jmedchem.2c00404. 35731933 PMC9236210

[ref13] Morse J. S.; Lalonde T.; Xu S.; Liu W. R. Learning from the Past: Possible Urgent Prevention and Treatment Options for Severe Acute Respiratory Infections Caused by 2019-nCoV. ChemBioChem 2020, 21, 730–738. 10.1002/cbic.202000047. 32022370 PMC7162020

[ref14] Fu L.; Ye F.; Feng Y.; Yu F.; Wang Q.; Wu Y.; Zhao C.; Sun H.; Huang B.; Niu P.; Song H.; Shi Y.; Li X.; Tan W.; Qi J.; Gao G. F. Both Boceprevir and GC376 efficaciously inhibit SARS-CoV-2 by targeting its main protease. Nat. Commun. 2020, 11, 4417 10.1038/s41467-020-18233-x. 32887884 PMC7474075

[ref15] Dai W.; Zhang B.; Jiang X. M.; Su H.; Li J.; Zhao Y.; Xie X.; Jin Z.; Peng J.; Liu F.; Li C.; Li Y.; Bai F.; Wang H.; Cheng X.; Cen X.; Hu S.; Yang X.; Wang J.; Liu X.; Xiao G.; Jiang H.; Rao Z.; Zhang L. K.; Xu Y.; Yang H.; Liu H. Structure-based design of antiviral drug candidates targeting the SARS-CoV-2 main protease. Science 2020, 368, 1331–1335. 10.1126/science.abb4489. 32321856 PMC7179937

[ref16] Boras B.; Jones R. M.; Anson B. J.; Arenson D.; Aschenbrenner L.; Bakowski M. A.; Beutler N.; Binder J.; Chen E.; Eng H.; Hammond H.; Hammond J.; Haupt R. E.; Hoffman R.; Kadar E. P.; Kania R.; Kimoto E.; Kirkpatrick M. G.; Lanyon L.; Lendy E. K.; Lillis J. R.; Logue J.; Luthra S. A.; Ma C.; Mason S. W.; McGrath M. E.; Noell S.; Obach R. S.; MN O. B.; O’Connor R.; Ogilvie K.; Owen D.; Pettersson M.; Reese M. R.; Rogers T. F.; Rosales R.; Rossulek M. I.; Sathish J. G.; Shirai N.; Steppan C.; Ticehurst M.; Updyke L. W.; Weston S.; Zhu Y.; White K. M.; Garcia-Sastre A.; Wang J.; Chatterjee A. K.; Mesecar A. D.; Frieman M. B.; Anderson A. S.; Allerton C. Preclinical characterization of an intravenous coronavirus 3CL protease inhibitor for the potential treatment of COVID19. Nat. Commun. 2021, 12, 6055 10.1038/s41467-021-26239-2. 34663813 PMC8523698

[ref17] Dampalla C. S.; Zheng J.; Perera K. D.; Wong L.-Y. R.; Meyerholz D. K.; Nguyen H. N.; Kashipathy M. M.; Battaile K. P.; Lovell S.; Kim Y.; Perlman S.; Groutas W. C.; Chang K.-O. Postinfection treatment with a protease inhibitor increases survival of mice with a fatal SARS-CoV-2 infection. Proc. Natl. Acad. Sci. U. S. A. 2021, 118, e2101555118 10.1073/pnas.2101555118. 34210738 PMC8307543

[ref18] Iketani S.; Forouhar F.; Liu H.; Hong S. J.; Lin F. Y.; Nair M. S.; Zask A.; Huang Y.; Xing L.; Stockwell B. R.; Chavez A.; Ho D. D. Lead compounds for the development of SARS-CoV-2 3CL protease inhibitors. Nat. Commun. 2021, 12, 2016 10.1038/s41467-021-22362-2. 33795671 PMC8016852

[ref19] Ma C.; Sacco M. D.; Hurst B.; Townsend J. A.; Hu Y.; Szeto T.; Zhang X.; Tarbet B.; Marty M. T.; Chen Y.; Wang J. Boceprevir, GC-376, and calpain inhibitors II, XII inhibit SARS-CoV-2 viral replication by targeting the viral main protease. Cell Res. 2020, 30, 678–692. 10.1038/s41422-020-0356-z. 32541865 PMC7294525

[ref20] Yang K. S.; Ma X. R.; Ma Y.; Alugubelli Y. R.; Scott D. A.; Vatansever E. C.; Drelich A. K.; Sankaran B.; Geng Z. Z.; Blankenship L. R.; Ward H. E.; Sheng Y. J.; Hsu J. C.; Kratch K. C.; Zhao B.; Hayatshahi H. S.; Liu J.; Li P.; Fierke C. A.; Tseng C.-T. K.; Xu S.; Liu W. R. A Quick Route to Multiple Highly Potent SARS-CoV-2 Main Protease Inhibitors. ChemMedChem 2021, 16, 942–948. 10.1002/cmdc.202000924. 33283984 PMC7979488

[ref21] Ma Y.; Yang K. S.; Geng Z. Z.; Alugubelli Y. R.; Shaabani N.; Vatansever E. C.; Ma X. R.; Cho C. C.; Khatua K.; Xiao J.; Blankenship L. R.; Yu G.; Sankaran B.; Li P.; Allen R.; Ji H.; Xu S.; Liu W. R. A multi-pronged evaluation of aldehyde-based tripeptidyl main protease inhibitors as SARS-CoV-2 antivirals. Eur. J. Med. Chem. 2022, 240, 114570 10.1016/j.ejmech.2022.114570. 35779291 PMC9235293

[ref22] Alugubelli Y. R.; Geng Z. Z.; Yang K. S.; Shaabani N.; Khatua K.; Ma X. R.; Vatansever E. C.; Cho C. C.; Ma Y.; Xiao J.; Blankenship L. R.; Yu G.; Sankaran B.; Li P.; Allen R.; Ji H.; Xu S.; Liu W. R. A systematic exploration of boceprevir-based main protease inhibitors as SARS-CoV-2 antivirals. Eur. J. Med. Chem. 2022, 240, 114596 10.1016/j.ejmech.2022.114596. 35839690 PMC9264725

[ref23] Hoffman R. L.; Kania R. S.; Brothers M. A.; Davies J. F.; Ferre R. A.; Gajiwala K. S.; He M.; Hogan R. J.; Kozminski K.; Li L. Y.; Lockner J. W.; Lou J.; Marra M. T.; Mitchell L. J. Jr.; Murray B. W.; Nieman J. A.; Noell S.; Planken S. P.; Rowe T.; Ryan K.; Smith G. J. III; Solowiej J. E.; Steppan C. M.; Taggart B. Discovery of Ketone-Based Covalent Inhibitors of Coronavirus 3CL Proteases for the Potential Therapeutic Treatment of COVID-19. J. Med. Chem. 2020, 63, 12725–12747. 10.1021/acs.jmedchem.0c01063. 33054210

[ref24] Jin Z.; Du X.; Xu Y.; Deng Y.; Liu M.; Zhao Y.; Zhang B.; Li X.; Zhang L.; Peng C.; Duan Y.; Yu J.; Wang L.; Yang K.; Liu F.; Jiang R.; Yang X.; You T.; Liu X.; Yang X.; Bai F.; Liu H.; Liu X.; Guddat L. W.; Xu W.; Xiao G.; Qin C.; Shi Z.; Jiang H.; Rao Z.; Yang H. Structure of M^pro^ from SARS-CoV-2 and discovery of its inhibitors. Nature 2020, 582, 289–293. 10.1038/s41586-020-2223-y. 32272481

[ref25] Rathnayake A. D.; Zheng J.; Kim Y.; Perera K. D.; Mackin S.; Meyerholz D. K.; Kashipathy M. M.; Battaile K. P.; Lovell S.; Perlman S.; Groutas W. C.; Chang K. O. 3C-like protease inhibitors block coronavirus replication in vitro and improve survival in MERS-CoV-infected mice. Sci. Transl. Med. 2020, 12, eabc5332 10.1126/scitranslmed.abc5332. 32747425 PMC7574915

[ref26] Zhang L.; Lin D.; Sun X.; Curth U.; Drosten C.; Sauerhering L.; Becker S.; Rox K.; Hilgenfeld R. Crystal structure of SARS-CoV-2 main protease provides a basis for design of improved α-ketoamide inhibitors. Science 2020, 368, 409–412. 10.1126/science.abb3405. 32198291 PMC7164518

[ref27] Bai B.; Arutyunova E.; Khan M. B.; Lu J.; Joyce M. A.; Saffran H. A.; Shields J. A.; Kandadai A. S.; Belovodskiy A.; Hena M.; Vuong W.; Lamer T.; Young H. S.; Vederas J. C.; Tyrrell D. L.; Lemieux M. J.; Nieman J. A. Peptidomimetic nitrile warheads as SARS-CoV-2 3CL protease inhibitors. RSC Med. Chem. 2021, 12, 1722–1730. 10.1039/D1MD00247C. 34778773 PMC8529539

[ref28] Qiao J.; Li Y.-S.; Zeng R.; Liu F.-L.; Luo R.-H.; Huang C.; Wang Y.-F.; Zhang J.; Quan B.; Shen C.; Mao X.; Liu X.; Sun W.; Yang W.; Ni X.; Wang K.; Xu L.; Duan Z.-L.; Zou Q.-C.; Zhang H.-L.; Qu W.; Long Y.-H.-P.; Li M.-H.; Yang R.-C.; Liu X.; You J.; Zhou Y.; Yao R.; Li W.-P.; Liu J.-M.; Chen P.; Liu Y.; Lin G.-F.; Yang X.; Zou J.; Li L.; Hu Y.; Lu G.-W.; Li W.-M.; Wei Y.-Q.; Zheng Y. T.; Lei J.; Yang S. SARS-CoV-2 M^pro^ inhibitors with antiviral activity in a transgenic mouse model. Science 2021, 371, 1374–1378. 10.1126/science.abf1611. 33602867 PMC8099175

[ref29] Vuong W.; Fischer C.; Khan M. B.; van Belkum M. J.; Lamer T.; Willoughby K. D.; Lu J.; Arutyunova E.; Joyce M. A.; Saffran H. A.; Shields J. A.; Young H. S.; Nieman J. A.; Tyrrell D. L.; Lemieux M. J.; Vederas J. C. Improved SARS-CoV-2 M(pro) inhibitors based on feline antiviral drug GC376: Structural enhancements, increased solubility, and micellar studies. Eur. J. Med. Chem. 2021, 222, 113584 10.1016/j.ejmech.2021.113584. 34118724 PMC8164773

[ref30] Bai B.; Belovodskiy A.; Hena M.; Kandadai A. S.; Joyce M. A.; Saffran H. A.; Shields J. A.; Khan M. B.; Arutyunova E.; Lu J.; Bajwa S. K.; Hockman D.; Fischer C.; Lamer T.; Vuong W.; van Belkum M. J.; Gu Z.; Lin F.; Du Y.; Xu J.; Rahim M.; Young H. S.; Vederas J. C.; Tyrrell D. L.; Lemieux M. J.; Nieman J. A. Peptidomimetic α-Acyloxymethylketone Warheads with Six-Membered Lactam P1 Glutamine Mimic: SARS-CoV-2 3CL Protease Inhibition, Coronavirus Antiviral Activity, and in *Vitro* Biological Stability. J. Med. Chem. 2022, 65, 2905–2925. 10.1021/acs.jmedchem.1c00616. 34242027

[ref31] Dai W.; Jochmans D.; Xie H.; Yang H.; Li J.; Su H.; Chang D.; Wang J.; Peng J.; Zhu L.; Nian Y.; Hilgenfeld R.; Jiang H.; Chen K.; Zhang L.; Xu Y.; Neyts J.; Liu H. Design, Synthesis, and Biological Evaluation of Peptidomimetic Aldehydes as Broad-Spectrum Inhibitors against Enterovirus and SARS-CoV-2. J. Med. Chem. 2022, 65, 2794–2808. 10.1021/acs.jmedchem.0c02258. 33872498

[ref32] Konno S.; Kobayashi K.; Senda M.; Funai Y.; Seki Y.; Tamai I.; Schäkel L.; Sakata K.; Pillaiyar T.; Taguchi A.; Taniguchi A.; Gütschow M.; Müller C. E.; Takeuchi K.; Hirohama M.; Kawaguchi A.; Kojima M.; Senda T.; Shirasaka Y.; Kamitani W.; Hayashi Y. 3CL Protease Inhibitors with an Electrophilic Arylketone Moiety as Anti-SARS-CoV-2 Agents. J. Med. Chem. 2022, 65, 2926–2939. 10.1021/acs.jmedchem.1c00665. 34313428

[ref33] Yang K. S.; Blankenship L. R.; Kuo S.-T. A.; Sheng Y. J.; Li P.; Fierke C. A.; Russell D. H.; Yan X.; Xu S.; Liu W. R. A Novel Y-Shaped, S-O-N-O-S-Bridged Cross-Link between Three Residues C22, C44, and K61 Is Frequently Observed in the SARS-CoV-2 Main Protease. ACS Chem. Biol. 2023, 18, 449–455. 10.1021/acschembio.2c00695. 36629751 PMC10023456

[ref34] Rabe von Pappenheim F.; Wensien M.; Ye J.; Uranga J.; Irisarri I.; de Vries J.; Funk L. M.; Mata R. A.; Tittmann K. Widespread occurrence of covalent lysine-cysteine redox switches in proteins. Nat. Chem. Biol. 2022, 18, 368–375. 10.1038/s41589-021-00966-5. 35165445 PMC8964421

[ref35] Cao W.; Cho C.-C. D.; Geng Z. Z.; Shaabani N.; Ma X. R.; Vatansever E. C.; Alugubelli Y. R.; Ma Y.; Chaki S. P.; Ellenburg W. H.; Yang K. S.; Qiao Y.; Allen R.; Neuman B. W.; Ji H.; Xu S.; Liu W. R. Evaluation of SARS-CoV-2 Main Protease Inhibitors Using a Novel Cell-Based Assay. ACS Cent. Sci. 2022, 8, 192–204. 10.1021/acscentsci.1c00910. 35229034 PMC8848508

[ref36] Vatansever E. C.; Yang K. S.; Drelich A. K.; Kratch K. C.; Cho C. C.; Kempaiah K. R.; Hsu J. C.; Mellott D. M.; Xu S.; Tseng C. K.; Liu W. R. Bepridil is potent against SARS-CoV-2 in vitro. Proc. Natl. Acad. Sci. U. S. A. 2021, 118, e2012201118 10.1073/pnas.2012201118. 33597253 PMC7958448

[ref37] Zhang C. H.; Stone E. A.; Deshmukh M.; Ippolito J. A.; Ghahremanpour M. M.; Tirado-Rives J.; Spasov K. A.; Zhang S.; Takeo Y.; Kudalkar S. N.; Liang Z.; Isaacs F.; Lindenbach B.; Miller S. J.; Anderson K. S.; Jorgensen W. L. Potent Noncovalent Inhibitors of the Main Protease of SARS-CoV-2 from Molecular Sculpting of the Drug Perampanel Guided by Free Energy Perturbation Calculations. ACS Cent. Sci. 2021, 7, 467–475. 10.1021/acscentsci.1c00039. 33786375 PMC7931627

[ref38] Ma C.; Xia Z.; Sacco M. D.; Hu Y.; Townsend J. A.; Meng X.; Choza J.; Tan H.; Jang J.; Gongora M. V.; Zhang X.; Zhang F.; Xiang Y.; Marty M. T.; Chen Y.; Wang J. Discovery of Di- and Trihaloacetamides as Covalent SARS-CoV-2 Main Protease Inhibitors with High Target Specificity. J. Am. Chem. Soc. 2021, 143, 20697–20709. 10.1021/jacs.1c08060. 34860011 PMC8672434

[ref39] Hoffmann M.; Kleine-Weber H.; Schroeder S.; Krüger N.; Herrler T.; Erichsen S.; Schiergens T. S.; Herrler G.; Wu N.-H.; Nitsche A.; Müller M. A.; Drosten C.; Pöhlmann S. SARS-CoV-2 Cell Entry Depends on ACE2 and TMPRSS2 and Is Blocked by a Clinically Proven Protease Inhibitor. Cell 2020, 181, 271–280.e8. 10.1016/j.cell.2020.02.052. 32142651 PMC7102627

[ref40] Zhao M. M.; Yang W. L.; Yang F. Y.; Zhang L.; Huang W. J.; Hou W.; Fan C. F.; Jin R. H.; Feng Y. M.; Wang Y. C.; Yang J. K. Cathepsin L plays a key role in SARS-CoV-2 infection in humans and humanized mice and is a promising target for new drug development. Signal Transduction Targeted Ther. 2021, 6, 134 10.1038/s41392-021-00558-8. PMC7997800 33774649

[ref41] Cheng Y. W.; Chao T. L.; Li C. L.; Chiu M. F.; Kao H. C.; Wang S. H.; Pang Y. H.; Lin C. H.; Tsai Y. M.; Lee W. H.; Tao M. H.; Ho T. C.; Wu P. Y.; Jang L. T.; Chen P. J.; Chang S. Y.; Yeh S. H. Furin Inhibitors Block SARS-CoV-2 Spike Protein Cleavage to Suppress Virus Production and Cytopathic Effects. Cell Rep. 2020, 33, 108254 10.1016/j.celrep.2020.108254. 33007239 PMC7510585

[ref42] Kumar P.; Nagarajan A.; Uchil P. D. Analysis of Cell Viability by the MTT Assay. Cold Spring Harbor Protoc. 2018, 2018, 469–471. 10.1101/pdb.prot095505 29858338

