# Supplementary material for: A Systematic Survey of Reversibly Covalent Dipeptidyl Inhibitors of the SARS-CoV-2 Main Protease
Source: J Med Chem. 2023 Aug 10;66(16):11040–55. doi: 10.1021/acs.jmedchem.3c00221 (PMC10861299; doi:10.1021/acs.jmedchem.3c00221)

Supporting Information

for

A Systematic Survey of Reversibly Covalent Dipeptidyl Inhibitors of the SARS-CoV-2 Main Protease

Zhi Zachary Geng[a,†], Sandeep Atla[a,†], Namir Shaabani[b,†], Veerabhadra Vulupala[a,†], Kai S. Yang[a,†], Yugendar R. Alugubelli[a,†], Kaustav Khatua[a,†], Peng-Hsun Chen[a], Jing Xiao[a], Lauren R. Blankenship[a], Xinyu R. Ma[a], Erol C. Vatansever[a], Chia-Chuan D. Cho[a], Yuying Ma[a], Robert Allen[b], Henry Ji[b,*], Shiqing Xu[a,c,*], and Wenshe Ray Liu[a,d,e,f,*]

[a]Texas A&M Drug Discovery Laboratory, Department of Chemistry, Texas A&M University, College Station, TX 77843, USA

[b]Sorrento Therapeutics, Inc. San Diego, CA 92121, USA

[c]Department of Pharmaceutical Sciences, Irma Lerma Rangel College of Pharmacy, Texas A&M University, College Station, TX 77843, USA

[d]Department of Biochemistry and Biophysics, Texas A&M University, College Station, TX 77843, USA

[e]Institute of Biosciences and Technology and Department of Translational Medical Sciences, College of Medicine, Texas A&M University, Houston, TX 77030, USA

[f]Department of Molecular and Cellular Medicine, College of Medicine, Texas A&M University, College Station, TX 77843, USA

[†]Contributed equally to the paper.

*Correspondence should be addressed to Henry Ji: hji@sorrentotherapeutics.com, Shiqing Xu: shiqing.xu@tamu.edu and Wenshe Ray Liu: wsliu2007@tamu.edu

Table of Contents

Supplementary Figures..........3

Table S1. Data Collection and refinement statistics..........10

Supplementary Synthesis and Characterization of Compounds..........11

Additional Supplementary NMR Spectra of Finally Synthesized Compounds..........26

LC-MS..........54

Supplementary Figures

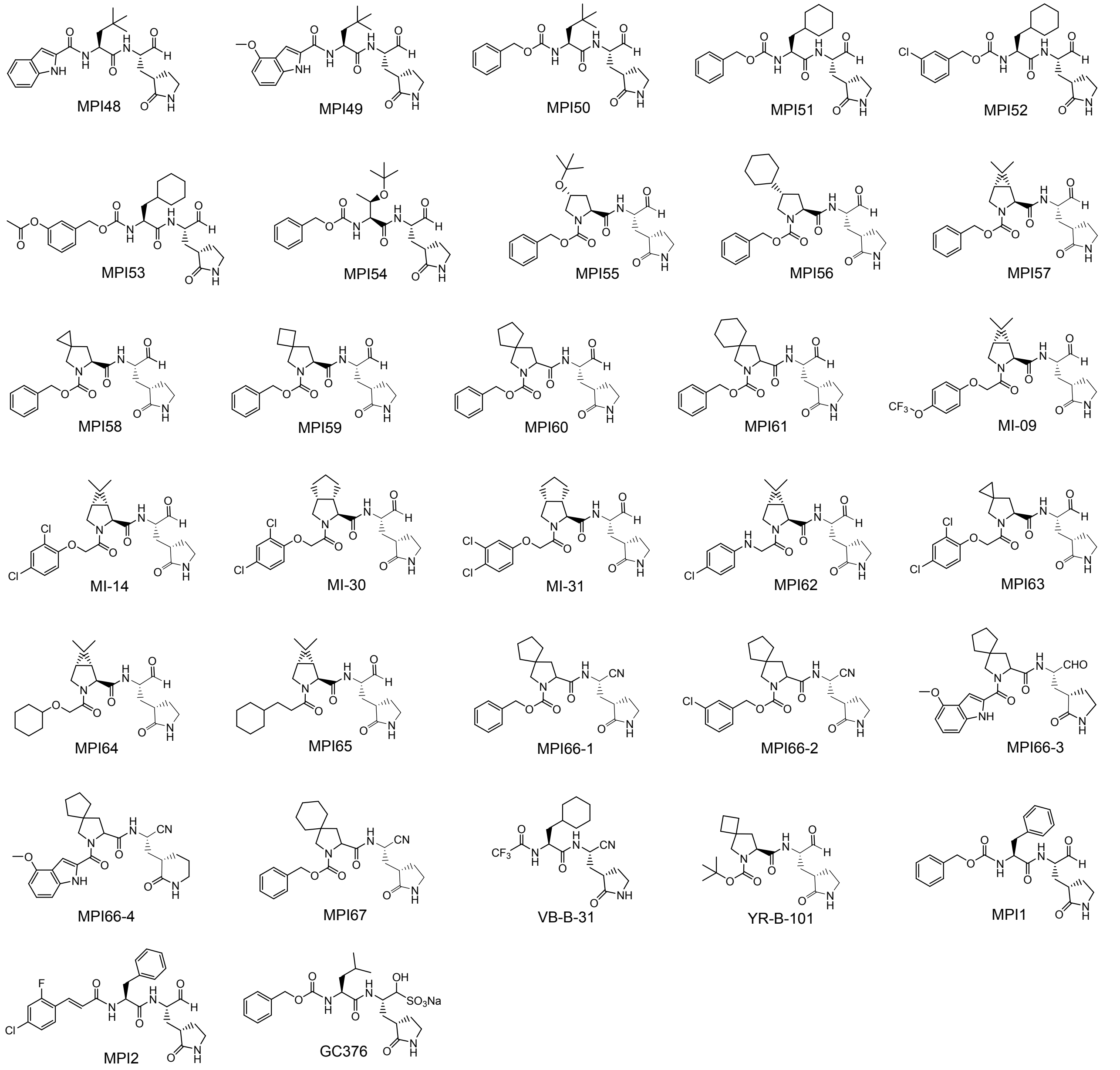

Figure S1. Structures of dipeptidyl M^{Pro} inhibitors.

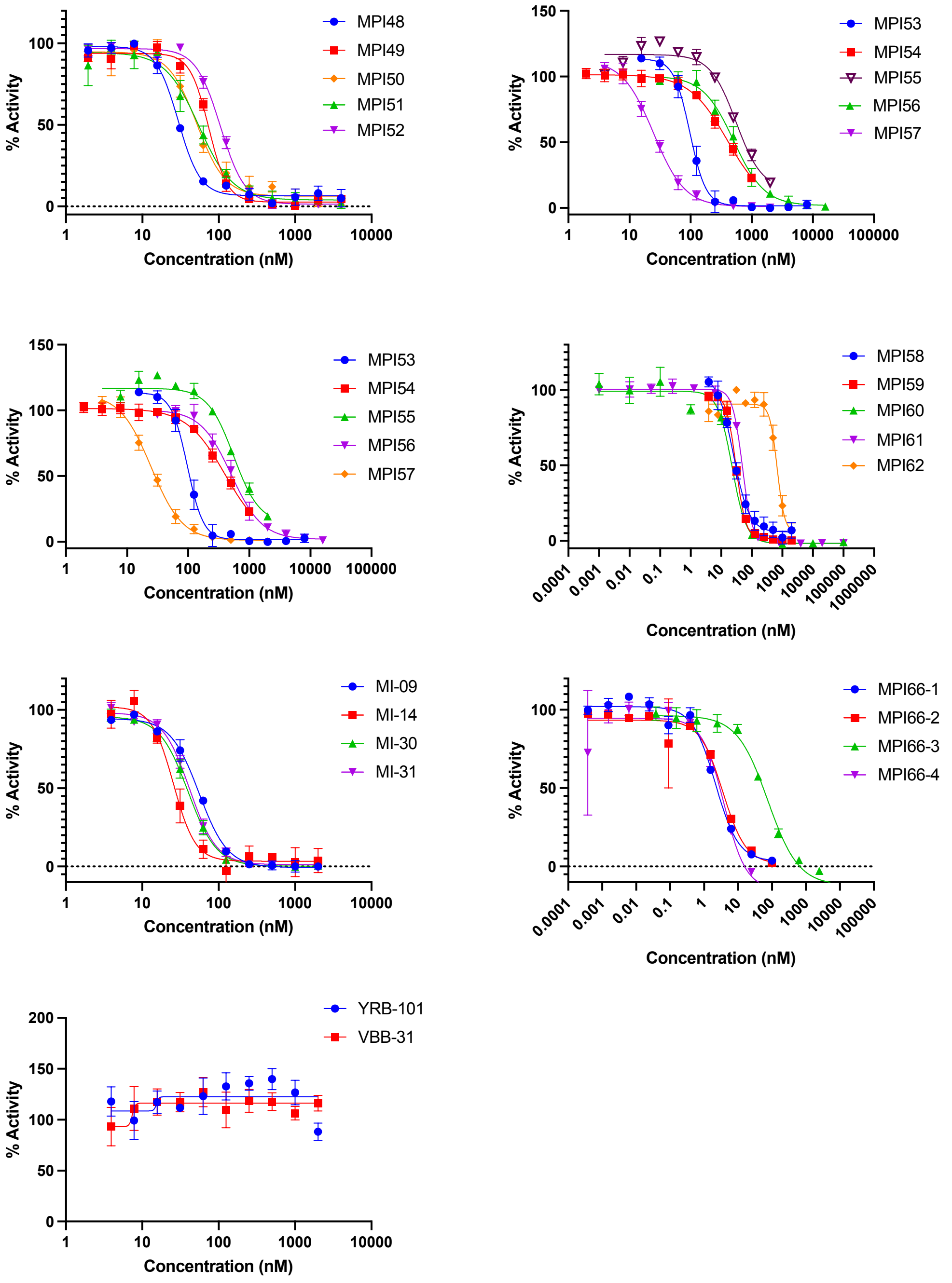

Figure S2. Inhibition curves of compounds on M^{Pro}. Triplicate experiments were performed for each compound. For all experiments, 20 or 10 nM M^{Pro} was incubated with an inhibitor for 30 min before 10 μM Sub3 was added. The M^{Pro}-catalyzed Sub3 hydrolysis rate was determined by measuring linear increase of product fluorescence (Ex: 336 nm/Em: 455 nm) for 5 min.

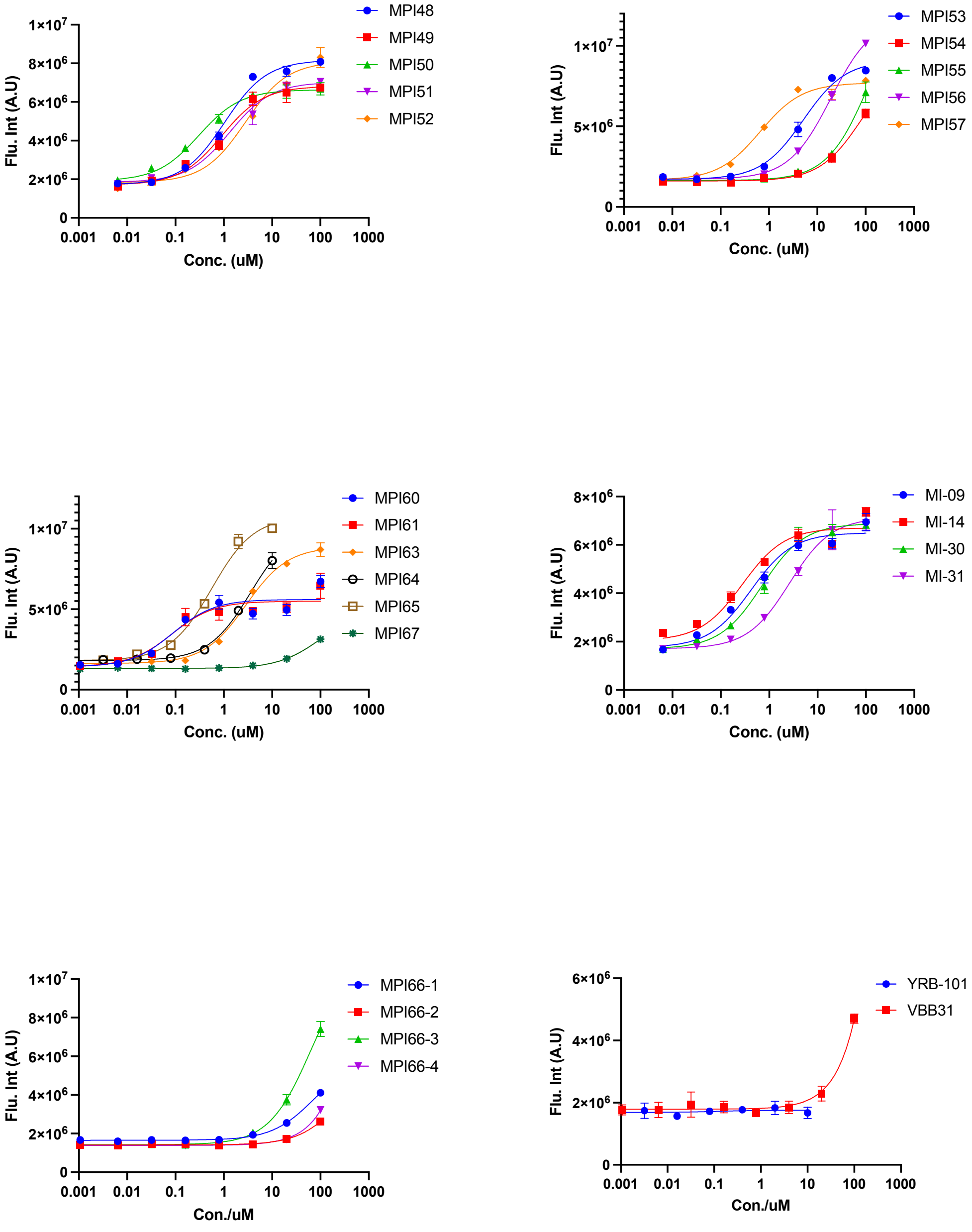

Figure S3. Cellular potency of inhibitors in their inhibition of M^{Pro} to drive host 293T cell survival and overall M^{Pro}-eGFP expression.

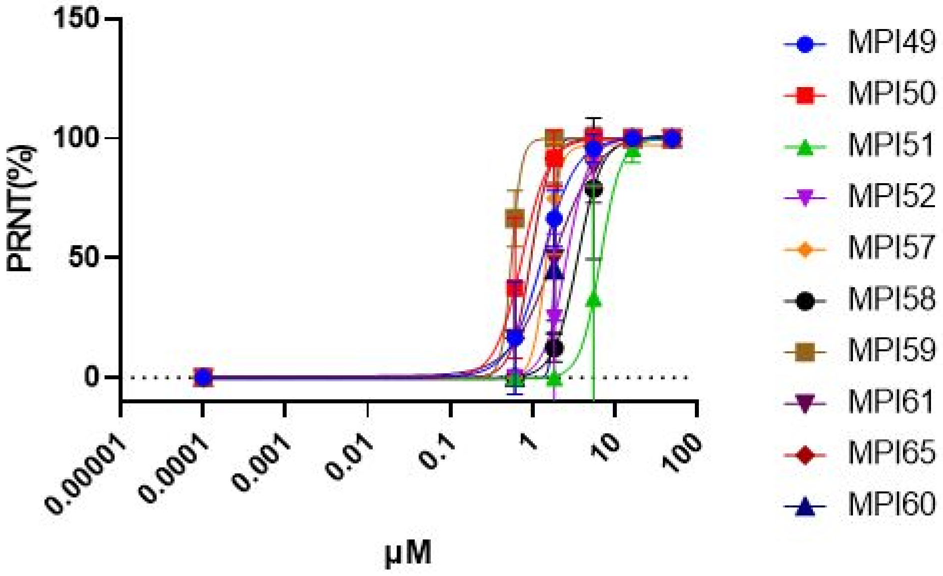

Figure S4. Plaque reduction neutralization tests (PRNTs) of selected compounds on their inhibition of three SARS-CoV-2 in Vero E6 cells. Two repeats were conducted for each concentration.

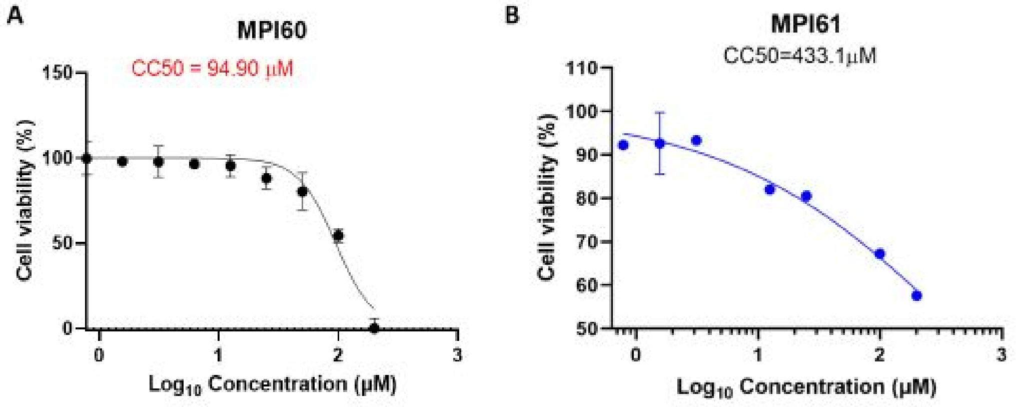

Figure S5. Cytotoxicity curves of MPI60 and MPI61 in 293T cells tested by the MTT assay.

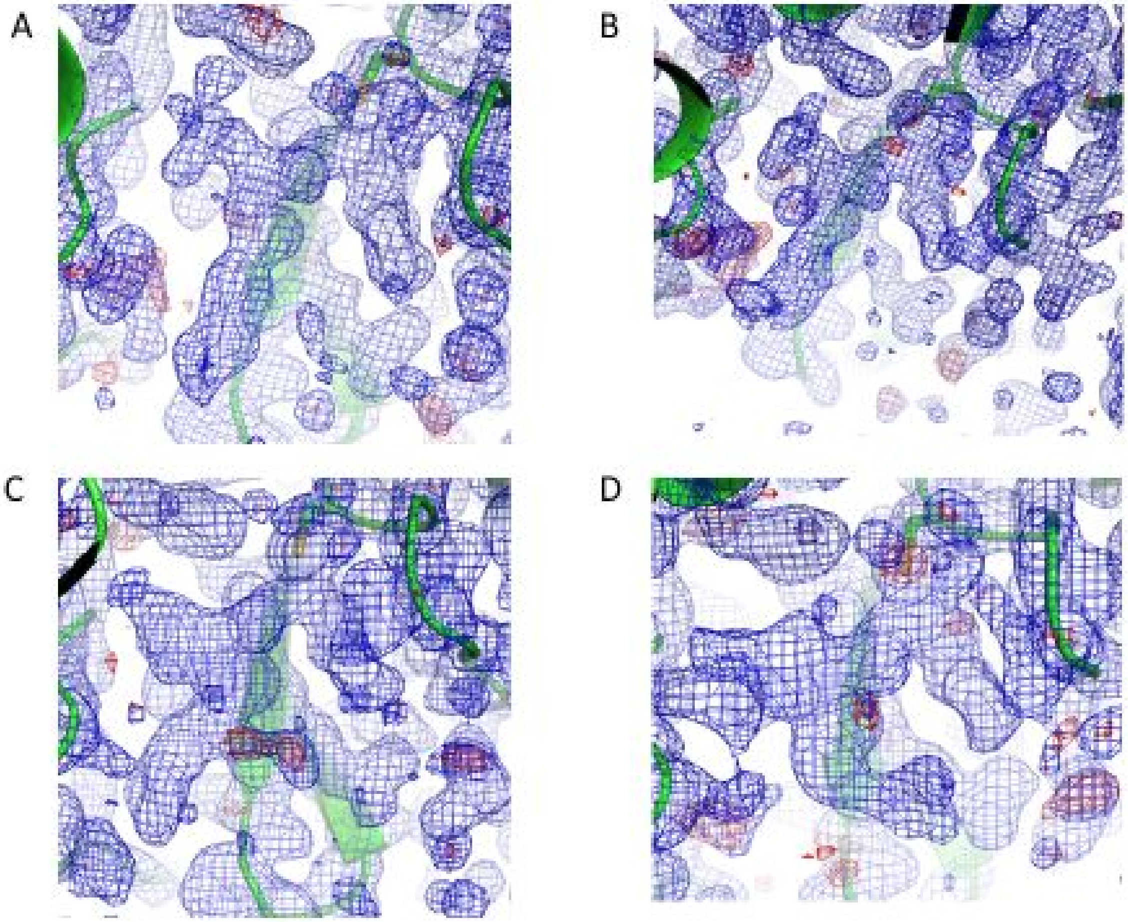

Figure S6: The crystal structures of (**A**) M^{Pro}-MPI48 (PDB ID: 7SD9), (**B**) M^{Pro}-MPI49 (PDB ID: 7SDA), and (**C**) M^{Pro}-MI-09 (PDB ID: 7SDC). (**D**) M^{Pro}-MPI-60 (PDB ID: 8STY). The fo-fc maps around the inhibitor and C145 in all three structures were contoured at 3σ.

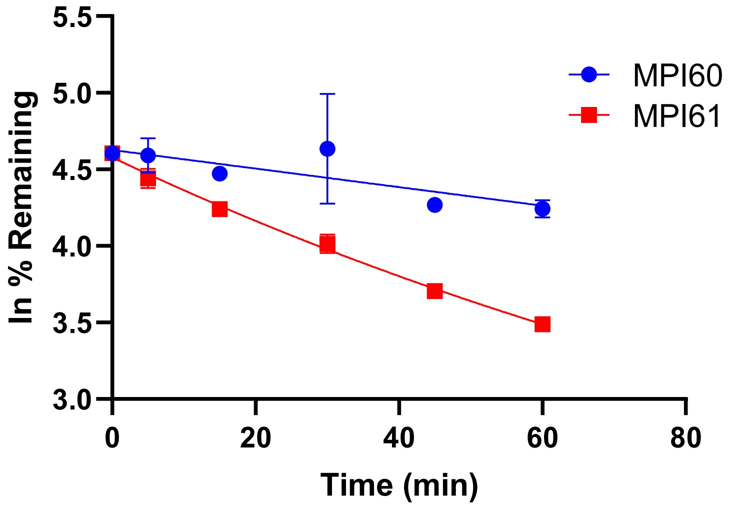

Figure S7. The metabolic degradation of MPI60 and MPI61 (5 μM) in human liver microsomes in the presence of 5 mM NADPH.

Table S1. Data Collection and refinement statistics.

Ligand (PDB Entry)	**MPI48 (7SD9)**	**MPI49 (7SDA)**	**MI-09 (7SDC)**	**MPI60 (8STY)**
Data Collection				
Space Group	I 1 2 1	I 1 2 1	I 1 2 1	I 1 2 1
cell dimensions				
a, b, c (Å)	51.661 80.8574 89.6971	51.6838 81.3006 89.2993	54.3646 80.9874 87.8118	54.3565 80.6287 86.2524
α, β, γ (°)	90 96.6634 90	90 97.0934 90	90 97.2942 90	90 97.1562 90
Resolution Range (Å)	24.28 - 1.85 (1.916-1.85)	24.32 - 1.85 (1.91 - 1.85)	24.35 - 1.85 (1.916 - 1.85)	24.22 - 1.9 (1.968 - 1.90)
Total Reflections	90996	81146	63804	227206
Unique Reflections	29850 (2976)	31237 (3071)	31726 (3108)	29124 (2915)
Multiplicity	4.7	6.6	6.9	7.8
Completeness (%)	95.31 (95.05)	99.37 (97.93)	98.23 (96.40)	98.88 (96.85)
Mean I/sigma(I)	9.5	10.4	9.5	12.88
Wilson B-factor	10.94	17.17	20.72	25.95
R-merge	0.094	0.101	0.121	0.083
R-meas	0.113	0.119	0.139	0.087
R-pim	0.062	0.061	0.068	0.029
CC1/2	0.996	0.997	0.996	0.999
Refinement				
No. Reflections Refinement	29849 (2975)	31128 (3069)	31696 (3104)	28831 (2828)
No. Reflections *R*-free	1508 (161)	1561 (165)	1542 (151)	1480 (151)
R-work	0.2192 (0.3465)	0.2124 (0.3151)	0.2600 (0.4761)	0.2144 (0.3138)
R-free	0.2483 (0.3864)	0.2378 (0.3362)	0.3006 (0.5238)	0.2492 (0.3378)
No. atoms				
Non-Hydrogen	2610	2629	2566	2602
Macromolecules	2360	2360	2360	2360
Ligands	31	33	36	32
Water	219	236	170	203
Protein Residues	306	306	306	306
RMS				
Bond Length (Å)	0.010	0.010	0.016	0.009
Bond Angles (°)	1.21	1.22	1.59	1.09

Supplementary Synthesis and Characterization of Compounds

Methyl (S)-2-(1H-Indole-2-carboxamido)-4,4-dimethylpentanoate (MPI48c). MPI48c was prepared with methyl (S)-2-amino-4,4-dimethylpentanoate hydrochloride (MPI48b) and 1H-indole-2-carboxylic acid (MPI48a) as a white solid following a general procedure **A** (yield 70%).

(S)-2-(1H-Indole-2-carboxamido)-4,4-dimethylpentanoic acid (MPI48d). MPI48d was prepared as a white solid following a general procedure **B**.

Methyl (S)-2-((S)-2-(1H-indole-2-carboxamido)-4,4-dimethylpentanamido)-3-((S)-2-oxopyrrolidin-3-yl)propanoate (MPI48e). MPI48e was prepared with Int.i and 1H-indole-2-carboxylic acid (MPI48d) as a white solid following a general procedure **C** (yield 60%). ^{1}H NMR (400 MHz, DMSO-d6) δ 11.57 (s, 1H), 8.48 (dd, J = 21.5, 8.1 Hz, 2H), 7.70 – 7.58 (m, 2H), 7.42 (d, J = 8.2 Hz, 1H), 7.23 (s, 1H), 7.17 (t, J = 7.6 Hz, 1H), 7.03 (t, J = 7.4 Hz, 1H), 4.68 – 4.55 (m, 1H), 4.38 – 4.28 (m, 1H), 3.61 (s, 3H), 3.17 – 3.02 (m, 2H), 2.38 – 2.27 (m, 1H), 2.16 – 2.02 (m, 2H), 1.82 – 1.52 (m, 4H), 0.94 (s, 9H).

N-((S)-1-(((S)-1-Hydroxy-3-((S)-2-oxopyrrolidin-3-yl)propan-2-yl)amino)-4,4-dimethyl-1-oxopentan-2-yl)-1H-indole-2-carboxamide (MPI48f). **MPI48f** was prepared as a white solid following a general procedure **D** (yield 60%). ^{1}H NMR (400 MHz, DMSO-d6) δ 11.57 (s, 1H), 8.48 (dd, J = 21.5, 8.1 Hz, 2H), 7.70 – 7.58 (m, 2H), 7.42 (d, J = 8.2 Hz, 1H), 7.23 (s, 1H), 7.17 (t, J = 7.6 Hz, 1H), 7.03 (t, J = 7.4 Hz, 1H), 4.68 – 4.55 (m, 1H), 4.38 – 4.28 (m, 1H), 3.61 (s, 3H), 3.17 – 3.02 (m, 2H), 2.38 – 2.27 (m, 1H), 2.16 – 2.02 (m, 2H), 1.82 – 1.52 (m, 4H), 0.94 (s, 9H).

(S)-Methyl 2-(4-methoxy-1H-indole-2-carboxamido)-4,4-dimethylpentanoate (MPI49c). MPI49c was prepared with methyl (S)-2-amino-4,4-dimethylpentanoate hydrochloride (MPI48b) and 4-methoxy-1H-indole-2-carboxylic acid (MPI49a) as a white solid following general procedure **A** (yield 79%). ^{1}H NMR (400 MHz, Chloroform-d) δ 10.02 (s, 1H), 7.18 (t, J = 8.0 Hz, 1H), 7.09 – 7.03 (m, 2H), 6.77 (d, J = 8.6 Hz, 1H), 6.48 (d, J = 7.7 Hz, 1H), 4.94 (td, J = 8.9, 3.6 Hz, 1H), 3.93 (s, 3H), 3.76 (s, 3H), 1.89 (dd, J = 14.4, 3.6 Hz, 1H), 1.68 (dd, J = 14.4, 9.1 Hz, 1H), 1.02 (s, 9H). ^{13}C NMR (101 MHz, CDCl3): δ 173.99, 161.39, 154.13, 138.11, 128.91, 125.47, 118.84, 105.32, 100.55, 99.53, 60.44, 55.26, 52.51, 50.14, 30.80, 29.67.

(S)-2-(4-Methoxy-1H-indole-2-carboxamido)-4,4-dimethylpentanoic acid (MPI49d). MPI49d was prepared as a white solid following a general procedure **B** (290 mg, 86%). ^{1}H NMR (400 MHz, DMSO-d6) δ 12.60 (s, 1H), 11.58 (s, 1H), 8.53 (d, J = 8.3 Hz, 1H), 7.33 (d, J = 5.2 Hz, 1H), 7.17 – 6.93 (m, 2H), 6.51 (t, J = 6.2 Hz, 1H), 4.52 (t, J = 8.4 Hz, 1H), 3.89 (s, 3H), 1.93 – 1.80 (m, 1H), 1.78 – 1.70 (m, 1H), 0.95 (s, 9H). 13C NMR (101 MHz, DMSO). δ 175.06, 161.16, 154.08, 138.29, 130.48, 124.89, 118.54, 105.88, 101.34, 99.65, 60.23, 55.50, 49.95, 44.16, 30.89, 29.84.

(S)-Methyl 2-((S)-2-(4-methoxy-1H-indole-2-carboxamido)-4,4-dimethylpentanamido)-3-((S)-2-oxopyrrolidin-3-yl)propanoate (MPI49e). MPI49e was prepared with Int.i and MPI49d as a white gummy solid following general procedure **C** (yield 72%). ^{1}H NMR (400 MHz, Methanol-d4) δ 8.47 (dd, J = 28.2, 7.9 Hz, 1H), 8.22 (dd, J = 17.4, 8.1 Hz, 1H), 7.14 (d, J = 2.8 Hz, 1H), 7.03 (td, J = 8.0, 2.7 Hz, 1H), 6.92 (dd, J = 8.3, 4.9 Hz, 1H), 6.39 (dd, J = 7.8, 2.7 Hz, 1H), 4.68 – 4.59 (m, 1H), 4.48 – 4.35 (m, 1H), 3.81 (s, 3H), 3.60 (s, 2H), 3.16 – 3.02 (m, 2H), 2.51 – 2.39 (m, 1H), 2.26 – 2.02 (m, 2H), 1.86 – 1.55 (m, 4H), 0.92 (s, 9H). ^{13}C NMR (101 MHz, MeOD): δ 180.37, 174.37, 172.27, 162.14, 154.24, 138.41, 129.05, 124.97, 118.71, 104.89, 104.81, 101.53, 98.93, 54.32, 51.47, 51.15, 50.69, 44.44, 40.03, 38.18, 30.13, 30.07, 28.81, 27.29.

N-((S)-1-(((S)-1-Hydroxy-3-((S)-2-oxopyrrolidin-3-yl)propan-2-yl)amino)-4,4-dimethyl-1-oxopentan-2-yl)-4-methoxy-1H-indole-2-carboxamide (MPI49f). MPI49f was prepared as a white solid following a general procedure **D** (yield 58%). ^{1}H NMR (400 MHz, Chloroform-d) δ 10.39 (s, 1H), 8.06 (d, J = 8.0 Hz, 1H), 7.20 – 7.12 (m, 1H), 7.10 – 6.98 (m, 2H), 6.91 (s, 1H), 6.55 – 6.37 (m, 1H), 5.92 (s, 1H), 4.81 – 4.63 (m, 1H), 4.10 – 3.98 (m, 1H), 3.93 (s, 3H), 3.72 – 3.58 (m, 2H), 3.24 – 2.90 (m, 2H), 2.45 – 2.33 (m, 1H), 2.28 – 2.17 (m, 1H), 2.13 – 1.92 (m, 3H), 1.63 (dd, J = 13.8, 8.8 Hz, 2H), 1.03 (s, 3H), 0.96 (s, 6H).

(S)-2-(((Benzyloxy)carbonyl)amino)-4,4-dimethylpentanoic acid (MPI50d). MPI50d was prepared as a white solid following a general procedure **G** (yield 84%). ^{1}H NMR (400 MHz, Chloroform-d) δ 7.15 (s, 5H), 5.71 (s, 1H), 5.08 (d, J = 12.5 Hz, 1H), 4.75 (d, J = 12.6 Hz, 1H), 4.10 (s, 1H), 1.67 (d, J = 14.3 Hz, 1H), 1.29 (dd, J = 14.4, 9.1 Hz, 1H), 0.79 (s, 9H). ^{13}C NMR (100 MHz, Chloroform-d) δ 156.70, 136.50, 128.40, 127.89, 66.77, 30.53, 29.68.

Methyl (S)-2-((S)-2-(((benzyloxy)carbonyl)amino)-4,4-dimethylpentanamido)-3-((S)-2-oxopyrrolidin-3-yl)propanoate (MPI50e). MPI50e was prepared with Int.i and MPI50d as a white gummy solid following general procedure **C** (yield 67%). ^{1}H NMR (400 MHz, Chloroform-d) δ 7.74 (d, J = 7.8 Hz, 1H), 7.32 – 7.22 (m, 5H), 6.78 (s, 1H), 5.46 (d, J = 9.2 Hz, 1H), 5.01 (s, 2H), 4.64 – 4.52 (m, 1H), 4.52 – 4.40 (m, 1H), 3.94 – 3.85 (m, 1H), 3.62 (s, 3H), 3.30 – 3.15 (m, 2H), 2.29 (dd, J = 7.1, 3.2 Hz, 2H), 2.23 – 2.10 (m, 1H), 2.10 – 2.00 (m, 1H), 1.80 – 1.68 (m, 3H), 0.85 (s, 9H). ^{13}C NMR (100 MHz, Chloroform-d) δ 179.83, 173.02, 172.00, 170.90, 156.52, 136.22, 128.54, 128.18, 128.00, 67.07, 60.41, 52.33, 50.90, 46.48, 40.54, 30.95, 30.56, 29.55, 27.95, 21.06, 19.20, 18.08, 14.20.

Benzyl ((S)-1-(((S)-1-hydroxy-3-((S)-2-oxopyrrolidin-3-yl)propan-2-yl)amino)-4,4-dimethyl-1-oxopentan-2-yl)carbamate (MPI50f). MPI50f was prepared as a white solid following a general procedure **D** (yield 51%). ^{1}H NMR (400 MHz, Chloroform-d) δ 7.41 – 7.22 (m, 5H), 6.50 (d, J = 6.3 Hz,

1H), 5.93 (s, 1H), 5.41 (s, 1H), 5.07 (d, J = 4.1 Hz, 2H), 4.17 (d, J = 2.9 Hz, 1H), 3.97 (s, 1H), 3.60 (dd, J = 11.8, 3.4 Hz, 1H), 3.50 (dd, J = 11.7, 6.7 Hz, 1H), 3.26 – 3.05 (m, 2H), 2.40 – 2.29 (m, 1H), 2.29 – 2.19 (m, 1H), 2.08 (d, J = 15.7 Hz, 1H), 1.97 – 1.77 (m, 2H), 1.77 – 1.61 (m, 1H), 0.86 (s, 9H). 13C NMR (100 MHz, Chloroform-d) δ 180.44, 173.62, 173.42, 156.25, 135.91, 128.67, 128.46, 128.37, 67.62, 65.70, 63.37, 53.04, 50.63, 45.79, 40.33, 38.10, 35.36, 32.36, 30.69, 29.69, 28.37, 17.45, 17.11, 17.00.

Methyl (S)-2-((S)-2-(((benzyloxy)carbonyl)amino)-3-cyclohexylpropanamido)-3-((S)-2-oxopyrrolidin-3-yl)propanoate (MPI51e). MPI51e was prepared with Int.i and (S)-2-(((benzyloxy)carbonyl)amino)-3-cyclohexylpropanoic acid (MPI51d) as a white gummy solid following general procedure **C** (yield 67%). ^{1}H NMR (400 MHz, Chloroform-d) δ 7.69 (d, J = 7.0 Hz, 1H), 7.42 – 7.29 (m, 5H), 5.92 (s, 1H), 5.29 (d, J = 8.7 Hz, 1H), 5.18 – 5.04 (m, 2H), 4.50 (s, 1H), 4.29 (d, J = 6.3 Hz, 1H), 3.73 (s, 3H), 3.39 – 3.25 (m, 2H), 2.42 (s, 2H), 2.19 – 2.10 (m, 1H), 1.97 – 1.57 (m, 4H), 1.57 – 1.33 (m, 3H), 1.32 – 1.05 (m, 4H), 1.05 – 0.76 (m, 4H).

(S)-Methyl 2-((((3-chlorobenzyl)oxy)carbonyl)amino)-3-cyclohexylpropanoate (MPI52c).

To 3,5-dichlorobenzyl alcohol (0.201 g, 1.39 mmol) in THF (5 mL) were added K_2CO_3 (193 mg, 1.39 mmol) and Triphosgene (166 mg, 0.56 mmol) and the mixture was stirred at rt for 1 h. The mixture was then poured into water (10 mL) and extracted with ethyl acetate (2×20 mL), Combine organic layers and dried over Na_2SO_4. The organic phase was evaporated to dryness and the crude material was used directly in the next step. 3,5-Dichlorobenzyl Chloroformate in THF (5 mL) was added to drop wise to a mixture of methyl (S)-2-amino-3-cyclohexylpropanoate (320 mg,1.39mmol) and DIPEA (0.3 ml, 2.78mmol).The reaction mixture stirred for 12 h. The mixture was then poured into water (30 mL) and extracted with ethyl acetate (4×20 mL). The organic layer was washed with aqueous hydrochloric acid 10% v/v (2×20 mL), saturated aqueous $NaHCO_3$ (2×20 mL), brine (2×20 mL) and dried over Na_2SO_4. The organic phase was evaporated to dryness and the crude material purified by silica gel column chromatography (15-50% EtOAc in n-hexane as the eluent) to afford **MPI52c** white solid (280 mg, 59%). ^{1}H NMR (400 MHz, Chloroform-d) δ 7.45 – 7.05 (m, 4H), 5.22 – 4.86 (m, 2H), 4.34 (td, J = 9.0, 5.1 Hz, 1H), 3.66 (s, 3H), 1.79 – 1.67 (m, 1H), 1.67 – 1.49 (m, 5H), 1.49 – 1.37 (m, 1H), 1.33 – 1.24 (m, 1H), 1.22 – 1.01 (m, 3H), 0.94 – 0.74 (m, 2H).

(S)-2-((((3-Chlorobenzyl)oxy)carbonyl)amino)-3-cyclohexylpropanoic acid (MPI52d). MPI52d was prepared as a white solid following a general procedure **B**. ^{1}H NMR (400 MHz, Chloroform-d) δ 8.13 (s, 1H), 7.27 (s, 1H), 7.21 (d, J = 4.4 Hz, 2H), 7.15 (d, J = 4.6 Hz, 1H), 5.28 – 4.79 (m, 2H), 4.44 – 4.16 (m, 1H), 1.82 – 1.70 (m, 1H), 1.69 – 1.53 (m, 5H), 1.51 – 1.42 (m, 1H), 1.38 – 1.28 (m, 1H), 1.22 – 1.02 (m, 3H), 0.95 – 0.77 (m, 2H).

(S)-Methyl 2-((S)-2-((((3-chlorobenzyl)oxy)carbonyl)amino)-3-cyclohexylpropanamido)-3-((S)-2-oxopyrrolidin-3-yl)propanoate (MPI52e). MPI50e was prepared with Int.i and MPI52d as a white gummy solid following general procedure **C** (yield 54%). ^{1}H NMR (400 MHz, Chloroform-d) δ 7.87 (d, J = 6.7 Hz, 1H), 7.37 (s, 1H), 7.29 (d, J = 2.8 Hz, 2H), 7.24 (t, J = 4.1 Hz, 1H), 6.01 (s, 1H), 5.39 (d, J = 8.6 Hz, 1H), 5.10 (s, 2H), 4.50 (s, 1H), 4.34 (d, J = 6.7 Hz, 1H), 3.75 (s, 3H), 3.45 – 3.30 (m, 2H), 2.52 – 2.34 (m, 2H), 2.23 – 2.00 (m, 3H), 1.99 – 1.81 (m, 3H), 1.76 – 1.68 (m, 4H), 1.56 – 1.49 (m, 1H), 1.31 – 1.13 (m, 3H), 1.06 – 0.90 (m, 2H).

3-Chlorobenzyl ((S)-3-cyclohexyl-1-(((S)-1-hydroxy-3-((S)-2-oxopyrrolidin-3-yl)propan-2-yl)amino)-1-oxopropan-2-yl)carbamate (MPI52f). MPI52f was prepared as a white solid following a general procedure **D** (yield 80%). ^{1}H NMR (400 MHz, Chloroform-d) δ 7.75 (d, J = 7.2 Hz, 1H), 7.27 (s, 1H), 7.24 (s, 2H), 7.14 (t, J = 4.6 Hz, 1H), 6.16 (s, 1H), 5.52 (d, J = 8.2 Hz, 1H), 5.00 (s, 2H), 4.39 – 4.10 (m, 1H), 4.03 – 3.82 (m, 1H), 3.65 – 3.46 (m, 2H), 3.32 – 3.16 (m, 2H), 2.44 – 2.25 (m, 2H), 2.02 – 1.89 (m, 1H), 1.79 – 1.69 (m, 2H), 1.64 – 1.50 (m, 6H), 1.50 – 1.39 (m, 2H), 1.27 (s, 1H), 1.17 – 1.01 (m, 3H), 0.95 – 0.73 (m, 2H). ^{13}C NMR (101 MHz, $CDCl_3$) δ 181.09, 173.72, 155.96, 138.55, 134.37, 129.82, 128.20, 127.77, 125.82, 65.91, 53.29, 51.21, 40.67, 38.46, 34.12, 33.71, 32.52, 32.03, 30.96, 28.81, 26.38, 26.24, 26.05.

3-(((((S)-3-Cyclohexyl-1-(((S)-1-hydroxy-3-((S)-2-oxopyrrolidin-3-yl)propan-2-yl)amino)-1-oxopropan-2-yl)carbamoyl)oxy)methyl)phenyl acetate (MPI53f). To a stirred solution of 3-(hydroxymethyl)phenyl acetate (100 mg, 0.599 mmol) and DIPEA (0.31 mL, 1.79 mmol) in dry CH_2Cl_2 (10 mL) was added N,N'-disuccinimidyl carbonate (214 mg, 0.838 mmol) at 0 °C. After 10 h at rt, (S)-2-amino-3-cyclohexyl-N-((S)-1-hydroxy-3-((S)-2-oxopyrrolidin-3-yl)propan-2-yl)propanamide (186 mg, 0.599 mmol) was added one portion at 0 °C. After 10 h at rt, the reaction mixture was evaporated in vacuo. Purification by silica gel chromatography (Dichloromethane/MeOH = 9:1). 150 mg of compound isolated. Yield 50%. ^{1}H NMR (400 MHz, Chloroform-d) δ 7.68 (d, J = 8.3 Hz, 1H), 7.33 (t, J = 7.8 Hz, 1H), 7.18 (d, J = 7.6 Hz, 1H), 7.07 (s, 1H), 7.01 (dd, J = 8.0, 2.3 Hz, 1H), 6.43 (d, J = 17.5 Hz, 1H), 5.70 (t, J = 10.0 Hz, 1H), 5.07 (s, 2H), 4.25 (dd, J = 8.9, 5.3 Hz, 1H), 4.02 – 3.90 (m, 1H), 3.57 (q, J = 8.4, 5.4 Hz, 2H), 3.24 (t, J = 8.4 Hz, 2H), 2.45 – 2.30 (m, 2H), 2.28 (s, 3H), 1.99 (ddd, J = 14.2, 11.0, 5.2 Hz, 1H), 1.81 – 1.73 (m, 2H), 1.64 (td, J = 11.0, 8.6, 4.8 Hz, 6H), 1.49 (td, J = 8.9, 8.3, 4.5 Hz, 1H), 1.33 (s, 1H), 1.16 (ddd, J = 25.5, 16.6, 10.9 Hz, 3H), 0.98 – 0.84 (m, 2H). 13C NMR (101 MHz, CDCl3) δ 181.06, 173.63, 169.52, 156.03, 150.76, 138.20, 129.53, 125.15, 121.26, 120.94, 66.09, 65.78, 53.28, 50.75, 40.68, 40.61, 38.37, 34.10, 33.70, 32.52, 32.15, 28.61, 26.40, 26.24, 26.05, 21.13.

Methyl (1R,2S,5S)-3-(2-(2,4-dichlorophenoxy)acetyl)-6,6-dimethyl-3-azabicyclo[3.1.0]hexane-2-carboxylate (MPI55c). MPI55c was prepared with methyl (1R,2S,5S)-6,6-dimethyl-3-

azabicyclo[3.1.0]hexane-2-carboxylate hydrogen chloride (MPI55b) and 2-(2,4-dichlorophenoxy)acetic acid (MPI55a) as a white solid following a general procedure **A** (yield 54%). ^{1}H NMR (400 MHz, Chloroform-d) δ 7.29 (d, J = 2.5 Hz, 1H), 7.14 – 7.02 (m, 1H), 6.79 (dd, J = 8.9, 7.5 Hz, 1H), 4.64 – 4.41 (m, 2H), 4.37 (s, 1H), 3.82 (dd, J = 10.5, 5.3 Hz, 1H), 3.67 (d, J = 1.5 Hz, 1H), 3.64 (d, J = 4.3 Hz, 3H), 1.45 (dd, J = 7.4, 5.1 Hz, 1H), 1.37 (d, J = 7.5 Hz, 1H), 0.98 (d, J = 4.1 Hz, 3H), 0.84 (s, 2H), 0.76 (s, 1H). 13C NMR (100MHz, Chloroform-d) δ 171.82, 171.57, 166.18, 165.75, 152.24, 130.15, 130.10, 127.78, 127.67, 126.72, 123.56, 123.47, 114.52, 114.32, 69.33, 68.48, 59.91, 59.03, 52.75, 52.44, 47.35, 46.25, 32.28, 29.89, 27.61, 26.18, 24.69, 19.53, 19.45, 12.52, 12.33.

Synthesis of (1R,2S,5S)-3-(2-(2,4-dichlorophenoxy)acetyl)-6,6-dimethyl-3-azabicyclo[3.1.0]hexane-2-carboxylic acid (MPI55d). MPI55d was prepared as a white solid following a general procedure **B**. ^{1}H NMR (400 MHz, Methanol-d4) δ 7.30 (dd, J = 4.3, 2.6 Hz, 1H), 7.10 (dd, J = 8.9, 2.6 Hz, 1H), 6.83 (dd, J = 8.9, 6.9 Hz, 1H), 4.82 – 4.69 (m, 2H), 4.24 (s, 1H), 3.79 (dd, J = 10.6, 5.3 Hz, 1H), 3.65 – 3.48 (m, 1H), 1.49 (dd, J = 7.5, 5.2 Hz, 1H), 1.45 – 1.35 (m, 1H), 0.97 (d, J = 3.6 Hz, 3H), 0.87 (s, 2H), 0.81 (s, 1H). ^{13}C NMR (100 MHz, Methanol-d_4) δ 173.04, 166.78, 129.44, 127.41, 126.07, 123.24, 114.71, 67.18, 59.79, 48.29, 48.08, 47.87, 47.65, 47.44, 47.23, 47.01, 45.66, 29.95, 27.21, 25.07, 19.10, 11.54.

Methyl (S)-2-((1R,2S,5S)-3-(2-(2,4-dichlorophenoxy)acetyl)-6,6-dimethyl-3-azabicyclo[3.1.0]hexane-2-carboxamido)-3-((S)-2-oxopyrrolidin-3-yl)propanoate (MPI55e). MPI55e was prepared with Int.i and (MPI55d) as a white gummy solid following general procedure **C.** ^{1}H NMR (400 MHz, Chloroform-d) δ 7.27 (dd, J = 4.6, 2.5 Hz, 1H), 7.13 – 6.97 (m, 1H), 6.79 (dd, J = 22.5, 8.9 Hz, 1H), 4.65 – 4.55 (m, 2H), 4.51 – 4.39 (m, 1H), 4.34 – 4.28 (m, 1H), 3.87 – 3.68 (m, 2H), 3.65 (d, J = 11.1 Hz, 3H), 3.35 – 3.05 (m, 2H), 2.52 – 2.35 (m, 1H), 2.35 – 2.23 (m, 1H), 2.09 – 1.95 (m, 1H), 1.95 – 1.71 (m, 3H), 1.65 – 1.48 (m, 3H), 1.39 – 1.27 (m, 1H), 0.98 (d, J = 4.5 Hz, 3H), 0.81 (d, J = 10.9 Hz, 3H).

(1R,2S,5S)-3-(2-(2,4-Dichlorophenoxy)acetyl)-N-((S)-1-hydroxy-3-((S)-2-oxopyrrolidin-3-yl)propan-2-yl)-6,6-dimethyl-3-azabicyclo[3.1.0]hexane-2-carboxamide (MPI55f). MPI55f was prepared as a white solid following a general procedure **D** (yield 63%). ^{1}H NMR (400 MHz, Chloroform-d) δ 7.34 – 7.24 (m, 1H), 7.15 – 7.02 (m, 1H), 6.88 – 6.60 (m, 1H), 4.64 – 4.51 (m, 2H), 4.05 (dd, J = 5.1, 3.9 Hz, 1H), 4.00 – 3.76 (m, 3H), 3.70 – 3.43 (m, 5H), 3.32 – 3.08 (m, 2H), 2.48 – 2.32 (m, 1H), 2.34 – 2.17 (m, 1H), 2.03 – 1.84 (m, 1H), 1.82 – 1.63 (m, 2H), 1.59 – 1.49 (m, 1H), 0.98 (s, 3H), 0.80 (d, J = 14.5 Hz, 3H). ^{13}C NMR (100 MHz, Chloroform-d) δ 181.24, 171.79, 166.09, 152.43, 130.05, 127.70, 127.62, 126.60, 126.35, 123.61, 114.73, 114.64, 71.03, 68.13, 67.98, 65.19, 61.88, 61.15, 51.35, 46.57, 40.59, 38.21, 31.88, 30.78, 29.06, 27.61, 26.14, 25.62, 19.37, 12.66.

(2S,4S)-1-((Benzyloxy)carbonyl)-4-cyclohexylpyrrolidine-2-carboxylic acid (MPI56d). MPI56d was prepared as a white solid following a general procedure **G**. ^{1}H NMR (400 MHz, Chloroform-d) δ 7.43 – 7.28 (m, 5H), 5.37 – 5.04 (m, 2H), 4.46 (d, J = 8.9 Hz, 1H), 3.75 – 3.60 (m, 1H), 3.13 – 2.98 (m, 1H), 2.42 (dd, J = 12.8, 6.2 Hz, 1H), 2.05 (d, J = 7.4 Hz, 1H), 1.80 – 1.54 (m, 6H), 1.16 (dq, J = 16.7, 5.9 Hz, 5H), 1.03 – 0.82 (m, 2H).

(2S,4S)-Benzyl 4-cyclohexyl-2-(((S)-1-methoxy-1-oxo-3-((S)-2-oxopyrrolidin-3-yl)propan-2-yl)carbamoyl)pyrrolidine-1-carboxylate (MPI56e). MPI56e was prepared with **Int.i** and MPI56d as a white gummy solid following general procedure **C** (yield 60%). ^{1}H NMR (400 MHz, Chloroform-d) δ 7.74 (s, 0H), 7.54 (d, J = 7.2 Hz, 1H), 7.42 – 7.27 (m, 5H), 6.04 (dd, J = 54.4, 22.8 Hz, 1H), 5.14 (s, 2H), 4.55 (s, 1H), 4.47 – 4.32 (m, 1H), 3.85 – 3.58 (m, 5H), 3.31 – 3.21 (m, 2H), 2.52 – 2.01 (m, 5H), 1.90 – 1.53 (m, 9H), 1.26 – 1.06 (m, 5H), 1.03 – 0.85 (m, 2H).

(2S,4S)-Benzyl 4-cyclohexyl-2-(((S)-1-hydroxy-3-((S)-2-oxopyrrolidin-3-yl)propan-2-yl)carbamoyl)pyrrolidine-1-carboxylate (MPI56f). MPI56f was prepared as a white solid following a general procedure **D** (yield 59%). ^{1}H NMR (400 MHz, Chloroform-d) δ 7.80 (t, J = 8.4 Hz, 1H), 7.36 – 7.22 (m, 5H), 5.06 (d, J = 9.6 Hz, 2H), 4.39 – 4.16 (m, 1H), 4.00 – 3.81 (m, 1H), 3.74 (dd, J = 10.2, 7.7 Hz, 1H), 3.49 – 3.31 (m, 2H), 3.25 – 3.13 (m, 2H), 3.07 – 2.91 (m, 2H), 2.51 – 2.22 (m, 1H), 2.11 – 1.99 (m, 3H), 1.94 – 1.73 (m, 2H), 1.71 – 1.56 (m, 7H), 1.47 (ddd, J = 14.7, 10.8, 3.6 Hz, 1H), 1.27 – 1.09 (m, 5H), 1.00 – 0.88 (m, 2H).

Synthesis of 3-benzyl 2-methyl (1R,2S,5S)-6,6-dimethyl-3-azabicyclo[3.1.0]hexane-2,3-dicarboxylate (MPI57c). To a solution of methyl (1R,2S,5S)-6,6-dimethyl-3-azabicyclo[3.1.0]hexane-2-carboxylate (300 mg, 1.46 mmol) in dichloromethane (20 mL) was added benzyl chloroformate (300 mg, 0.25 mL, 1.75 mmol) dropwise, cooled to 0°C, followed by the addition of DIPEA (566 mg, 0.79 mL, 4.38 mmol). The reaction was allowed to stir at RT for overnight. The product was extracted with ethyl acetate (50 mL) and washed with saturated $NaHCO_3$ solution (2×20 mL), 1 M HCl solution (2×20 mL), and saturated brine solution (2×20 mL) sequentially. The organic layer was dried over anhydrous Na_2SO_4 and then concentrated on vacuo. The residue was then purified with flash chromatography (50-100% EtOAc in hexanes as the eluent) to afford **MPI57c** as white solid (380 mg, 77%). ^{1}H NMR (400 MHz, Chloroform-d) δ 7.24 – 7.15 (m, 5H), 5.13 – 4.86 (m, 3H), 4.16 (d, J = 23.4 Hz, 1H), 3.65 (d, J = 14.5 Hz, 3H), 3.43 (dd, J = 13.9, 10.9 Hz, 1H), 1.32 (d, J = 4.5 Hz, 3H), 0.95 (s, 4H), 0.87 (d, J = 1.9 Hz, 4H). ^{13}C NMR (100 MHz, Chloroform-d) δ 154.18, 153.60, 136.68, 136.58, 128.45, 128.39, 127.91, 127.63, 127.59, 66.96, 66.90, 59.87, 59.53, 52.31, 52.29, 52.16, 46.89, 46.34, 32.03, 31.05, 27.32, 26.48, 26.26, 26.25, 19.40, 19.36, 12.56.

Synthesis of (2S,4R)-1-((benzyloxy)carbonyl)-4-(tert-butoxy)pyrrolidine-2-carboxylic acid (MPI58d). **MPI58d** was prepared as a white solid following a general procedure **G**. ^{1}H NMR (400 MHz, Chloroform-d) δ 7.33 – 7.16 (m, 5H), 5.18 – 4.92 (m, 2H), 4.45 – 4.34 (m, 1H), 4.27 – 4.16 (m, 1H), 3.71 – 3.60 (m, 1H), 3.35 – 3.15 (m, 1H), 2.21 – 1.97 (m, 2H), 1.16 (t, J = 7.2 Hz, 1H), 1.09 (d, J = 2.1 Hz, 9H). ^{13}C NMR (100 MHz, Chloroform-d) δ 177.30, 176.38, 155.60, 154.47, 136.33, 136.30, 128.51, 128.40, 128.11, 127.89, 127.57, 74.30, 69.14, 68.48, 67.51, 67.27, 60.55, 57.92, 57.54, 53.82, 53.20, 38.49, 37.29, 28.23, 14.18.

Synthesis of benzyl (2S,4R)-4-(tert-butoxy)-2-(((S)-1-methoxy-1-oxo-3-((S)-2-oxopyrrolidin-3-yl)propan-2-yl)carbamoyl)pyrrolidine-1-carboxylate--methane (MPI58e). MPI58e was prepared with **Int.i** and MPI58d as a white gummy solid following general procedure **C** (yield 87%). ^{1}H NMR (400 MHz, DMSO-d6) δ 8.77 (dd, J = 4.4, 1.5 Hz, 1H), 8.62 – 8.49 (m, 2H), 7.62 (d, J = 3.5 Hz, 1H), 7.52 (dd, J = 8.4, 4.4 Hz, 1H), 7.42 – 7.22 (m, 5H), 5.13 – 4.92 (m, 2H), 4.42 – 4.22 (m, 3H), 3.65 – 3.57 (m, 4H), 3.23 – 3.02 (m, 3H), 2.16 – 1.86 (m, 5H), 1.66 – 1.47 (m, 2H), 1.26 (dd, J = 6.9, 4.5 Hz, 4H), 1.13 (d, J = 3.9 Hz, 9H). ^{13}C NMR (100 MHz, DMSO-d6) δ 178.57, 178.27, 172.80, 172.76, 172.72, 172.43, 162.78, 154.21, 151.50, 140.08, 137.46, 137.34, 135.10, 129.29, 128.84, 128.66, 128.23, 128.00, 127.82, 127.24, 121.15, 73.96, 73.93, 68.67, 66.29, 58.89, 58.49, 54.06, 52.42, 50.77, 42.31, 38.72, 38.36, 38.01, 37.93, 36.25, 32.88, 32.72, 31.24, 28.48, 27.45, 18.56, 17.20, 12.96.

Synthesis of benzyl (2S,4R)-4-(tert-butoxy)-2-(((S)-1-hydroxy-3-((S)-2-oxopyrrolidin-3-yl)propan-2-yl)carbamoyl)pyrrolidine-1-carboxylate--methane (MPI58f). MPI58f was prepared as a white solid following a general procedure **D**. Yield (59%). ^{1}H NMR (400 MHz, Chloroform-d) δ 7.42 (d, J = 7.7 Hz, 1H), 7.26 (d, J = 3.7 Hz, 5H), 6.04 (d, J = 11.2 Hz, 1H), 5.19 – 4.94 (m, 2H), 4.36 – 4.15 (m, 2H), 3.86 (d, J = 55.4 Hz, 1H), 3.73 – 3.52 (m, 2H), 3.50 – 3.31 (m, 2H), 3.22 (dd, J = 10.3, 4.2 Hz, 3H), 2.43 (s, 1H), 2.28 (s, 1H), 2.18 – 1.85 (m, 4H), 1.72 (p, J = 9.6 Hz, 2H), 1.51 (dd, J = 18.9, 10.4 Hz, 1H), 1.46 – 1.32 (m, 1H), 1.10 (s, 10H). ^{13}C NMR (100 MHz, Chloroform-d) δ 181.08, 172.94, 155.66, 154.88, 136.54, 128.49, 128.01, 127.73, 74.03, 69.41, 67.20, 65.38, 59.86, 53.61, 50.80, 40.55, 37.73, 31.86, 28.84, 28.27.

Synthesis of (1R,2S,5S)-3-((benzyloxy)carbonyl)-6,6-dimethyl-3-azabicyclo[3.1.0]hexane-2-carboxylic acid (MPI57d). MPI57d was prepared as a white solid following a general procedure **B**. ^{1}H NMR (400 MHz, Chloroform-d) δ 7.30 – 7.13 (m, 5H), 5.13 – 4.94 (m, 2H), 4.22 (s, 1H), 3.69 – 3.59 (m, 1H), 3.46 (dd, J = 15.5, 11.0 Hz, 1H), 1.46 (dd, J = 20.2, 7.4 Hz, 1H), 1.38 – 1.32 (m, 1H), 0.98 (d, J = 1.8 Hz, 3H), 0.89 (s, 3H). ^{13}C NMR (100 MHz, Chloroform-d) δ 177.88, 176.93, 154.75, 153.80, 136.40, 128.52, 128.43, 128.06, 127.90, 127.67, 127.50, 67.37, 67.19, 59.86, 59.34, 46.46, 32.00, 30.86, 27.25, 26.43, 26.30, 26.27, 19.54, 19.43, 12.59.

Synthesis of benzyl (1R,2S,5S)-2-((1-methoxy-1-oxo-3-(2-oxopyrrolidin-3-yl)propan-2-yl)carbamoyl)-6,6-dimethyl-3-azabicyclo[3.1.0]hexane-3-carboxylate (MPI57e). MPI56e was prepared with **Int.i** and MPI56d as a white gummy solid following general procedure **C** (yield 82%). ^{1}H NMR (400 MHz, Chloroform-d) δ 7.29 – 7.12 (m, 5H), 5.12 – 4.95 (m, 2H), 4.37 – 4.21 (m, 1H), 4.08 (d, J = 2.9 Hz, 1H), 3.79 – 3.61 (m, 3H), 3.61 – 3.38 (m, 3H), 3.30 – 3.05 (m, 2H), 2.48 – 1.89 (m, 4H), 1.88 – 1.60 (m, 2H), 1.53 – 1.26 (m, 3H), 0.96 (d, J = 1.7 Hz, 3H), 0.84 (d, J = 2.4 Hz, 3H). ^{13}C NMR (100 MHz, Chloroform-d) δ 179.87, 179.85, 172.64, 172.30, 172.10, 162.58, 154.52, 153.99, 136.72, 136.65, 128.46, 128.33, 127.92, 127.71, 127.53, 127.49, 67.05, 66.99, 61.27, 52.47, 52.37, 52.09, 51.35, 47.35, 38.63, 38.56, 36.51, 33.26, 32.80, 31.45, 31.19, 28.67, 26.40, 19.29, 19.14, 12.68, 12.60.

Synthesis of benzyl (1R,2S,5S)-2-((1-hydroxy-3-(2-oxopyrrolidin-3-yl)propan-2-yl)carbamoyl)-6,6-dimethyl-3-azabicyclo[3.1.0]hexane-3-carboxylate (MPI57f). MPI57f was prepared as a white solid following a general procedure **D** (yield 61%). ^{1}H NMR (400 MHz, Chloroform-d) δ 7.36 – 7.15 (m, 6H), 5.30 – 4.90 (m, 2H), 4.01 – 3.79 (m, 1H), 3.76 – 3.58 (m, 2H), 3.52 – 3.36 (m, 2H), 3.26 – 3.08 (m, 2H), 2.47 – 2.07 (m, 2H), 2.00 – 1.63 (m, 2H), 1.59 – 1.26 (m, 3H), 0.96 (s, 3H), 0.84 (d, J = 4.3 Hz, 3H). ^{13}C NMR (100 MHz, Chloroform-d) δ 181.08, 180.89, 173.46, 172.64, 154.68, 154.11, 136.56, 128.50, 128.47, 128.01, 127.99, 127.74, 127.60, 67.17, 67.07, 66.08, 65.42, 61.90, 51.43, 50.89, 47.34, 46.90, 40.60, 38.38, 38.16, 33.09, 31.95, 31.84, 31.64, 28.82, 28.74, 27.40, 26.23, 26.15, 19.27, 19.22, 12.66, 12.56.

(S)-N-((S)-1-Hydroxy-3-((S)-2-oxopyrrolidin-3-yl)propan-2-yl)-6-azaspiro[3.4]octane-7-carboxamide hydrogen chloride (MPI-59i-1). To a stirred solution of **YR-B-101c** (200 mg, 0.506 mmol) in 1,4-Dioxane (2 mL) at 0 °C was added 4N HCl (1.26 mL, 5.06 mmol). Reaction mixture was stirred at rt for 3 h. After completion of reaction, solvent was concentrated in a vacuum. The residue was used in the next step without further purification. (150 mg). Crude product proceeded for next step without purification.

Benzyl (S)-7-(((S)-1-hydroxy-3-((S)-2-oxopyrrolidin-3-yl)propan-2-yl)carbamoyl)-6-azaspiro[3.4]octane-6-carboxylate (MPI59f). MPI59f was prepared by using procedure of **MPI57c.** Yield (54%). ^{1}H NMR (400 MHz, CDCl3) δ 7.36 (s, 5H), 5.67 (d, J = 28.6 Hz, 1H), 5.25 – 4.99 (m, 2H), 4.25 (dd, J = 8.1, 6.0 Hz, 1H), 4.11 – 3.70 (m, 2H), 3.53 (s, 2H), 3.42 (dd, J = 11.3, 5.0 Hz, 1H), 3.36 – 3.24 (m, 2H), 2.41 (s, 1H), 2.30 – 2.11 (m, 3H), 2.05 – 1.78 (m, 9H).

tert-Butyl 3-(((S)-1-methoxy-1-oxo-3-((S)-2-oxopyrrolidin-3-yl)propan-2-yl)carbamoyl)-2-azaspiro[4.4]nonane-2-carboxylate (MPI60e). MPI60e was prepared with **Int.i** and 2-(tert-butoxycarbonyl)-2-azaspiro[4.4]nonane-3-carboxylic acid as a white gummy solid following general procedure **C** (yield 69%). ^{1}H NMR (400 MHz, DMSO) δ 8.41 (dd, J = 19.5, 7.8 Hz, 1H), 7.64 (d, J = 34.9

Hz, 1H), 4.35 – 4.18 (m, 1H), 4.13 (t, J = 7.8 Hz, 1H), 3.62 (s, 3H), 3.25 (d, J = 10.8 Hz, 1H), 3.12 (td, J = 19.7, 9.1 Hz, 3H), 2.31 – 1.93 (m, 4H), 1.80 – 1.50 (m, 9H), 1.50 – 1.25 (m, 11H).

Benzyl 3-(((S)-1-methoxy-1-oxo-3-((S)-2-oxopyrrolidin-3-yl)propan-2-yl)carbamoyl)-2-azaspiro[4.4]nonane-2-carboxylate (MPI60e-1). MPI60e-1 was prepared with as a white gummy solid following general procedures **F** and **G** (yield 77%). ^{1}H NMR (400 MHz, DMSO) δ 8.50 (dd, J = 13.0, 7.8 Hz, 1H), 7.61 (s, 1H), 7.41 – 7.23 (m, 5H), 5.11 – 4.91 (m, 2H), 4.40 – 4.18 (m, 2H), 3.61 (d, J = 12.8 Hz, 3H), 3.37 (d, J = 10.2 Hz, 2H), 3.22 (t, J = 10.0 Hz, 1H), 3.15 – 3.03 (m, 1H), 2.20 – 1.86 (m, 4H), 1.75 (td, J = 13.2, 7.8 Hz, 1H), 1.64 – 1.39 (m, 10H).

Benzyl 3-(((S)-1-hydroxy-3-((S)-2-oxopyrrolidin-3-yl)propan-2-yl)carbamoyl)-2-azaspiro[4.4]nonane-2-carboxylate (MPI60f). MPI60f was prepared as a white solid following a general procedure **D**. Yield (58%). ^{1}H NMR (400 MHz, $CDCl_3$) δ 7.79 (d, J = 6.9 Hz, 1H), 7.31 – 7.16 (m, 5H), 6.15 (d, J = 52.2 Hz, 1H), 5.16 – 4.91 (m, 2H), 4.20 (t, J = 7.8 Hz, 1H), 3.88 (d, J = 55.0 Hz, 1H), 3.63 – 3.12 (m, 6H), 2.44 – 2.03 (m, 3H), 1.94 (d, J = 7.6 Hz, 2H), 1.85 – 1.31 (m, 10H).

tert-Butyl 3-(((S)-1-methoxy-1-oxo-3-((S)-2-oxopyrrolidin-3-yl)propan-2-yl)carbamoyl)-2-azaspiro[4.5]decane-2-carboxylate (MPI61e). MPI61e was prepared with **Int.i** and 2-(tert-butoxycarbonyl)-2-azaspiro[4.5]decane-3-carboxylic acid a white gummy solid following general procedure **C** (yield 75%). ^{1}H NMR (400 MHz, $CDCl_3$) δ 6.38 (d, J = 141.5 Hz, 1H), 4.46 (d, J = 60.3 Hz, 1H), 4.18 (dd, J = 8.5, 7.3 Hz, 1H), 3.65 (d, J = 2.7 Hz, 3H), 3.33 – 3.21 (m, 2H), 3.14 – 2.99 (m, 1H), 2.48 – 2.27 (m, 2H), 2.11 (ddd, J = 13.2, 10.5, 4.8 Hz, 2H), 1.82 (dp, J = 11.5, 4.1 Hz, 3H), 1.35 (d, J = 18.7 Hz, 19H).

Benzyl 3-(((S)-1-methoxy-1-oxo-3-((S)-2-oxopyrrolidin-3-yl)propan-2-yl)carbamoyl)-2-azaspiro[4.5]decane-2-carboxylate (MPI61e-1). MPI61e-1 was prepared with as a white gummy solid following general procedures **F** and **G** (yield 90%). ^{1}H NMR (400 MHz, DMSO-d_6) δ 8.57 (ddd, J = 20.3, 10.2, 7.2 Hz, 1H), 7.70 (dd, J = 12.9, 4.7 Hz, 1H), 7.49 – 7.30 (m, 6H), 5.14 (q, J = 6.3, 4.8 Hz, 1H), 5.13 – 4.98 (m, 1H), 4.46 – 4.23 (m, 2H), 3.73 – 3.65 (m, 2H), 3.63 (s, 1H), 3.56 – 3.47 (m, 1H), 3.31 – 3.17 (m, 1H), 3.20 – 3.13 (m, 1H), 3.15 – 2.89 (m, 1H), 2.17 (s, 2H), 2.28 – 2.02 (m, 1H), 1.75 – 1.57 (m, 2H), 1.66 (s, 2H), 1.51 (d, J = 17.1 Hz, 5H), 1.42 (d, J = 14.5 Hz, 8H). ^{13}C NMR (101 MHz, DMSO) δ 22.04, 22.59, 24.93, 33.71, 34.57, 36.95, 37.61, 40.14, 40.99, 49.41, 51.28, 57.64, 65.17, 126.06, 126.64, 126.84, 127.55, 136.34, 153.33, 153.44, 171.66, 177.31.

Benzyl 3-(((S)-1-hydroxy-3-((S)-2-oxopyrrolidin-3-yl)propan-2-yl)carbamoyl)-2-azaspiro[4.5]decane-2-carboxylate (MPI61f). MPI61f was prepared as a white solid following a general procedure **D**. Yield (52%). ^{1}H NMR (400 MHz, CDCl3) δ 7.33-7.19 (m, 5H), 6.40-6.06 (m, 1H), 5.16 –

4.87 (m, 2H), 4.29-4.13 (m, 1H), 3.98-3.75 (m, 1H), 3.65 – 3.05 (m, 6H), 2.48 – 2.21 (m, 1H), 2.21 – 1.60 (m, 6H), 1.48 – 1.16 (m, 10H).

Synthesis of methyl (1R,2S,5S)-3-((4-chlorophenyl)glycyl)-6,6-dimethyl-3-azabicyclo[3.1.0]hexane-2-carboxylate (MPI62c). MPI62c was prepared as a white solid following a general procedure **A**. Yield (69%). ^{1}H NMR (400 MHz, Chloroform-d) δ 7.12 – 6.94 (m, 2H), 6.50 – 6.35 (m, 2H), 3.83 – 3.75 (m, 1H), 3.75 – 3.67 (m, 5H), 1.56 – 1.46 (m, 1H), 1.46 – 1.34 (m, 1H), 1.01 (d, J = 3.6 Hz, 3H), 0.87 (d, J = 14.6 Hz, 3H).

Synthesis of (1R,2S,5S)-3-((4-chlorophenyl)glycyl)-6,6-dimethyl-3-azabicyclo[3.1.0]hexane-2-carboxylic acid (MPI62d). To a stirred solution of 2 (300 mg, 0.1 mmol) in 1,4-dioxane (8 mL) was added a 4 M HCl solution in dioxane (8 mL). The reaction mixture was stirred at rt for 1 h and then concentrated in vacuo to get product **MPI62d**. ^{1}H NMR (400 MHz, DMSO-d6) δ 7.07 (dd, J = 8.9, 2.4 Hz, 2H), 6.67 – 6.53 (m, 2H), 5.95 – 5.80 (m, 1H), 3.87 (d, J = 5.4 Hz, 1H), 3.63 (s, 2H), 1.63 – 1.53 (m, 1H), 1.41 (dd, J = 7.5, 3.4 Hz, 1H), 1.35 (d, J = 3.9 Hz, 1H), 1.27 – 1.15 (m, 1H), 1.03 (d, J = 2.0 Hz, 3H), 0.89 (d, J = 9.8 Hz, 3H).

Synthesis of tert-butyl (S)-6-(((S)-1-methoxy-1-oxo-3-((S)-2-oxopyrrolidin-3-yl)propan-2-yl)carbamoyl)-5-azaspiro[2.4]heptane-5-carboxylate (MPI63c). **MPI63c** was prepared with (S)-5-(tert-butoxycarbonyl)-5-azaspiro[2.4]heptane-6-carboxylic acid (MPI63a) and Int.i as a white solid following a general procedure **C** (yield 82%).

Synthesis of methyl (S)-3-((S)-2-oxopyrrolidin-3-yl)-2-((S)-5-azaspiro[2.4]heptane-6-carboxamido)propanoate (MPI63i). **MPI63i** was prepared as a white solid following a general procedure **F**.

Synthesis of methyl (S)-2-((S)-5-(2-(2,4-dichlorophenoxy)acetyl)-5-azaspiro[2.4]heptane-6-carboxamido)-3-((S)-2-oxopyrrolidin-3-yl)propanoate (MPI63e). MPI63e was prepared with 2-(2,4-dichlorophenoxy)acetic acid and Int.i as a white solid following a general procedure **C** (yield 44%). ^{1}H NMR (400 MHz, Chloroform-d) δ 7.28 (dd, J = 5.4, 2.5 Hz, 1H), 6.86 (d, J = 8.9 Hz, 1H), 4.92 – 4.25 (m, 4H), 3.64 (d, J = 8.9 Hz, 3H), 3.32 – 3.13 (m, 2H), 2.52 – 2.35 (m, 1H), 2.35 – 2.19 (m, 1H), 2.19 – 1.92 (m, 2H), 1.92 – 1.63 (m, 4H), 1.48 – 1.24 (m, 2H), 0.70 – 0.52 (m, 3H), 0.52 – 0.36 (m, 1H).

Synthesis of (S)-5-(2-(2,4-dichlorophenoxy)acetyl)-N-((S)-1-hydroxy-3-((S)-2-oxopyrrolidin-3-yl)propan-2-yl)-5-azaspiro[2.4]heptane-6-carboxamide (MPI63f). **MPI63f** was prepared as a white solid following a general procedure **D**. (Yield 68%). ^{1}H NMR (400 MHz, Chloroform-d) δ 7.91 (dd, J = 7.3, 3.5 Hz, 1H), 7.28 (t, J = 2.3 Hz, 1H), 7.14 – 7.00 (m, 1H), 6.87 (d, J = 8.8 Hz, 1H), 4.79 – 4.65 (m,

1H), 4.62 – 4.46 (m, 1H), 3.93 – 3.78 (m, 1H), 3.71 – 3.58 (m, 1H), 3.58 – 3.33 (m, 3H), 3.32 – 3.14 (m, 2H), 2.51 – 2.22 (m, 3H), 2.16 (dd, J = 12.8, 8.6 Hz, 1H), 2.01 – 1.91 (m, 1H), 1.91 – 1.81 (m, 1H), 1.81 – 1.71 (m, 1H), 1.55 (ddt, J = 19.3, 14.5, 4.6 Hz, 2H), 1.45 – 1.32 (m, 2H), 1.19 (d, J = 1.7 Hz, 1H), 0.64 – 0.43 (m, 4H).

Methyl (1*R*,2*S*,5*S*)-3-(2-(cyclohexyloxy)acetyl)-6,6-dimethyl-3-azabicyclo[3.1.0]hexane-2-carboxylate (MPI64c). MPI64c was prepared as a white solid following a general procedure **A**. Yield (67%). ^{1}H NMR (400 MHz, DMSO) δ 4.21 – 3.81 (m, 3H), 3.77 – 3.63 (m, 4H), 3.58 – 3.44 (m, 1H), 3.31 – 3.19 (m, 1H), 1.90 – 1.74 (m, 2H), 1.72 – 1.59 (m, 2H), 1.58 – 1.51 (m, 1H), 1.50 – 1.37 (m, 2H), 1.20 (tq, *J* = 9.8, 3.1 Hz, 5H), 1.02 (s, 3H), 0.88 (d, *J* = 4.6 Hz, 3H).

(1*R*,2*S*,5*S*)-3-(2-(Cyclohexyloxy)acetyl)-6,6-dimethyl-3-azabicyclo[3.1.0]hexane-2-carboxylic acid (MPI64d). MPI64d was prepared with as a white gummy solid following general procedure **B**. ^{1}H NMR (400 MHz, $CDCl_3$) δ 4.64-4,32(m, 1H), 4.09 – 3.88 (m, 2H), 3.80 – 3.52 (m, 2H), 3.34-3,15 (m, 1H), 1.90 – 1.76 (m, 2H), 1.64 (dd, *J* = 9.0, 6.2 Hz, 2H), 1.49 – 1.39 (m, 2H), 1.32 – 1.07 (m, 6H), 1.00 (s, 3H), 0.88 (s, 3H).

Methyl (*S*)-2-((1*R*,2*S*,5*S*)-3-(2-(cyclohexyloxy)acetyl)-6,6-dimethyl-3-azabicyclo[3.1.0]hexane-2-carboxamido)-3-((*S*)-2-oxopyrrolidin-3-yl)propanoate (MPI64e). MPI64e was prepared with MPI64d and Int.i as a white solid following a general procedure **C** (yield 75%). ^{1}H NMR (400 MHz, $CDCl_3$) δ 8.81 – 8.19 (m, 1H), 7.67-7.24 (m, 1H), 4.53 – 4.25 (m, 2H), 4.05 – 3.95 (m, 2H), 3.77 (dd, *J* = 10.6, 5.1 Hz, 1H), 3.72 – 3.68 (m, 1H), 3.66 (s, 3H), 3.33 – 3.20 (m, 3H), 2.39-2.30 (m, 1H), 2.17 – 2.07 (m, 1H), 1.88-1.80 (m, 3H), 1.49 – 1.43 (m, 2H), 1.31 – 1.07 (m, 9H), 0.98 (s, 3H), 0.85 (s, 3H).

methyl (1R,2S,5S)-3-(3-cyclohexylpropanoyl)-6,6-dimethyl-3-azabicyclo[3.1.0]hexane-2-carboxylate (MPI65c). MPI65c was prepared as a white solid following a general procedure **A**. Yield (88%). ^{1}H NMR (400 MHz, $CDCl_3$) δ 4.38 (s, 1H), 3.82 (dd, *J* = 10.1, 5.3 Hz, 1H), 3.75 (s, 3H), 3.48 (d, *J* = 10.1 Hz, 1H), 2.29 – 2.19 (m, 2H), 1.74 – 1.58 (m, 5H), 1.57 – 1.44 (m, 3H), 1.41 (d, *J* = 7.4 Hz, 1H), 1.31 – 1.09 (m, 5H), 1.05 (s, 3H), 0.95 (s, 3H), 0.92 – 0.81 (m, 2H).

(1R,2S,5S)-3-(3-Cyclohexylpropanoyl)-6,6-dimethyl-3-azabicyclo[3.1.0]hexane-2-carboxylic acid (MPI65d). MPI65d was prepared as a white solid following a general procedure **B**.

methyl (S)-2-((1R,2S,5S)-3-(3-Cyclohexylpropanoyl)-6,6-dimethyl-3-azabicyclo[3.1.0]hexane-2-carboxamido)-3-((S)-2-oxopyrrolidin-3-yl)propanoate (MPI65e). MPI65e was prepared with MPI65d and Int.i as a white solid following a general procedure **C** (yield 73%).^{1}H NMR (400 MHz, $CDCl_3$) δ 7.59 (d, *J* = 7.2 Hz, 1H), 5.92 (s, 1H), 4.58 (ddd, *J* = 11.0, 7.2, 4.2 Hz, 1H), 4.33 (s, 1H), 3.85 (dd, *J* = 10.3, 5.3

Hz, 1H), 3.75 (s, 3H), 3.54 – 3.47 (m, 1H), 3.44 – 3.32 (m, 2H), 2.44 (td, *J* = 7.9, 3.9 Hz, 1H), 2.31 – 2.11 (m, 3H), 2.02 – 1.81 (m, 2H), 1.69 (t, *J* = 9.8 Hz, 4H), 1.58 (d, *J* = 7.6 Hz, 1H), 1.32 – 1.11 (m, 4H), 1.07 (s, 3H), 0.95 (s, 3H), 0.88 (d, *J* = 11.7 Hz, 2H).

(1R,2S,5S)-3-(3-Cyclohexylpropanoyl)-N-((S)-1-hydroxy-3-((S)-2-oxopyrrolidin-3-yl)propan-2-yl)-6,6-dimethyl-3-azabicyclo[3.1.0]hexane-2-carboxamide (MPI65f). **MPI65f** was prepared as a white solid following a general procedure **D**. (Yield 66%). ^{1}H NMR (400 MHz, $CDCl_3$) δ 7.56 (d, *J* = 7.2 Hz, 1H), 5.93 (d, *J* = 32.5 Hz, 1H), 4.25 (s, 1H), 3.98 (tt, *J* = 7.2, 4.0 Hz, 1H), 3.90 (dd, *J* = 10.3, 5.3 Hz, 1H), 3.76 (ddd, *J* = 11.6, 3.9, 2.2 Hz, 1H), 3.54 – 3.43 (m, 2H), 3.33 (dd, *J* = 9.2, 4.4 Hz, 2H), 2.55 – 2.47 (m, 1H), 2.44 – 2.35 (m, 1H), 2.24 (dq, *J* = 18.2, 7.5 Hz, 2H), 2.03 (ddd, *J* = 14.5, 10.7, 6.6 Hz, 1H), 1.89 – 1.74 (m, 1H), 1.70 – 1.59 (m, 6H), 1.53 – 1.44 (m, 4H), 1.18 (tdd, *J* = 20.8, 12.4, 9.4 Hz, 5H), 1.04 (d, *J* = 2.4 Hz, 3H), 0.91 (s, 3H), 0.88 – 0.78 (m, 2H).

tert-Butyl 3-(((S)-1-amino-1-oxo-3-((S)-2-oxopyrrolidin-3-yl)propan-2-yl)carbamoyl)-2-azaspiro[4.4]nonane-2-carboxylate (MPI66-1h). **MPI66-1h** was prepared with MPI66-1g and Int.ii as a white solid following a general procedure **C** (yield 73%).. ^{1}H NMR (400 MHz, DMSO) δ 8.22 – 7.88 (m, 1H), 7.62 (d, J = 12.6 Hz, 1H), 7.24 (d, J = 44.3 Hz, 1H), 7.05 (s, 1H), 4.29 – 4.18 (m, 1H), 4.18 – 4.05 (m, 1H), 3.30 – 3.04 (m, 4H), 2.40 – 1.90 (m, 4H), 1.78 – 1.41 (m, 10H), 1.33 (t, J = 20.8 Hz, 9H).

N-((S)-1-amino-1-oxo-3-((S)-2-oxopyrrolidin-3-yl) propan-2-yl)-2-azaspiro[4.4]nonane-3-carboxamide (MPI66-1i). **MPI66-1i** was prepared as a white solid following a general procedure **F** (Yield 80%).

Benzyl3-(((S)-1-amino-1-oxo-3-((S)-2-oxopyrrolidin-3-yl)propan-2-yl)carbamoyl)-2-azaspiro[4.4]nonane-2-carboxylate (MPI66-1k). **MPI66-1k** was prepared as a white solid following a general procedure **G** (Yield 75%). ^{1}H NMR (400 MHz, DMSO) δ 8.28 – 8.09 (m, 1H), 7.56 (t, J = 14.3 Hz, 1H), 7.41 – 7.17 (m, 5H), 7.03 (d, J = 16.2 Hz, 1H), 5.09 – 4.91 (m, 2H), 4.39 – 4.17 (m, 2H), 3.32 – 2.80 (m, 4H), 2.39 – 2.28 (m, 1H), 2.21 – 1.88 (m, 3H), 1.73 (dt, J = 19.2, 6.1 Hz, 1H), 1.65 – 1.34 (m, 10H).

3-Chlorobenzyl 3-(((S)-1-amino-1-oxo-3-((S)-2-oxopyrrolidin-3-yl)propan-2-yl)carbamoyl)-2-azaspiro[4.4]nonane-2-carboxylate (MPI66-2k). To 3-chlorobenzyl alcohol (0.3 g, 2.104 mmol) in CH_3CN (10 mL) were added DIPEA (1.1 mL, 6.311 mmol) and N,N'-disuccinimidyl carbonate (753 mg, 2.94 mmol) at 0 °C. After 10 h at rt, **MPI66-2i** (750 mg, 2.104 mmol) was added one portion at 0 °C. After 10 h at rt, the reaction mixture was evaporated in vacuo. Purification by silica gel chromatography (Dichloromethane/MeOH = 9:1). 350 mg of compound isolated. Yield 50%. ^{1}H NMR (400 MHz, DMSO) δ 8.37 – 8.10 (m, 1H), 7.72 – 7.52 (m, 1H), 7.43 – 6.97 (m, 5H), 5.13 – 4.90 (m, 2H), 4.46 – 4.17 (m, 2H),

3.38 (t, J = 11.3 Hz, 1H), 3.20 (d, J = 10.7 Hz, 1H), 3.16 – 2.89 (m, 2H), 2.13 (ddd, J = 25.8, 12.5, 7.8 Hz, 2H), 1.84 – 1.24 (m, 13H).

N-((S)-1-Hydroxy-3-((S)-2-oxopyrrolidin-3-yl)propan-2-yl)-2-(4-methoxy-1H-indole-2-carbonyl)-2-azaspiro[4.4]nonane-3-carboxamide (MPI66-3f). MPI66-3f was prepared by **4-methoxy-1H-indole-2-carboxylic acid** (0.345 mmol, 66 mg) and **N-((S)-1-hydroxy-3-((S)-2-oxopyrrolidin-3-yl)propan-2-yl)-2-azaspiro[4.4]nonane-3-carboxamide** (0.345 mmol, 120 mg) in general procedure **C** (Yield 36%). ^{1}H NMR (400 MHz, $CDCl_3$) δ 10.95 (s, 0.5H), 10.14 (s, 0.5H), 7.65 (s, 1H), 7.19 – 6.83 (m, 3H), 6.46 (d, J = 7.7 Hz, 1H), 4.64 (d, J = 67.9 Hz, 1H), 4.19 – 4.06 (m, 1H), 3.95 (d, J = 5.8 Hz, 3H), 3.91 – 3.76 (m, 2H), 3.68 (d, J = 16.7 Hz, 1H), 3.51 (s, 1H), 3.14 (s, 1H), 2.93 (s, 1H), 2.58 – 2.31 (m, 2H), 2.27 – 1.91 (m, 3H), 1.80 – 1.23 (m, 10H).

tert-Butyl 3-(((S)-1-amino-1-oxo-3-((S)-2-oxopiperidin-3-yl)propan-2-yl)carbamoyl)-2-azaspiro[4.4]nonane-2-carboxylate (MPI66-4h). MPI66-4h was prepared with MPI66-4g and Int.ii as a white solid following a general procedure **C** (yield 82%).. ^{1}H NMR (400 MHz, $CDCl_3$) δ 4.33 (s, 1H), 4.13 (d, J = 9.0 Hz, 1H), 3.24 (h, J = 11.3 Hz, 4H), 2.37 – 1.99 (m, 3H), 1.96 – 1.71 (m, 4H), 1.68 – 1.29 (m, 19H).

N-((S)-1-Amino-1-oxo-3-((S)-2-oxopiperidin-3-yl)propan-2-yl)-2-azaspiro[4.4]nonane-3-carboxamide hydrogen chloride (MPI66-4i). MPI66-4i was prepared as a white solid following a general procedure **F** (150 mg).

N-((S)-1-Amino-1-oxo-3-((S)-2-oxopiperidin-3-yl)propan-2-yl)-2-(4-methoxy-1H-indole-2-carbonyl)-2-azaspiro[4.4]nonane-3-carboxamide (MPI66-4k). MPI66-4k was prepared with MPI66-4i and **4-methoxy-1H-indole-2-carboxylic acid** as a white solid following a general procedure **C** (yield 39%).. ^{1}H NMR (400 MHz, DMSO) δ 11.57 (s, 1H), 8.43 (d, J = 8.5 Hz, 1H), 7.59 (s, 1H), 7.26 (s, 1H), 7.22 – 7.09 (m, 2H), 7.04 (d, J = 8.3 Hz, 1H), 6.93 (s, 1H), 6.53 (d, J = 7.7 Hz, 1H), 4.55 (dd, J = 9.7, 7.0 Hz, 1H), 4.28 – 4.13 (m, 1H), 3.89 (s, 3H), 3.87 – 3.79 (m, 2H), 3.20 – 3.04 (m, 2H), 2.24 – 2.04 (m, 3H), 2.01 – 1.75 (m, 3H), 1.63 (td, J = 7.1, 4.2 Hz, 7H), 1.51 – 1.23 (m, 4H).

Benzyl 3-(((S)-1-amino-1-oxo-3-((S)-2-oxopyrrolidin-3-yl)propan-2-yl)carbamoyl)-2-azaspiro[4.5]decane-2-carboxylate (MPI67k). MPI67k was prepared as a white solid following a general procedure **G** (Yield 79%). ^{1}H NMR (400 MHz, CDCl3) δ 8.46 (d, J = 6.0 Hz, 1H), 7.31 – 7.22 (m, 5H), 7.15 (s, 1H), 6.15 (s, 1H), 5.42 (d, J = 11.8 Hz, 1H), 5.15-5.00 (m, 2H), 4.25 – 4.20 (m, 1H), 3.72 – 3.55 (m, 1H), 3.48 (dd, J = 18.8, 10.0 Hz, 1H), 3.28 (d, J = 8.6 Hz, 2H), 3.16-3.04 (m, 1H), 2.14 (dd, J = 12.7, 8.0 Hz, 2H), 2.10 – 1.96 (m, 2H), 1.95-1.75 (m, 2H), 1.71 – 1.54 (m, 2H), 1.46 – 1.27 (m, 12H).

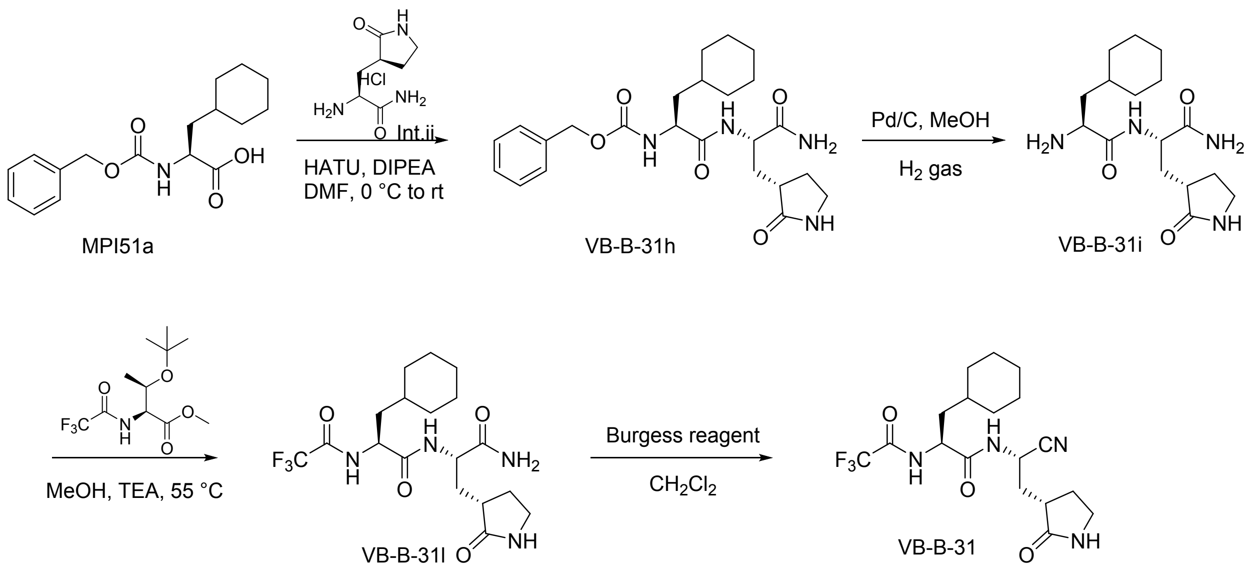

Benzyl ((*S*)-1-(((*S*)-1-amino-1-oxo-3-((*S*)-2-oxopyrrolidin-3-yl)propan-2-yl)amino)-3-cyclohexyl-1-oxopropan-2-yl)carbamate (VB-B-31h). VB-B-31h was prepared with MPI51a and Int.ii as a white solid following a general procedure **C** (yield 82%).^{1}H NMR (400 MHz, $CDCl_3$) δ 8.24 (d, *J* = 6.8 Hz, 1H), 7.31 – 7.17 (m, 5H), 6.97 (s, 1H), 6.74 (s, 1H), 6.10 (s, 1H), 5.92 (d, *J* = 7.3 Hz, 1H), 5.08 – 4.93 (m, 2H), 4.38 (dt, *J* = 10.1, 6.2 Hz, 1H), 4.23 – 4.07 (m, 1H), 3.24-3.08 (m, 2H), 2.31-2.10 (m, 2H), 2.03 – 1.79 (m, 2H), 1.78 – 1.47 (m, 7H), 1.46-1.22 (m, 2H), 1.08 (p, *J* = 11.6 Hz, 3H), 0.93 – 0.68 (m, 2H). ^{13}C NMR (101 MHz, $CDCl_3$) δ 180.70, 174.41, 173.53, 156.64, 136.38, 128.53, 128.14, 127.96, 66.96, 53.58, 52.43, 40.73, 39.96, 38.49, 34.07, 33.75, 32.80, 32.29, 28.46, 26.22, 26.03.

Methyl *O*-(*tert*-butyl)-*N*-(2,2,2-trifluoroethanethioyl)-*L*-threoninate (VB-B-31i).

To a solution of **VB-B-31h** (250 mg, 0.47 mmol) in methanol (10 mL) was added 10% Pd/C (50 mg). The reaction mixture was stirred under H_2 balloon at rt for 3 h. The reaction mixture was filtered with celite, and the filtrate was concentrated *in vacuo* to yield **VB-B-31h** as colorless oil (168 mg, 90%), which was used without further purification. ^{1}H NMR (400 MHz, $CDCl_3$) δ 8.45 (s, 1H), 4.98 – 4.77 (m, 1H), 4.30 (qd, *J* = 6.3, 1.8 Hz, 1H), 3.69 (s, 3H), 1.15 (d, *J* = 6.4 Hz, 3H), 1.08 (s, 9H). ^{13}C NMR (101 MHz, $CDCl_3$) δ 184.85, 184.49, 168.63, 118.77, 115.99, 75.02, 67.14, 63.00, 52.71, 28.23, 21.43.

(*S*)-*N*-((*S*)-1-amino-1-oxo-3-((*S*)-2-oxopyrrolidin-3-yl)propan-2-yl)-3-cyclohexyl-2-(2,2,2-trifluoroethanethioamido)propenamide (VB-B-31l). methyl O-(tert-butyl)-N-(2,2,2-trifluoroacetyl)-L-threoninate (1.5 eq) and VB-B-31i (1 eq) in methanol was added TEA (2.5 eq) stirred at 55 °C for 48 h. Remove the solvent by rotavapor and work up with ethyl acetate. Purified by silics gel column chromatography. ^{1}H NMR (400 MHz, DMSO) δ 8.52 – 8.28 (m, 1H), 7.67 (s, 1H), 7.36 (d, *J* = 5.5 Hz, 1H), 7.09 (s, 1H), 4.95 (dd, *J* = 11.1, 4.1 Hz, 1H), 4.30 (ddd, *J* = 11.8, 8.2, 4.1 Hz, 1H), 3.29 – 3.01 (m, 3H),

2.35 (t, J = 10.8 Hz, 1H), 2.14 – 1.94 (m, 2H), 1.74 – 1.66 (m, 6H), 1.59 – 1.51 (m, 1H), 1.30 (s, 1H), 1.25 – 1.11 (m, 5H), 1.00 – 0.88 (m, 2H).

tert-Butyl (S)-7-(((S)-1-methoxy-1-oxo-3-((S)-2-oxopyrrolidin-3-yl)propan-2-yl)carbamoyl)-6-azaspiro[3.4]octane-6-carboxylate (YR-B-101e). YR-B-101e was prepared by **(S)-6-(tert-butoxycarbonyl)-6-azaspiro[3.4]octane-7-carboxylic acid** and **Int.i** using general procedure **C**. (Yield 79%). ^{1}H NMR (400 MHz, $CDCl_3$) δ 4.51 (d, J = 54.1 Hz, 1H), 4.22 (t, J = 6.7 Hz, 1H), 3.72 (s, 3H), 3.40 (dd, J = 15.8, 8.3 Hz, 4H), 2.43 (s, 2H), 2.15 (dd, J = 17.5, 7.7 Hz, 3H), 1.99 – 1.77 (m, 8H), 1.44 (s, 9H).

tert-Butyl (S)-7-(((S)-1-hydroxy-3-((S)-2-oxopyrrolidin-3-yl)propan-2-yl)carbamoyl)-6-azaspiro[3.4]octane-6-carboxylate (Yr-B-101f). MPI101f was prepared as a white solid following a general procedure **D** (Yield 62%). ^{1}H NMR (400 MHz, $CDCl_3$) δ 4.18 (ddd, J = 8.2, 5.6, 2.2 Hz, 1H), 4.02 (s, 1H), 3.80 – 3.23 (m, 6H), 2.37 (s, 2H), 2.24 – 1.73 (m, 11H), 1.44 (s, 9H).

Additional Supplementary NMR Spectra of Finally Synthesized Compounds

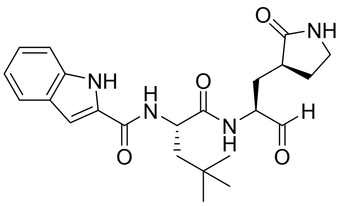
O
NH
NH
H
N
O
N
H
H
O
O

MPI48

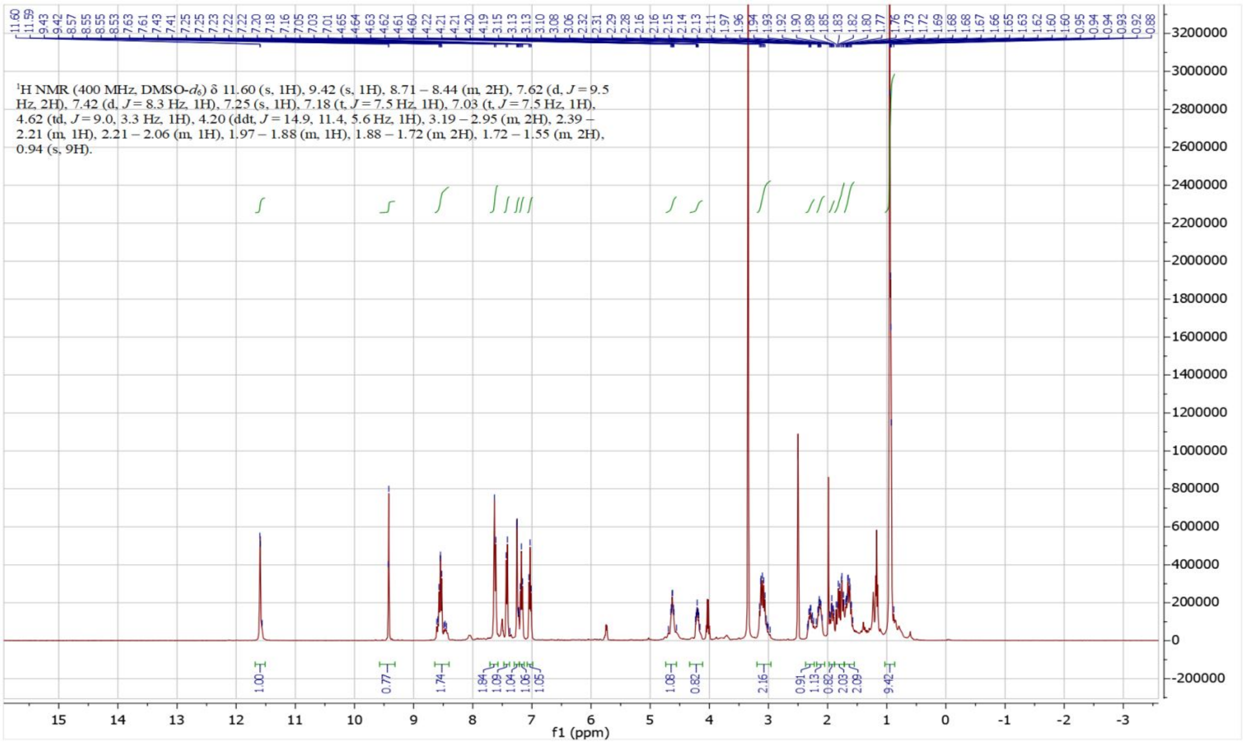
^{1}H NMR (400 MHz, DMSO-d_6) δ 11.60 (s, 1H), 9.42 (s, 1H), 8.71 – 8.44 (m, 2H), 7.62 (d, J = 9.5 Hz, 2H), 7.42 (d, J = 8.3 Hz, 1H), 7.25 (s, 1H), 7.18 (t, J = 7.5 Hz, 1H), 7.03 (t, J = 7.5 Hz, 1H), 4.62 (td, J = 9.0, 3.3 Hz, 1H), 4.20 (ddt, J = 14.9, 11.4, 5.6 Hz, 1H), 3.19 – 2.95 (m, 2H), 2.39 – 2.21 (m, 1H), 2.21 – 2.06 (m, 1H), 1.97 – 1.88 (m, 1H), 1.88 – 1.72 (m, 2H), 1.72 – 1.55 (m, 2H), 0.94 (s, 9H).
f1 (ppm)

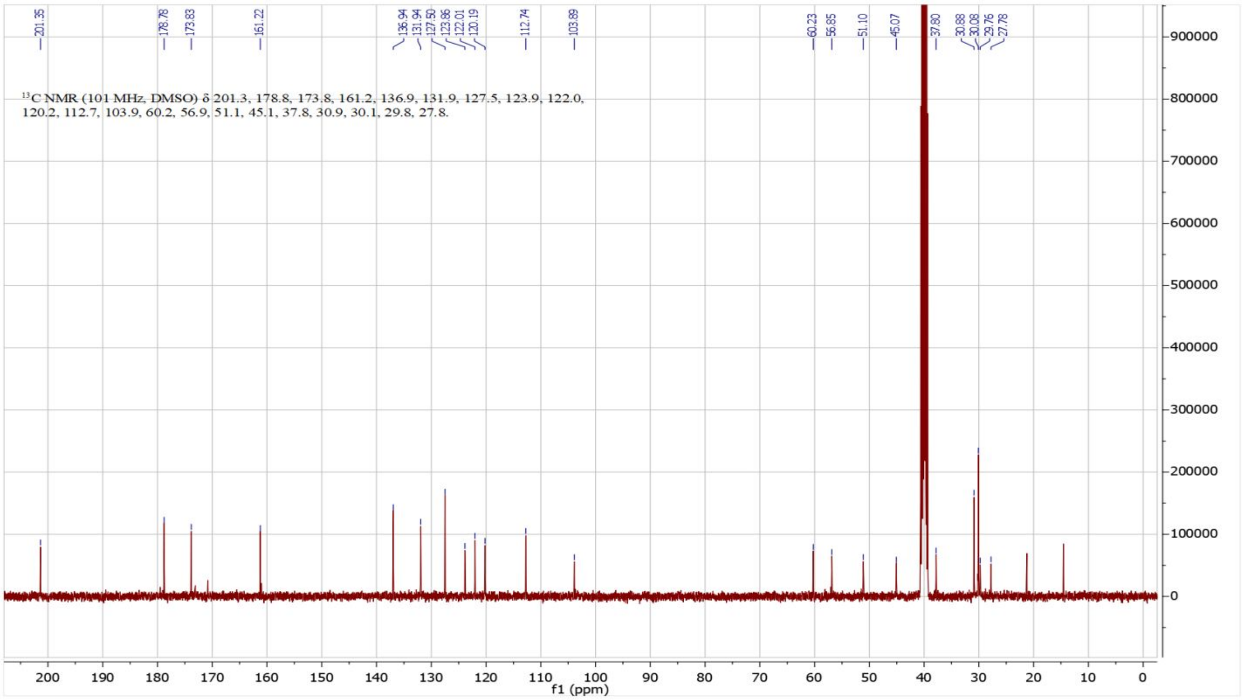
^{13}C NMR (101 MHz, DMSO) δ 201.3, 178.8, 173.8, 161.2, 136.9, 131.9, 127.5, 123.9, 122.0, 120.2, 112.7, 103.9, 60.2, 56.9, 51.1, 45.1, 37.8, 30.9, 30.1, 29.8, 27.8.
f1 (ppm)

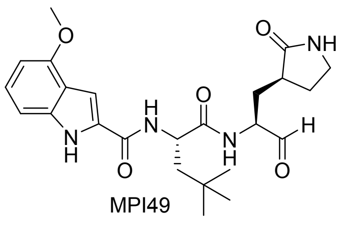

MPI49

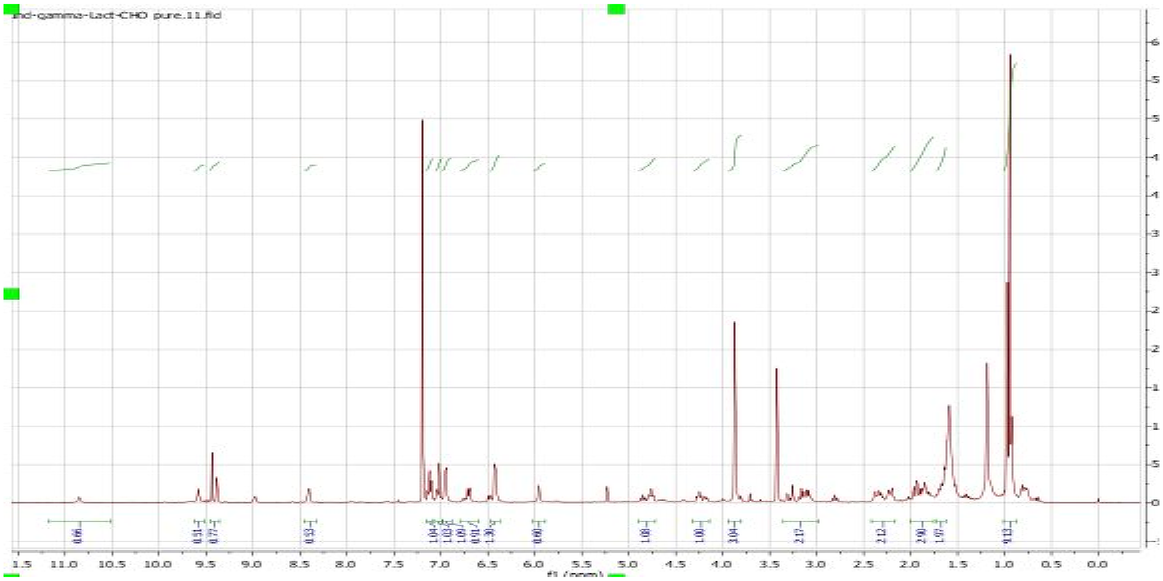
Ind-gamma-Lact-CHO pure.11.fid
f1 (ppm)

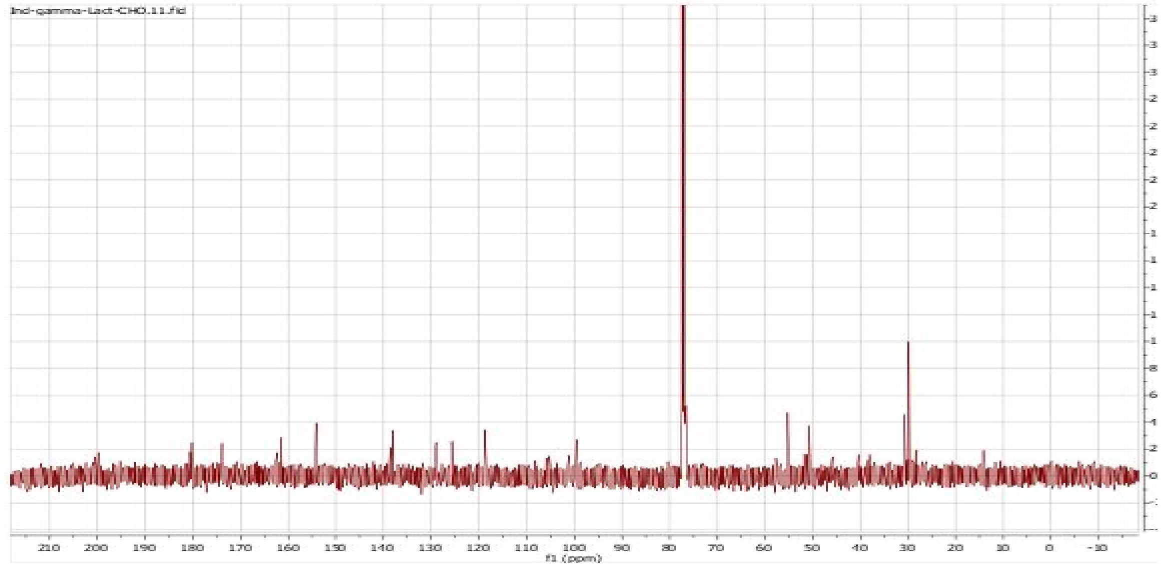
Ind-gamma-Lact-CHO.11.fid
f1 (ppm)

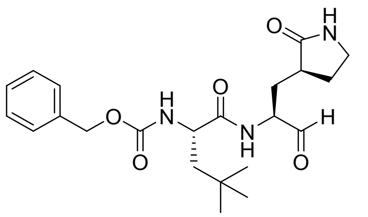

MPI50

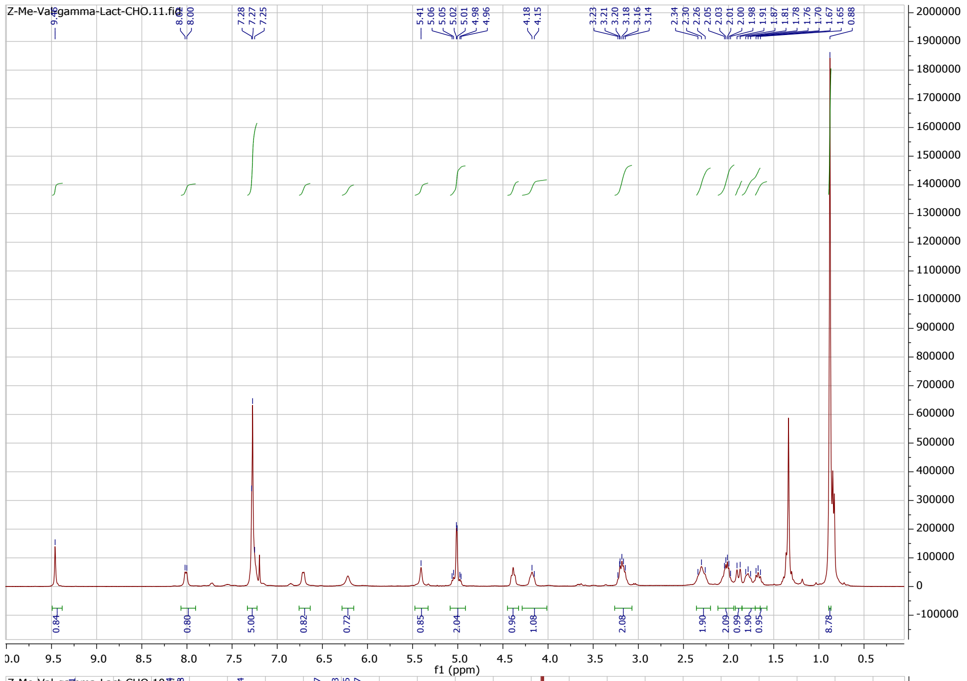
Z-Me-Val-gamma-Lact-CHO.11.fid
8.00
7.28
7.27
7.25
5.41
5.06
5.05
5.02
5.01
4.98
4.96
4.18
4.15
3.23
3.21
3.20
3.18
3.16
3.14
2.34
2.30
2.26
2.05
2.03
2.01
2.00
1.98
1.91
1.87
1.81
1.78
1.76
1.70
1.67
1.65
0.88
0.84
0.80
5.00
0.82
0.72
0.85
2.04
0.96
1.08
2.08
1.90
2.09
0.99
1.90
0.95
8.78
f1 (ppm)

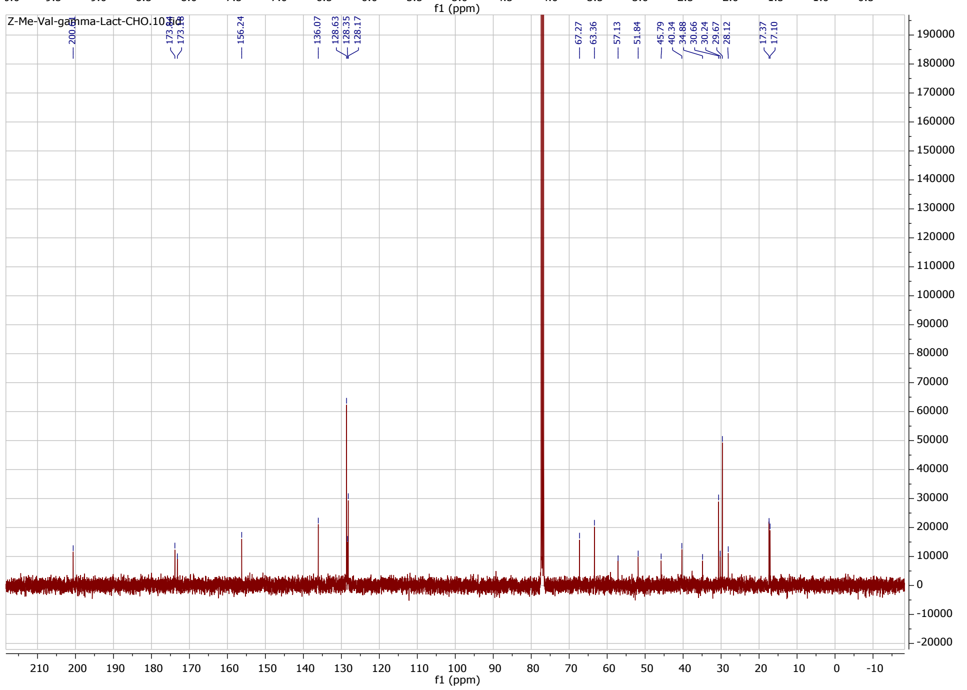
Z-Me-Val-gamma-Lact-CHO.10.fid
156.24
136.07
128.63
128.35
128.17
67.27
63.36
57.13
51.84
45.79
40.34
34.88
30.66
30.24
29.67
28.12
17.37
17.10
f1 (ppm)

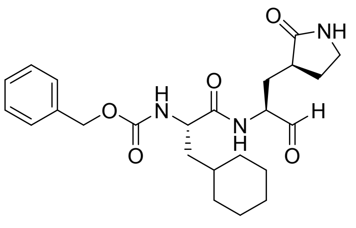
O
NH
H
N
O
O
N
H
H
O
O

MPI51

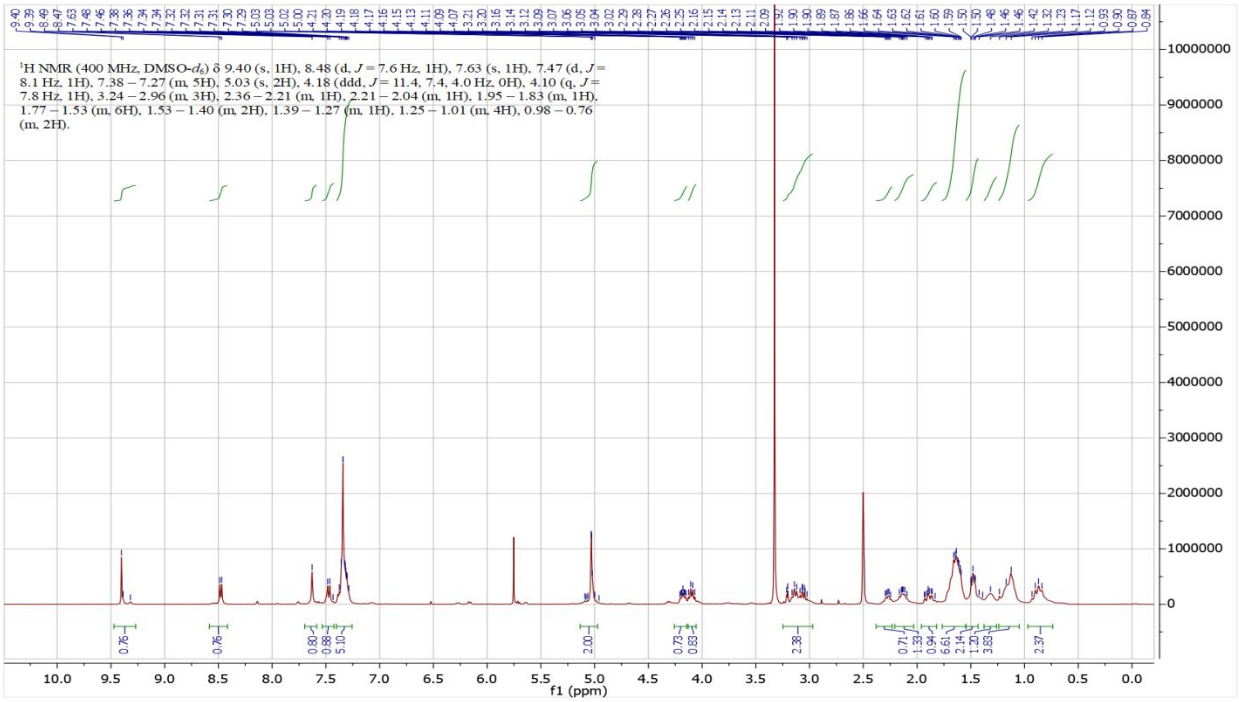
^{1}H NMR (400 MHz, DMSO-d_6) δ 9.40 (s, 1H), 8.48 (d, J = 7.6 Hz, 1H), 7.63 (s, 1H), 7.47 (d, J = 8.1 Hz, 1H), 7.38 – 7.27 (m, 5H), 5.03 (s, 2H), 4.18 (ddd, J = 11.4, 7.4, 4.0 Hz, 0H), 4.10 (q, J = 7.8 Hz, 1H), 3.24 – 2.96 (m, 3H), 2.36 – 2.21 (m, 1H), 2.21 – 2.04 (m, 1H), 1.95 – 1.83 (m, 1H), 1.77 – 1.53 (m, 6H), 1.53 – 1.40 (m, 2H), 1.39 – 1.27 (m, 1H), 1.25 – 1.01 (m, 4H), 0.98 – 0.76 (m, 2H).
f1 (ppm)

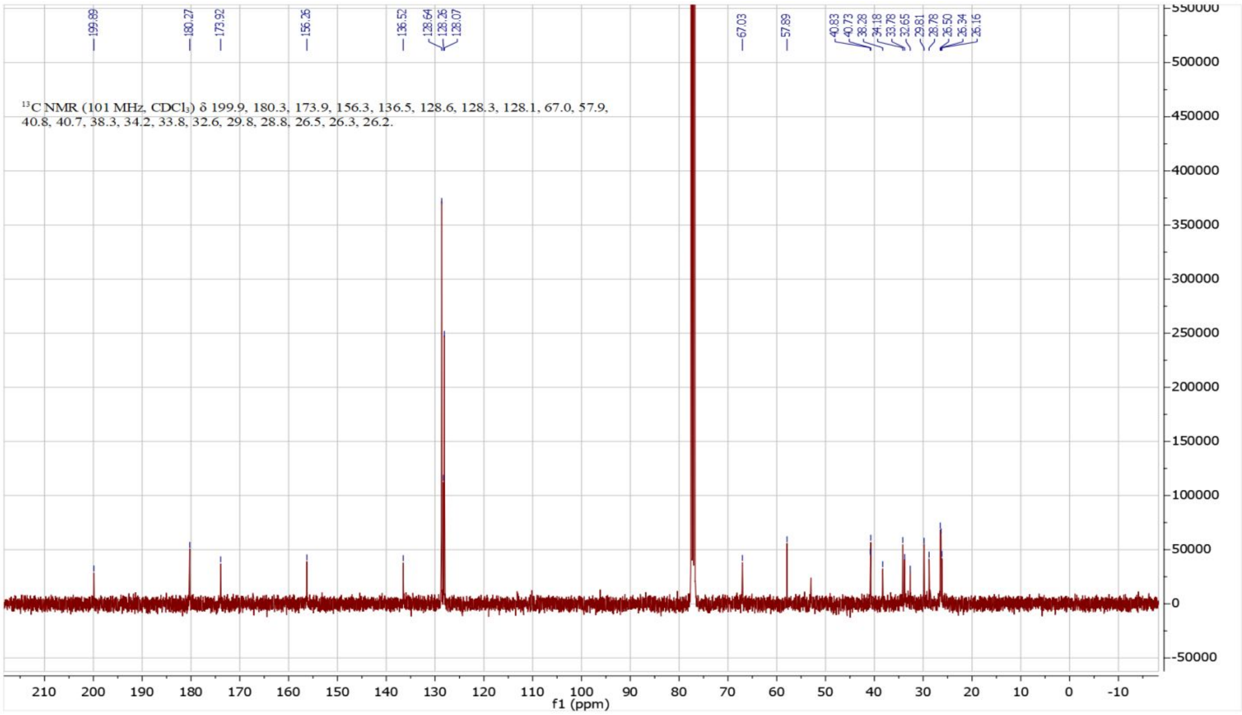
^{13}C NMR (101 MHz, $CDCl_3$) δ 199.9, 180.3, 173.9, 156.3, 136.5, 128.6, 128.3, 128.1, 67.0, 57.9, 40.8, 40.7, 38.3, 34.2, 33.8, 32.6, 29.8, 28.8, 26.5, 26.3, 26.2.
f1 (ppm)

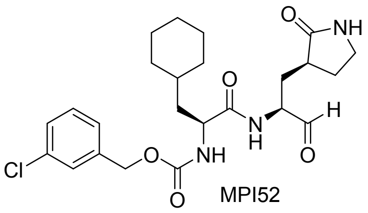
O
NH
O
N
H
H
O
Cl
O
NH
O

MPI52

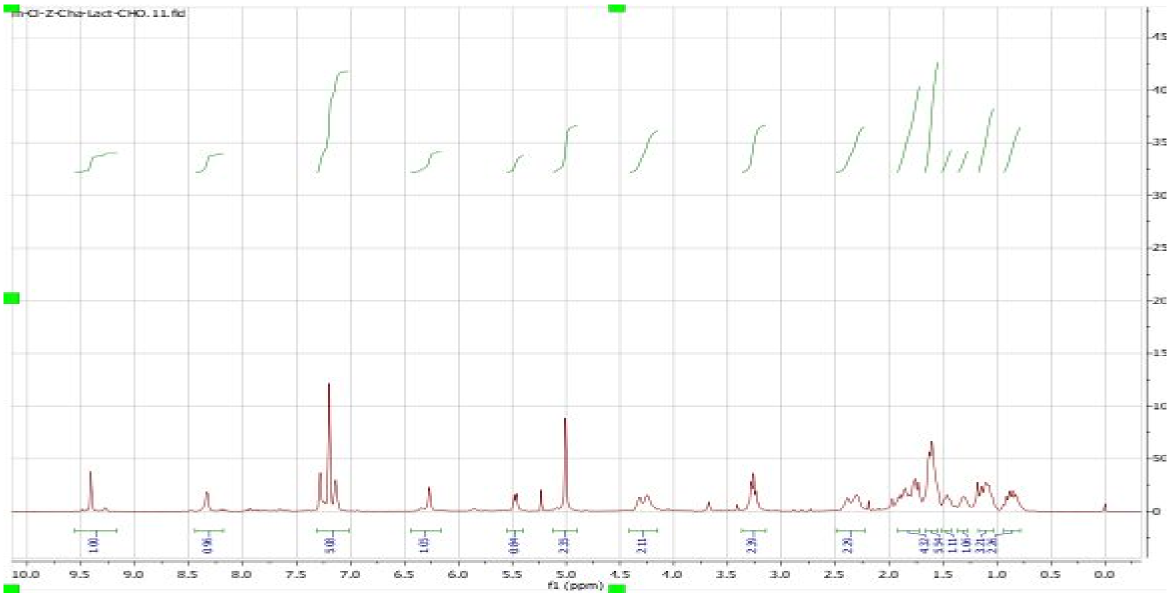
m-Cl-Z-Cha-Lact-CHO.11.fid
f1 (ppm)

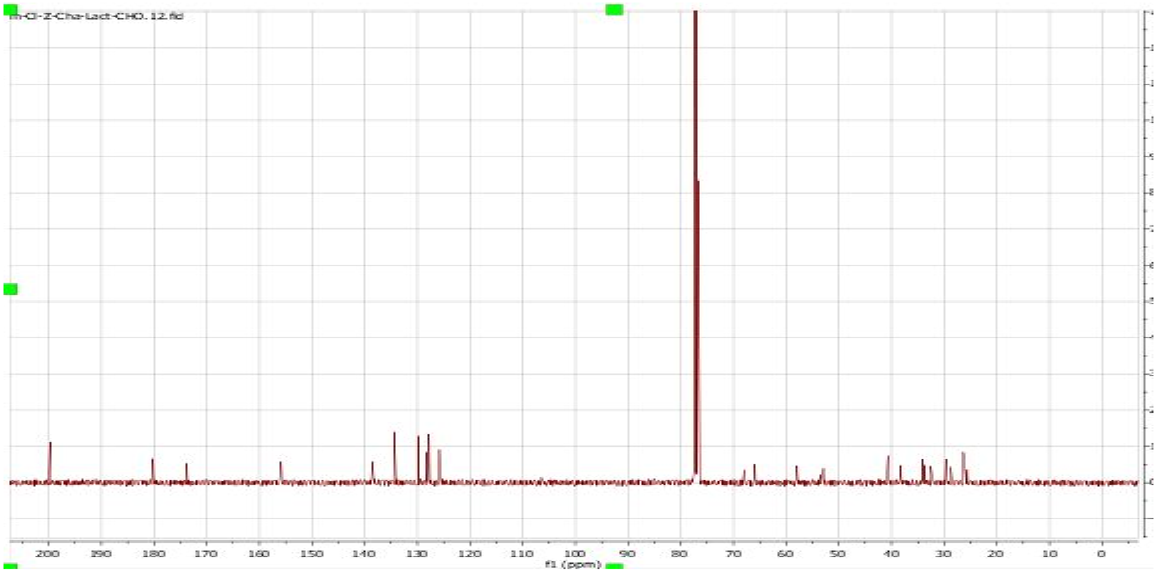
m-Cl-Z-Cha-Lact-CHO.12.fid
f1 (ppm)

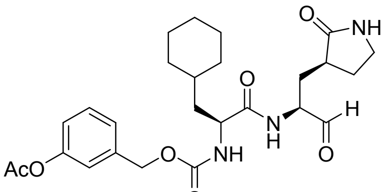

MPI53

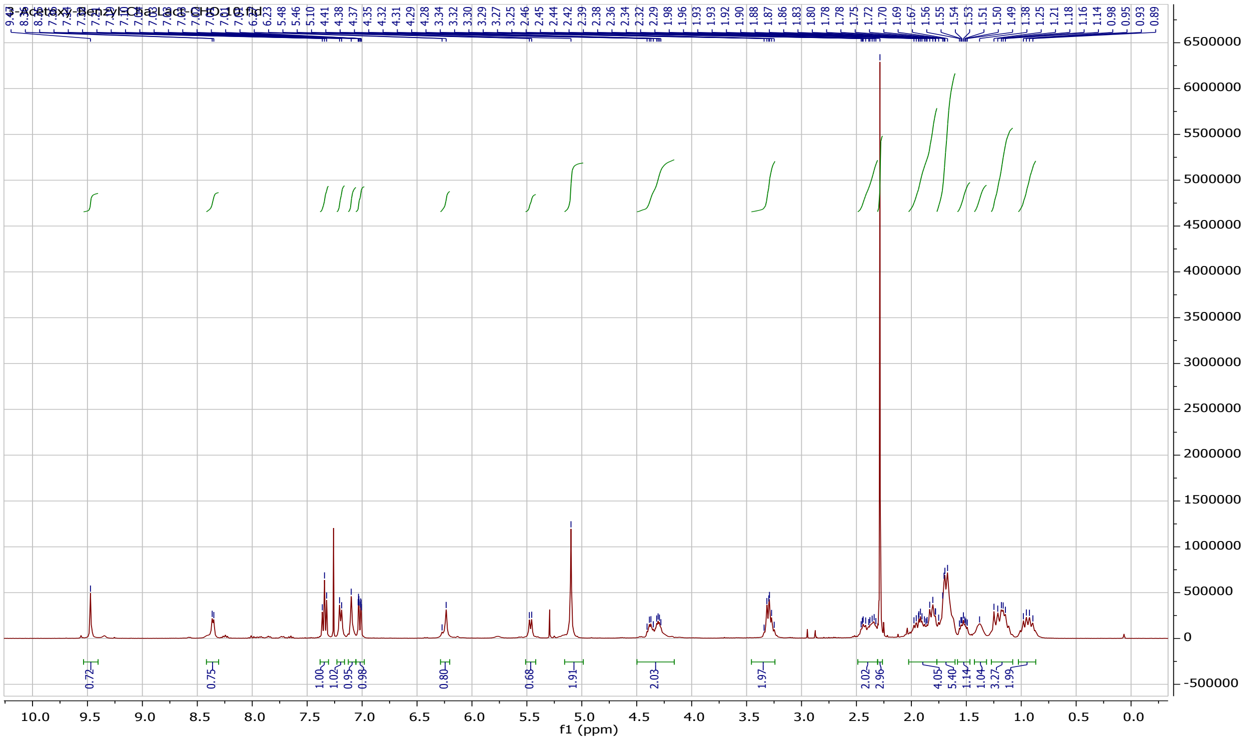

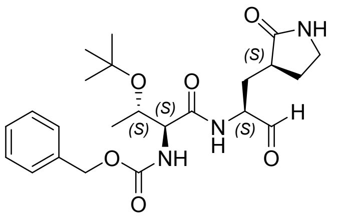
O
NH
(S)
O
(S)
(S)
O
H
N
(S)
H
O
NH
O
O

MPI54

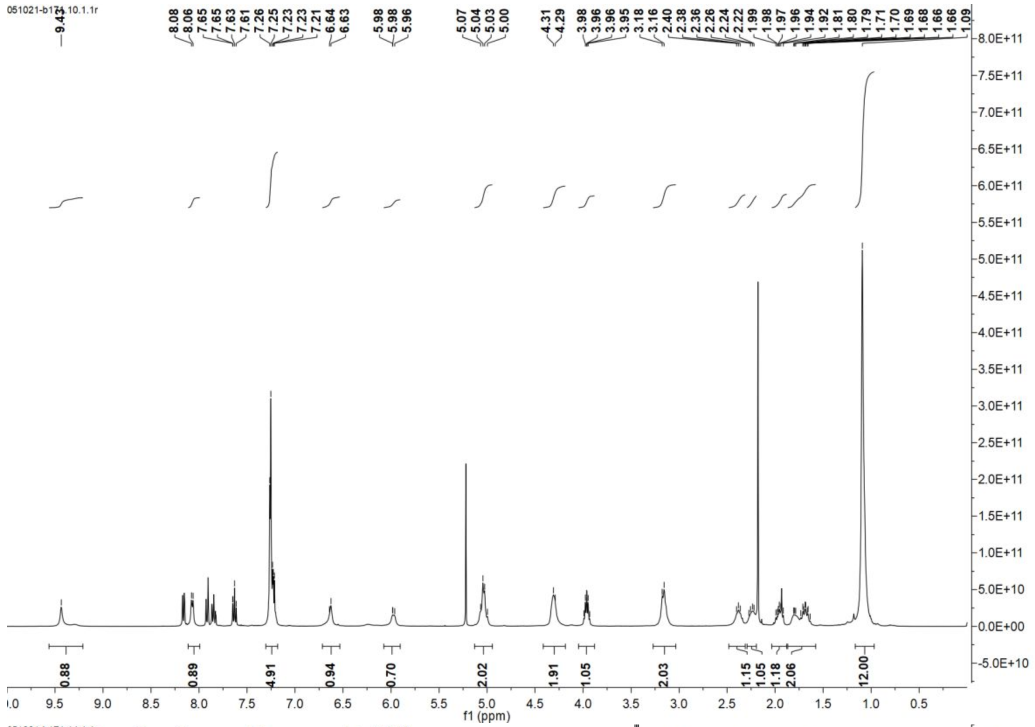
051021-b1[illegible].10.1.1r
9.43
8.08
8.06
7.65
7.63
7.61
7.26
7.25
7.23
7.23
7.21
6.64
6.63
5.98
5.98
5.96
5.07
5.04
5.03
5.00
4.31
4.29
3.98
3.96
3.96
3.95
3.18
3.16
2.40
2.38
2.36
2.26
2.24
2.22
1.99
1.98
1.97
1.96
1.94
1.92
1.81
1.80
1.79
1.71
1.70
1.69
1.68
1.66
1.09
0.88
0.89
4.91
0.94
0.70
2.02
1.91
1.05
2.03
1.15
1.05
1.18
2.06
12.00
f1 (ppm)

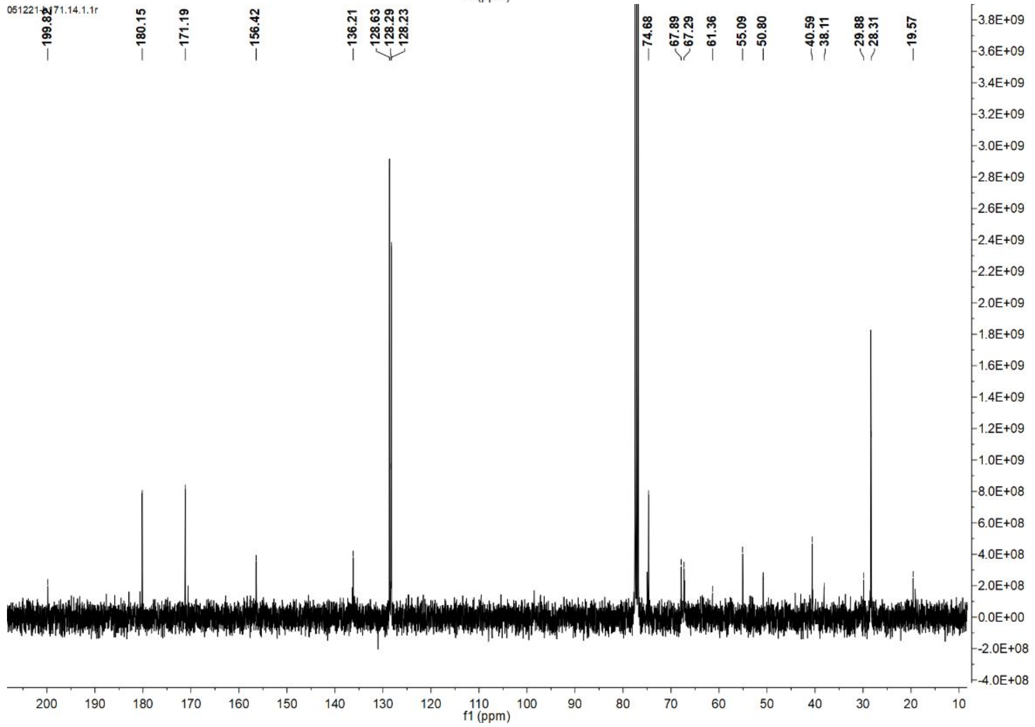
051221-b171.14.1.1r
199.82
180.15
171.19
156.42
136.21
128.63
128.29
128.23
74.68
67.89
67.29
61.36
55.09
50.80
40.59
38.11
29.88
28.31
19.57
f1 (ppm)

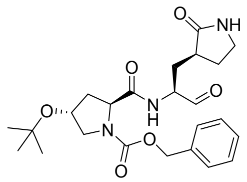

MPI55

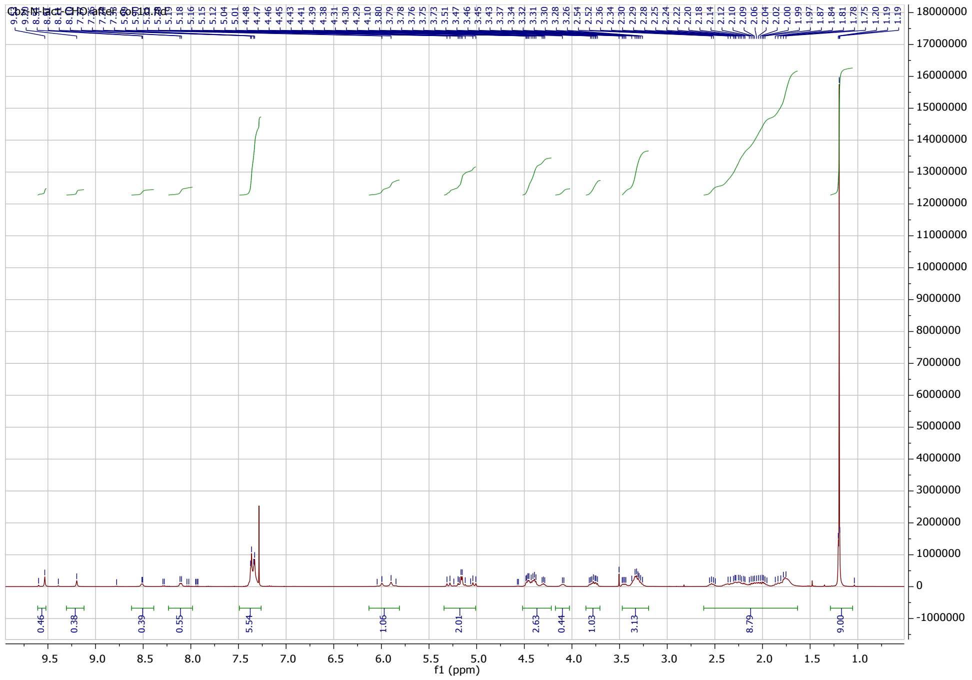
f1 (ppm)

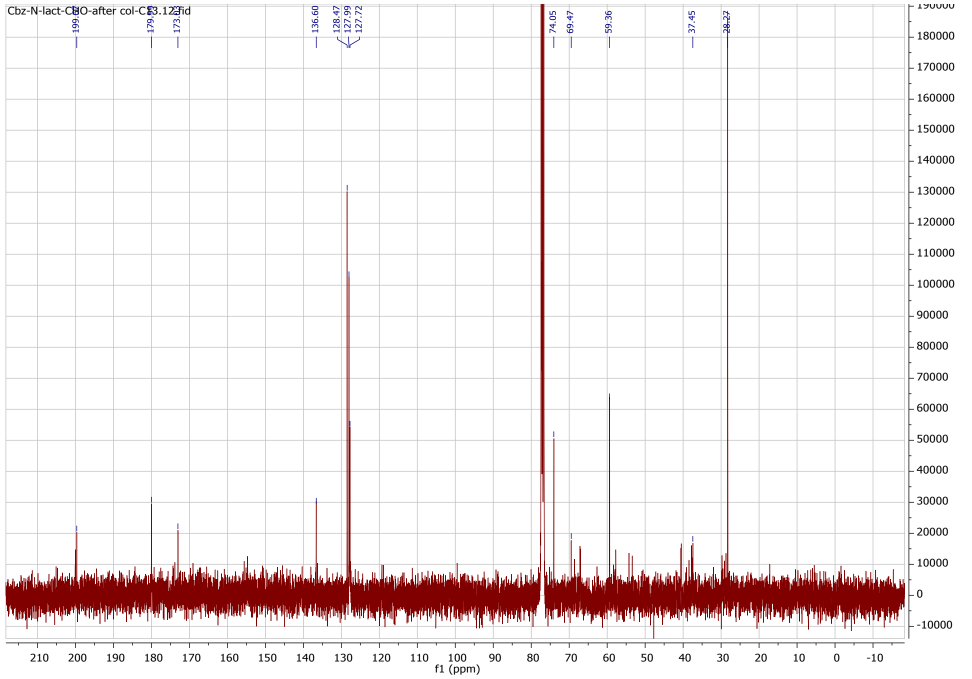
136.60
128.47
127.99
127.72
74.05
69.47
59.36
37.45
28.27
f1 (ppm)

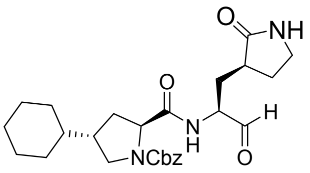
O
NH
O
N
H
H
NCbz
O

MPI56

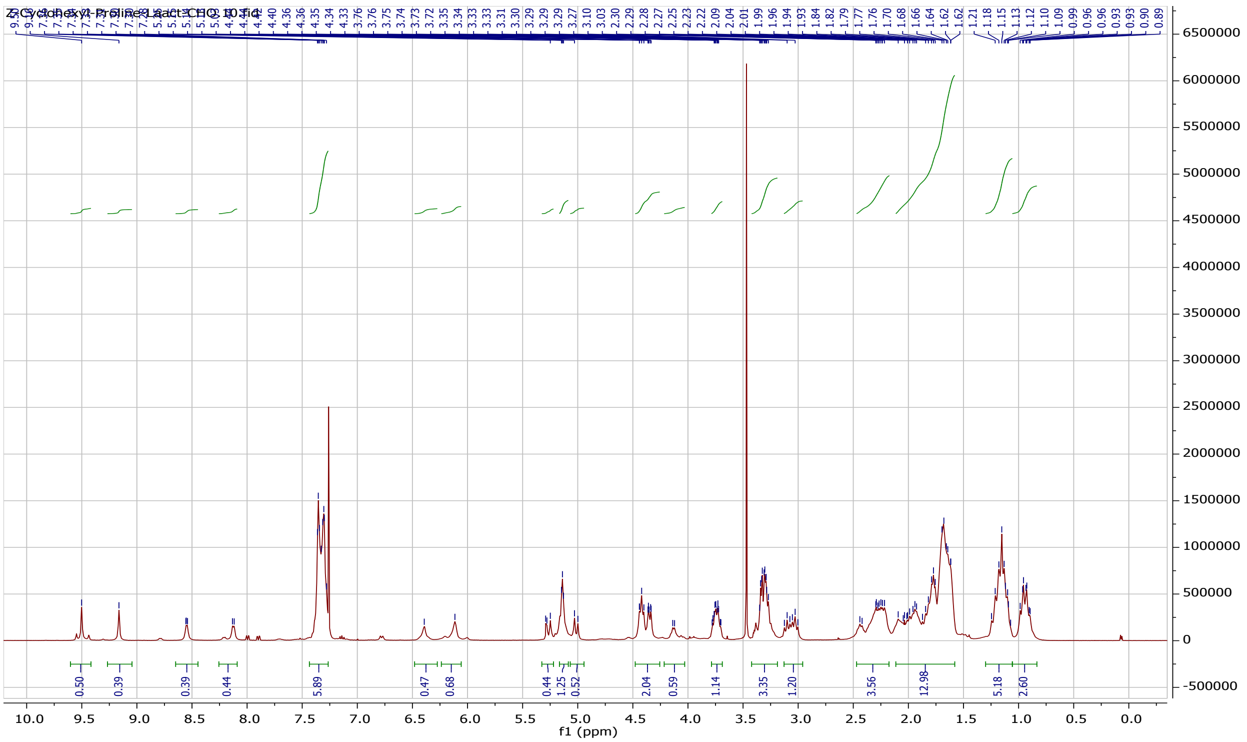
Z-Cyclohexyl-Proline-Laact-CHO.10.fid
0.50
0.39
0.39
0.44
5.89
0.47
0.68
0.44
1.25
0.52
2.04
0.59
1.14
3.35
1.20
3.56
12.98
5.18
2.60
f1 (ppm)

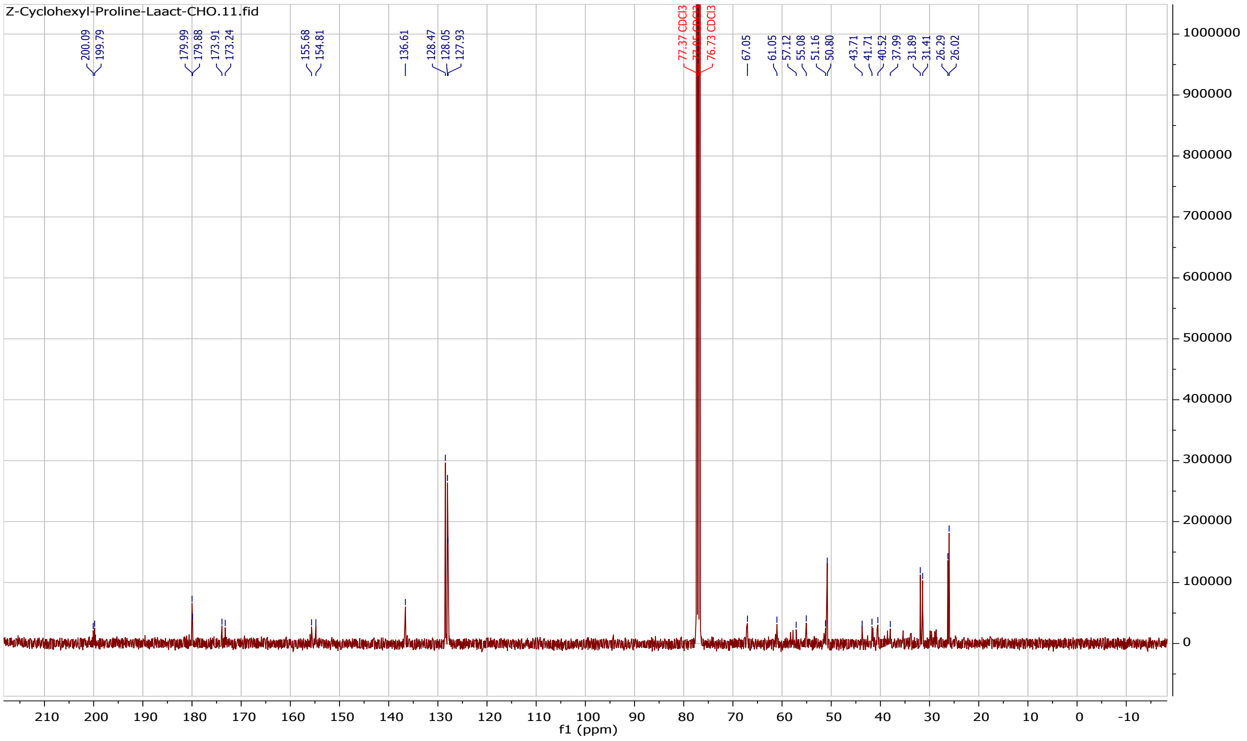
Z-Cyclohexyl-Proline-Laact-CHO.11.fid
200.09
199.79
179.99
179.88
173.91
173.24
155.68
154.81
136.61
128.47
128.05
127.93
77.37 CDCl3
76.73 CDCl3
67.05
61.05
57.12
55.08
51.16
50.80
43.71
41.71
40.52
37.99
31.89
31.41
26.29
26.02
f1 (ppm)

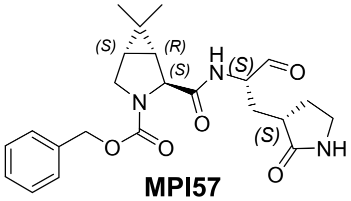

MPI57

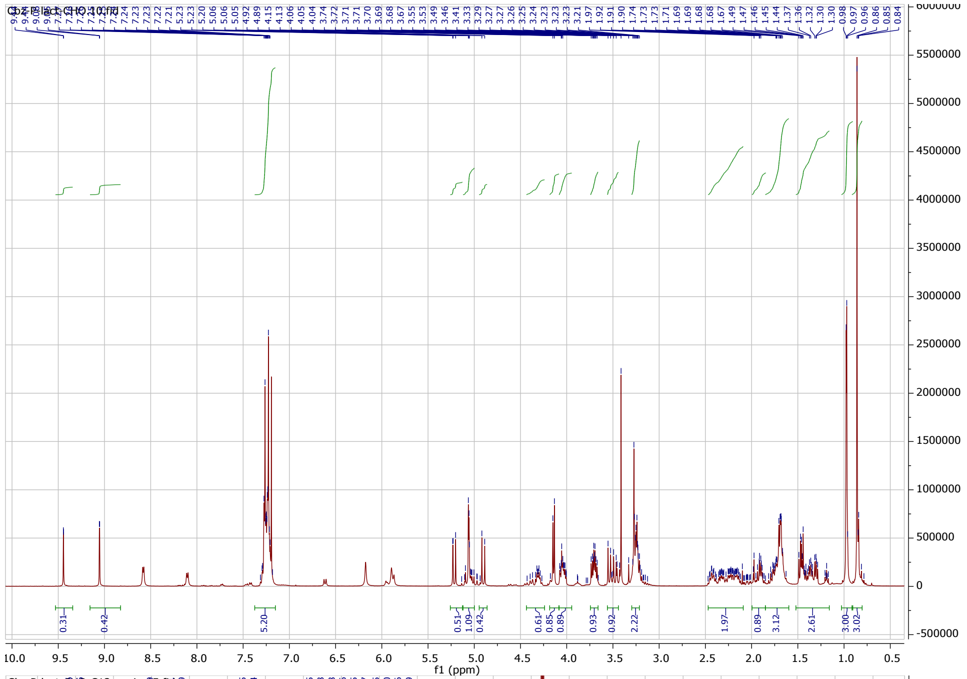

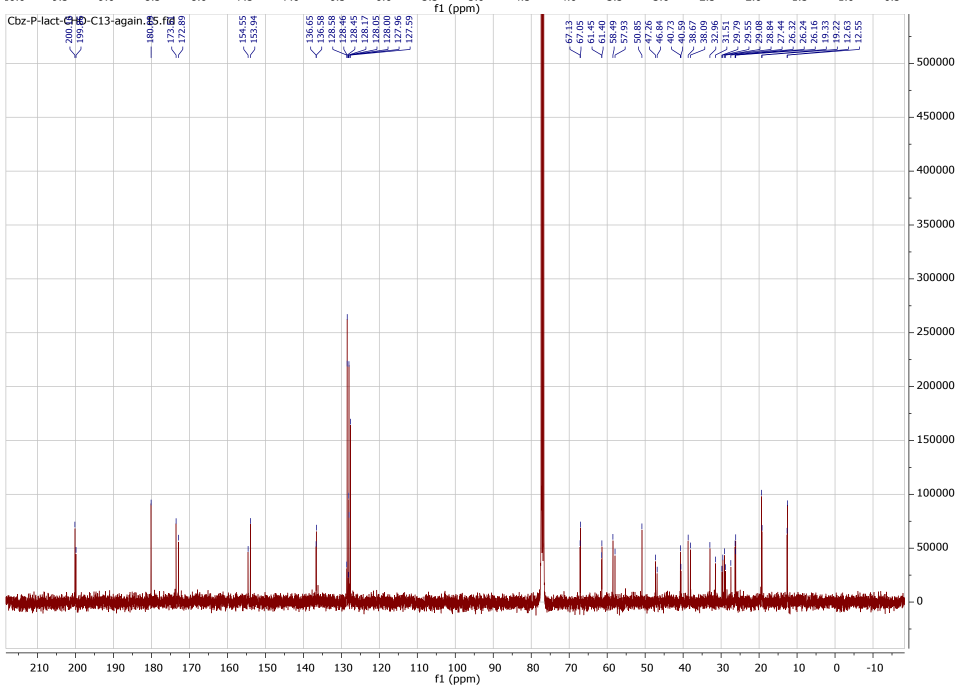

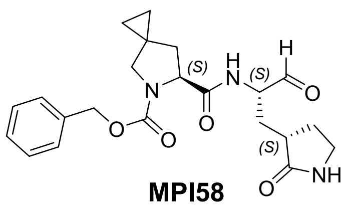

MPI58

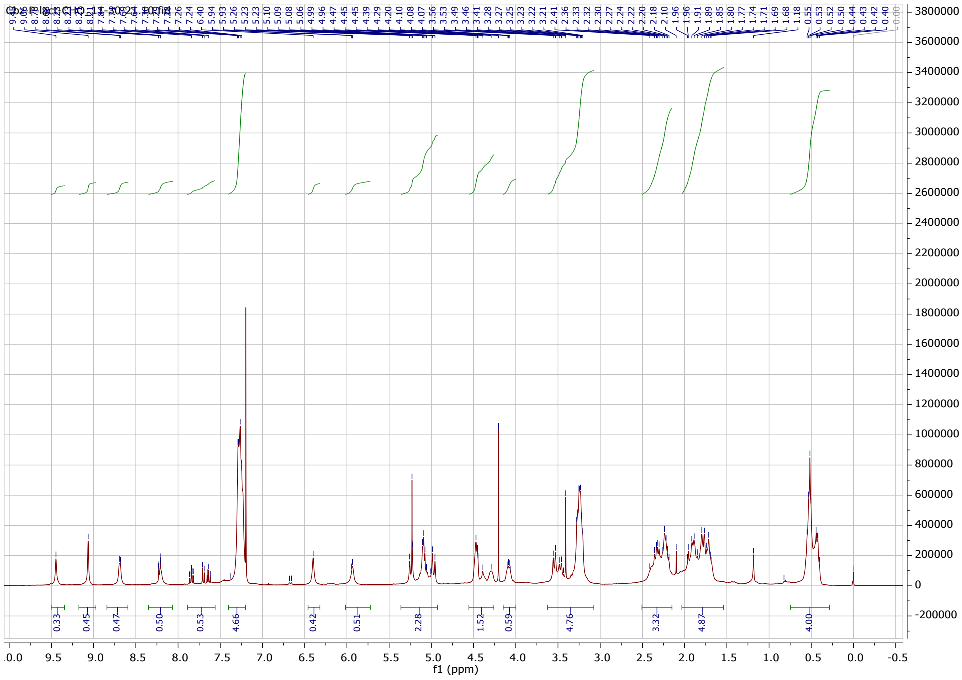

f1 (ppm)

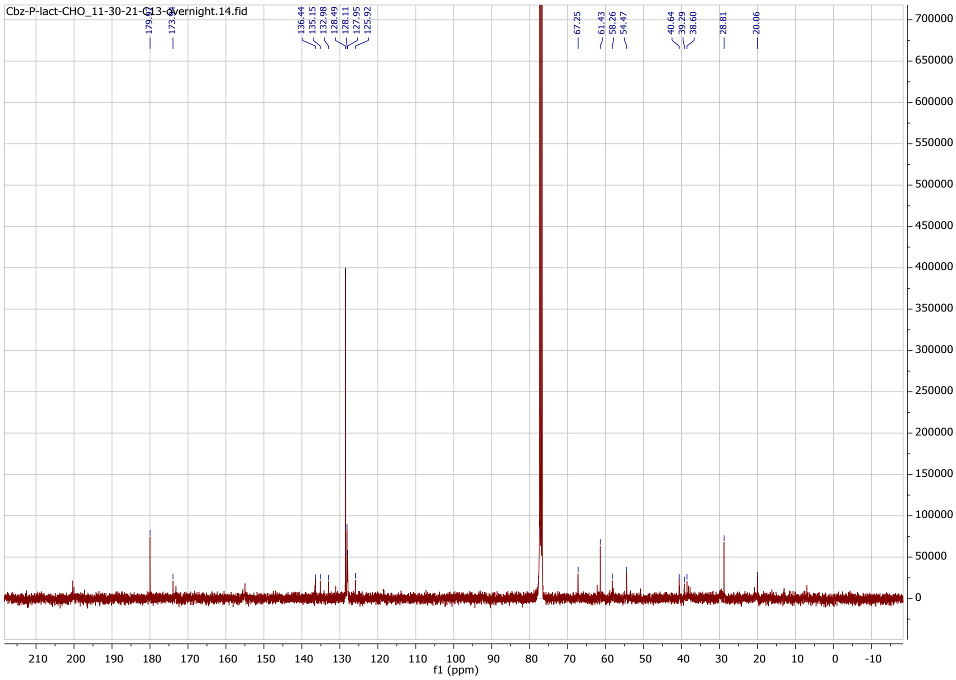

Cbz-P-lact-CHO_11-30-21-C13-overnight.14.fid
136.44
135.15
132.98
128.49
128.11
127.95
125.92
67.25
61.43
58.26
54.47
40.64
39.29
38.60
28.81
20.06
f1 (ppm)

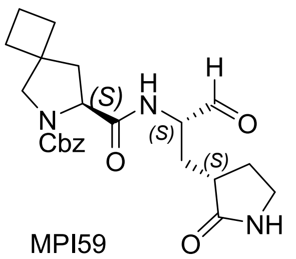

MPI59

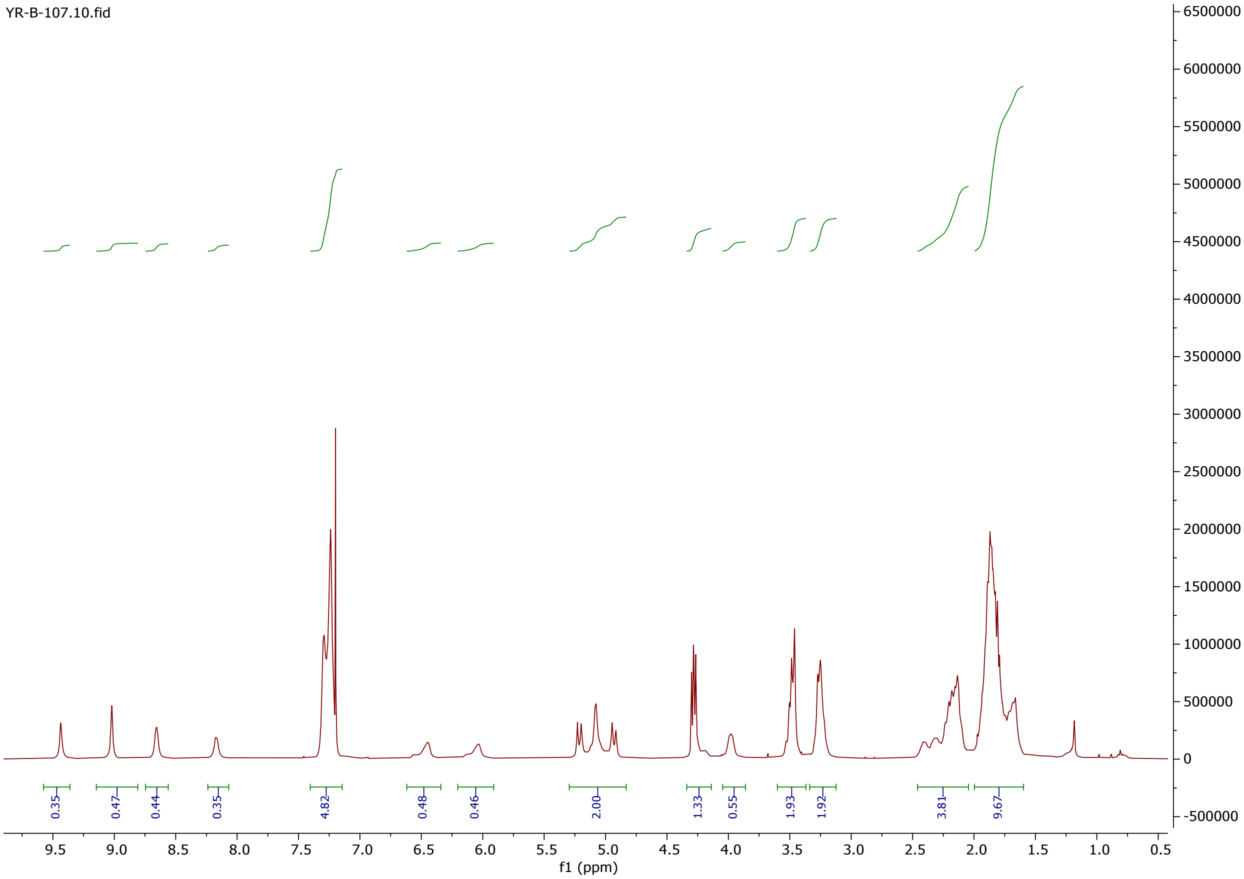
YR-B-107.10.fid
0.35
0.47
0.44
0.35
4.82
0.48
0.46
2.00
1.33
0.55
1.93
1.92
3.81
9.67
f1 (ppm)

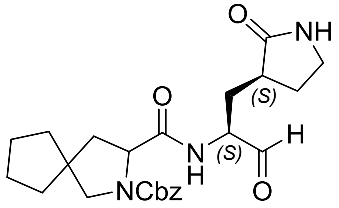
NH
(S)
(S)
NCbz
H

MPI60

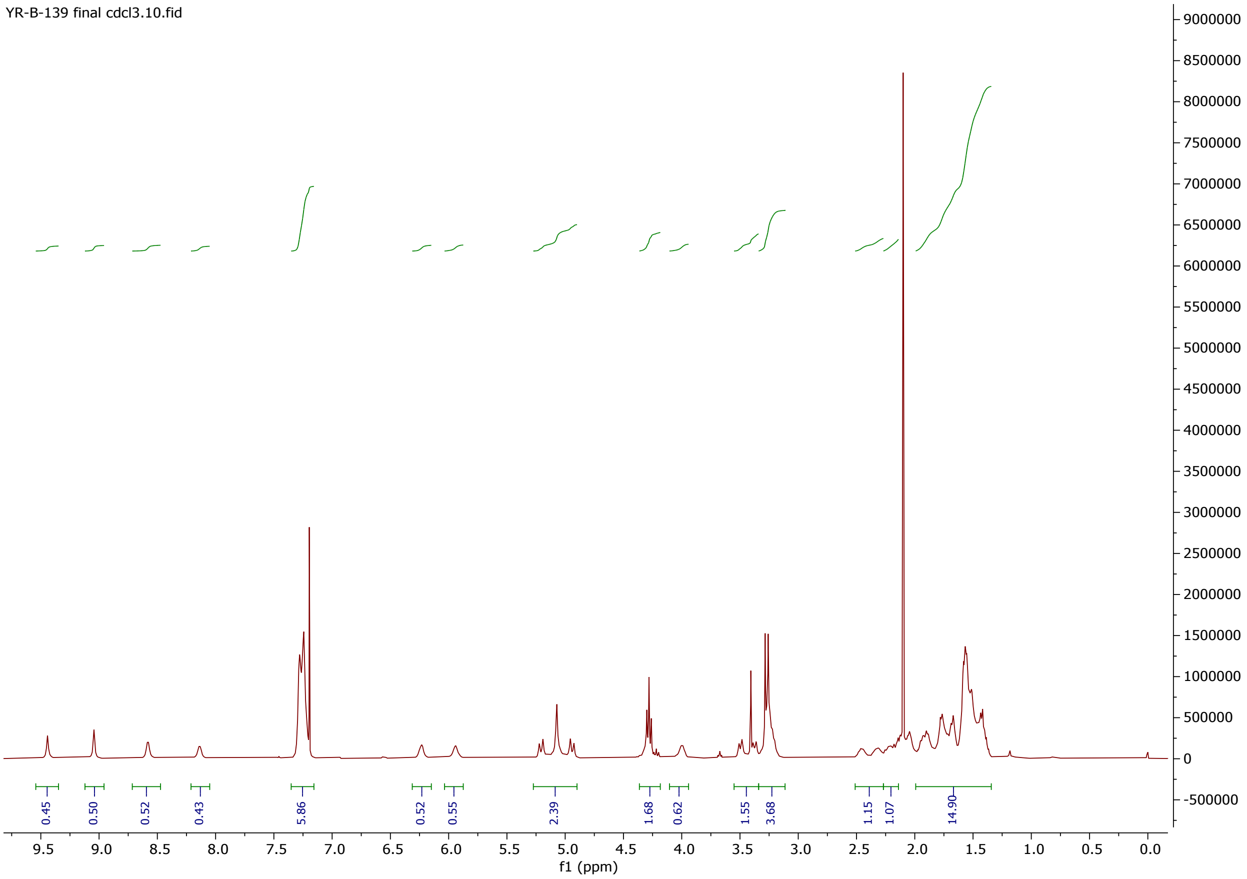
YR-B-139 final cdcl3.10.fid
0.45
0.50
0.52
0.43
5.86
0.52
0.55
2.39
1.68
0.62
1.55
3.68
1.15
1.07
14.90
f1 (ppm)

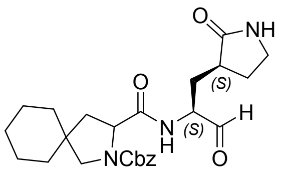
O
NH
(S)
O
H
N
H
(S)
NCbz
O

MPI61

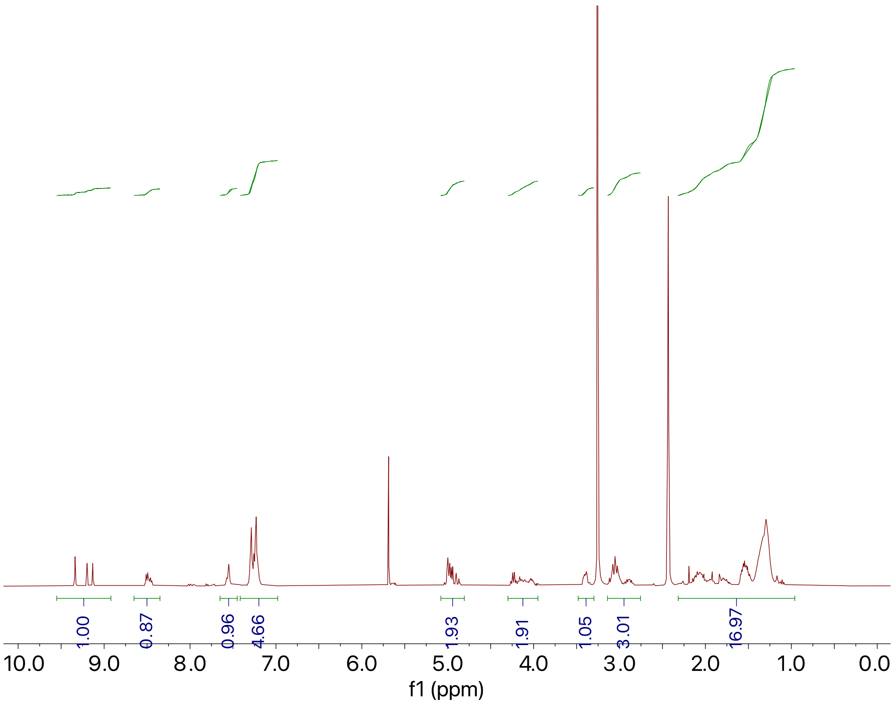
1.00
0.87
0.96
4.66
1.93
1.91
1.05
3.01
16.97
10.0 9.0 8.0 7.0 6.0 5.0 4.0 3.0 2.0 1.0 0.0
f1 (ppm)

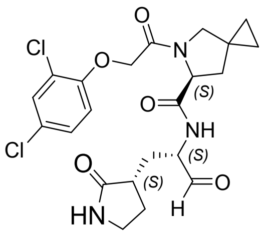

MPI63

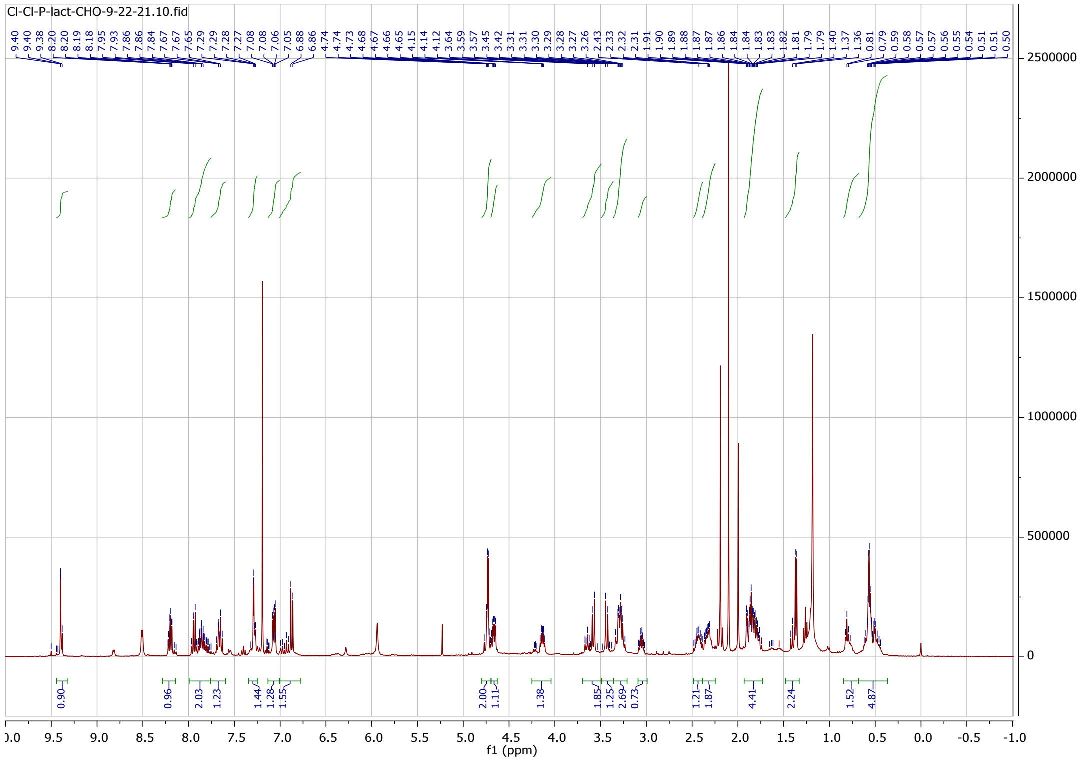

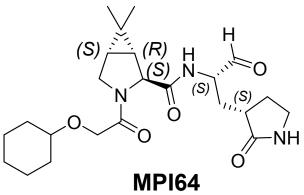
(S)
(R)
(S)
H
N
(S)
(S)
H
O
N
O
O
O
O
NH

MPI64

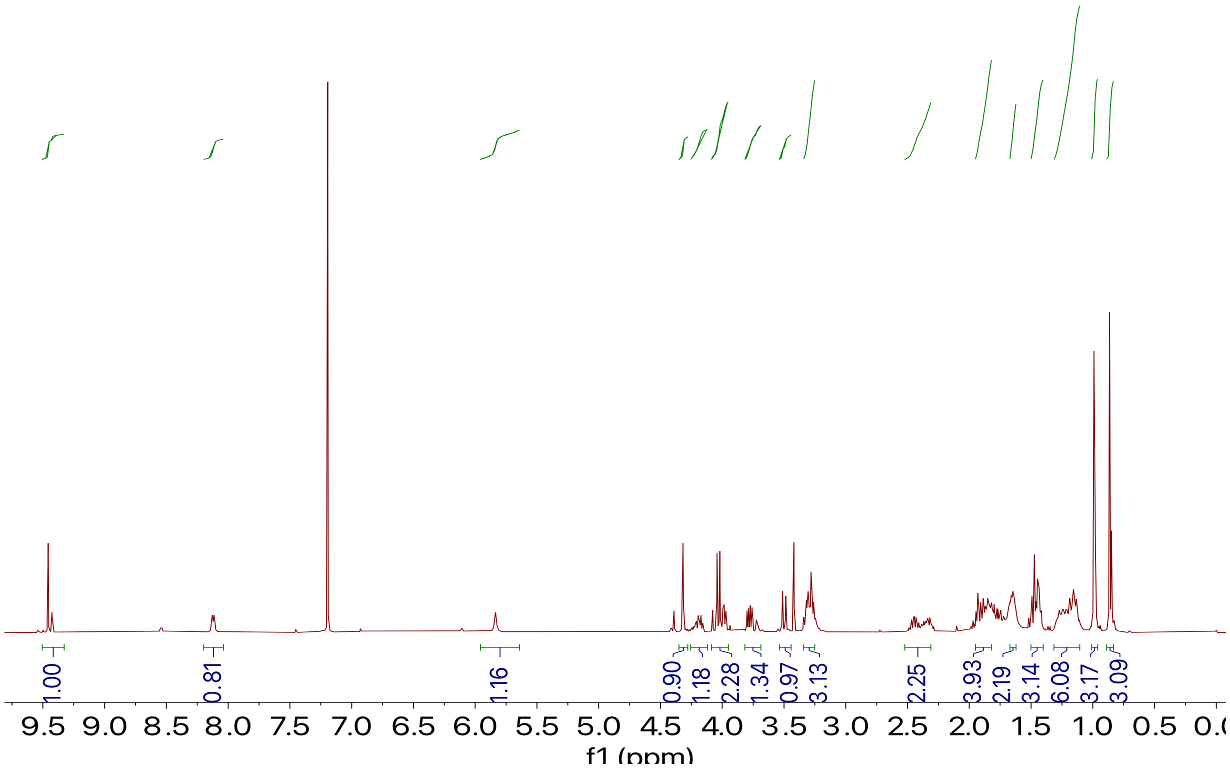
1.00
0.81
1.16
0.90
1.18
2.28
1.34
0.97
3.13
2.25
3.93
2.19
3.14
6.08
3.17
3.09
9.5 9.0 8.5 8.0 7.5 7.0 6.5 6.0 5.5 5.0 4.5 4.0 3.5 3.0 2.5 2.0 1.5 1.0 0.5
f1 (ppm)

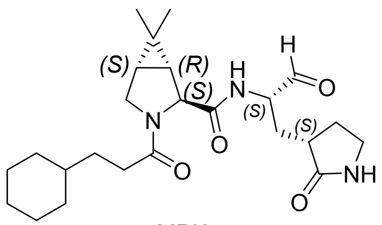

MPI65

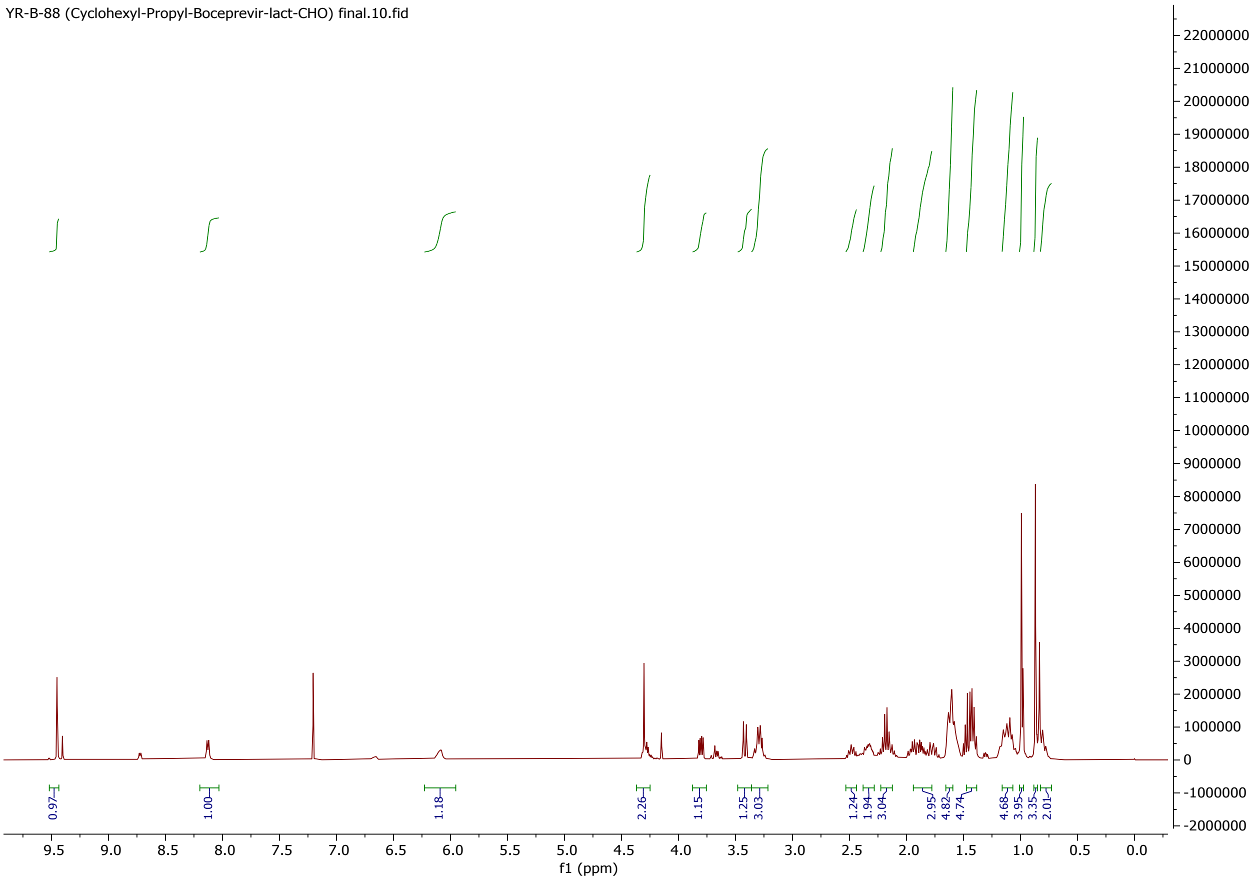

YR-B-88 (Cyclohexyl-Propyl-Boceprevir-lact-CHO) final.10.fid
f1 (ppm)

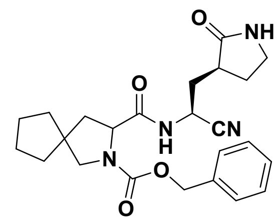
O
NH
O
N
H
CN
N
O
O

MPI66

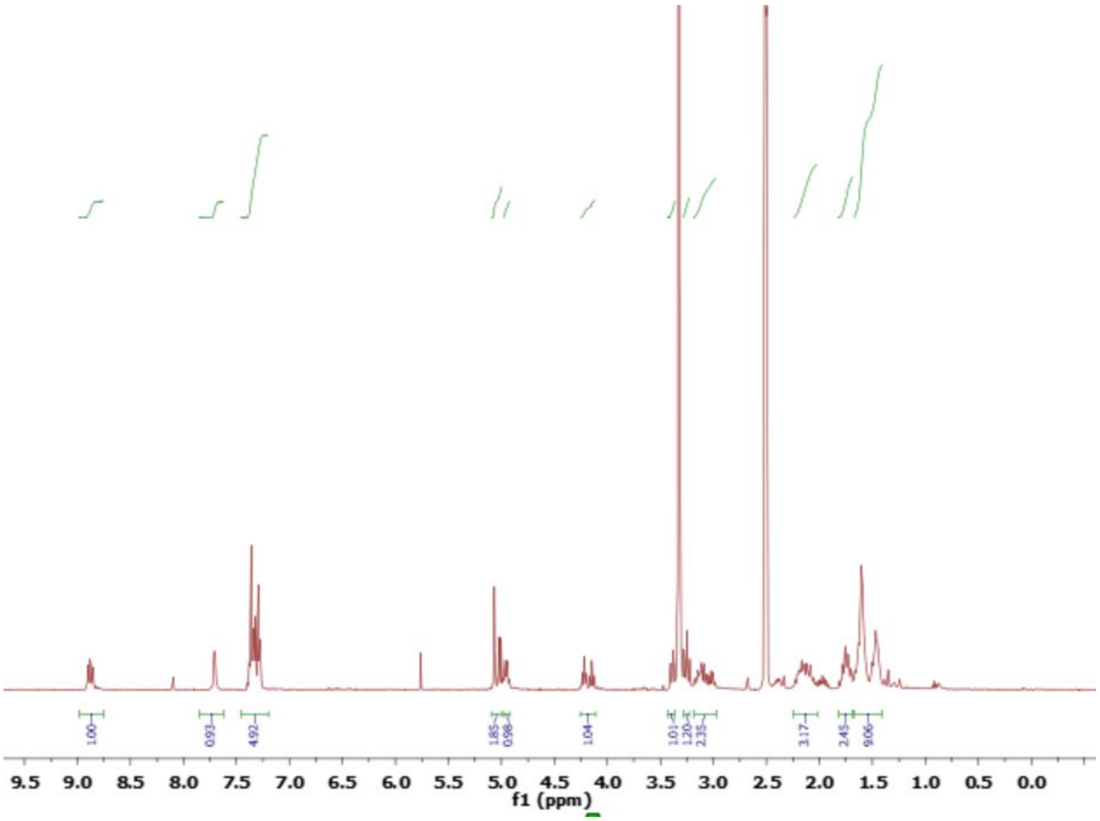
1.00
0.93
4.92
1.85
0.98
1.04
1.01
1.20
2.35
3.17
2.45
9.06
9.5 9.0 8.5 8.0 7.5 7.0 6.5 6.0 5.5 5.0 4.5 4.0 3.5 3.0 2.5 2.0 1.5 1.0 0.5 0.0
f1 (ppm)

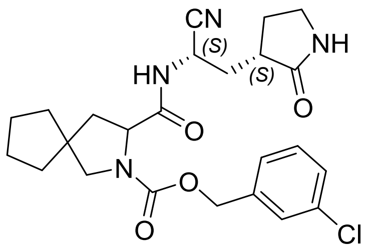

MPI66-2

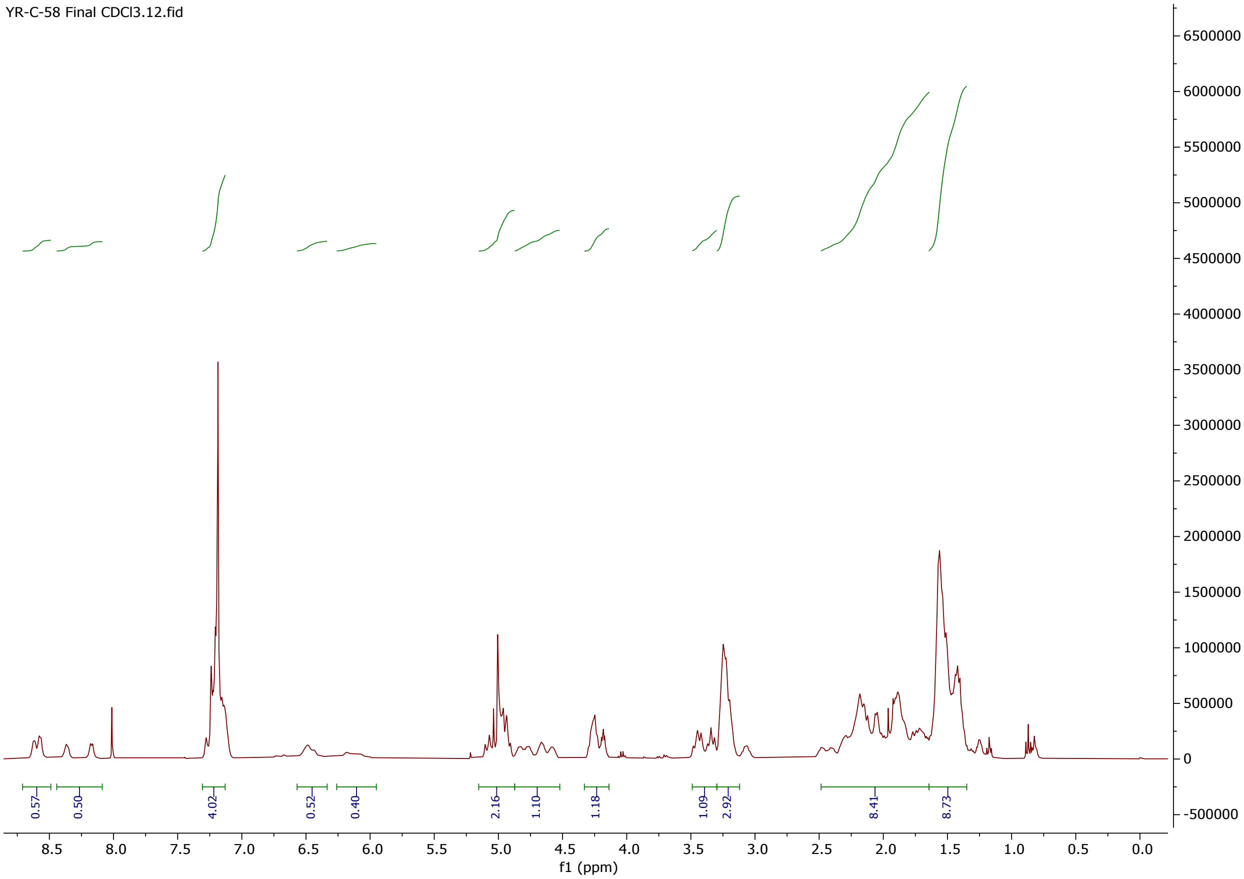

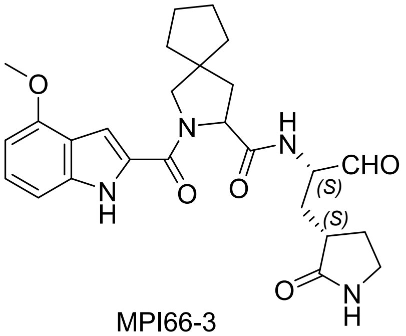

MPI66-3

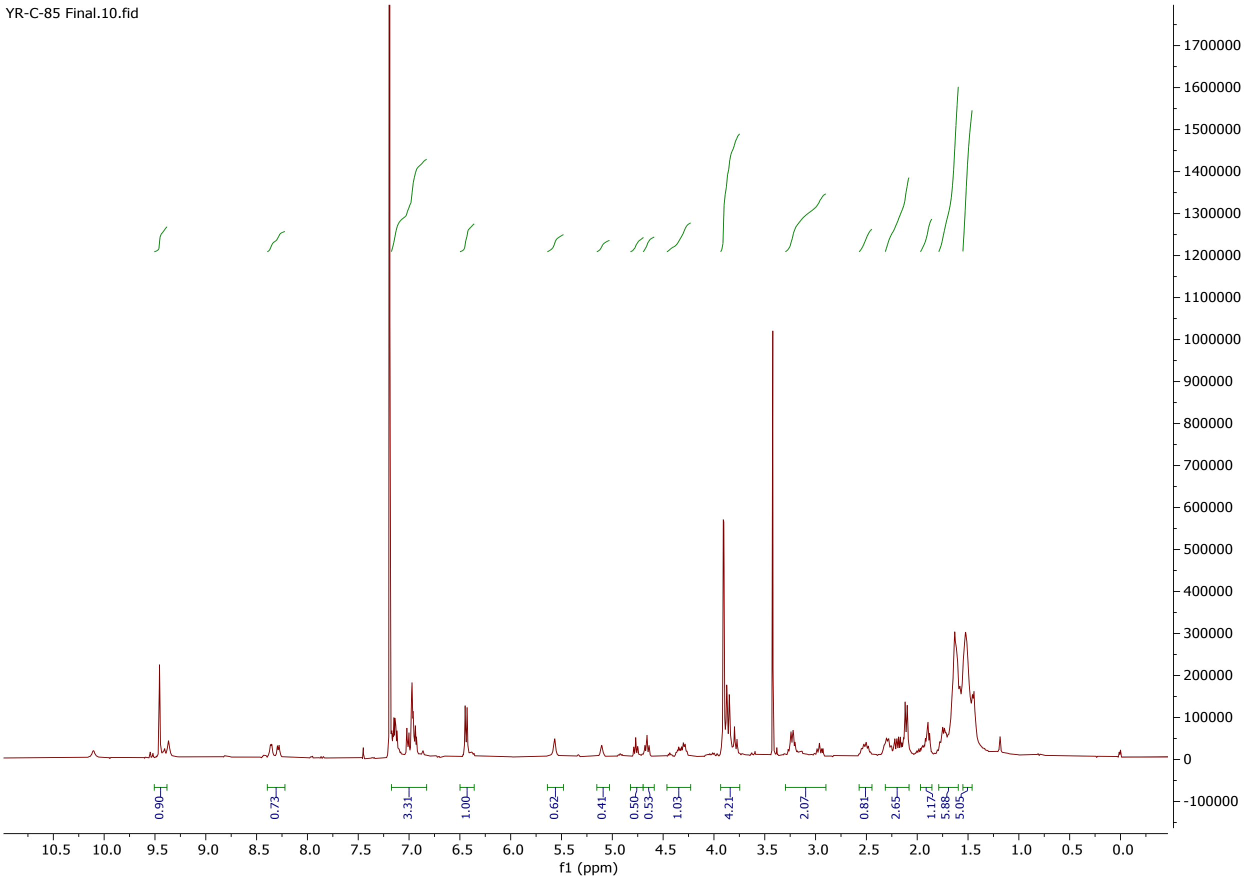

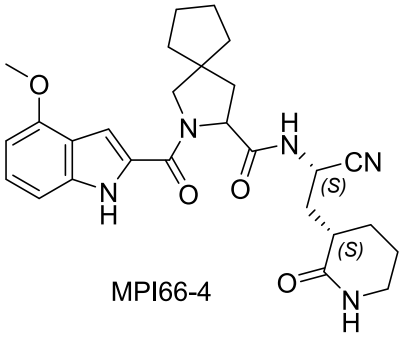

MPI66-4

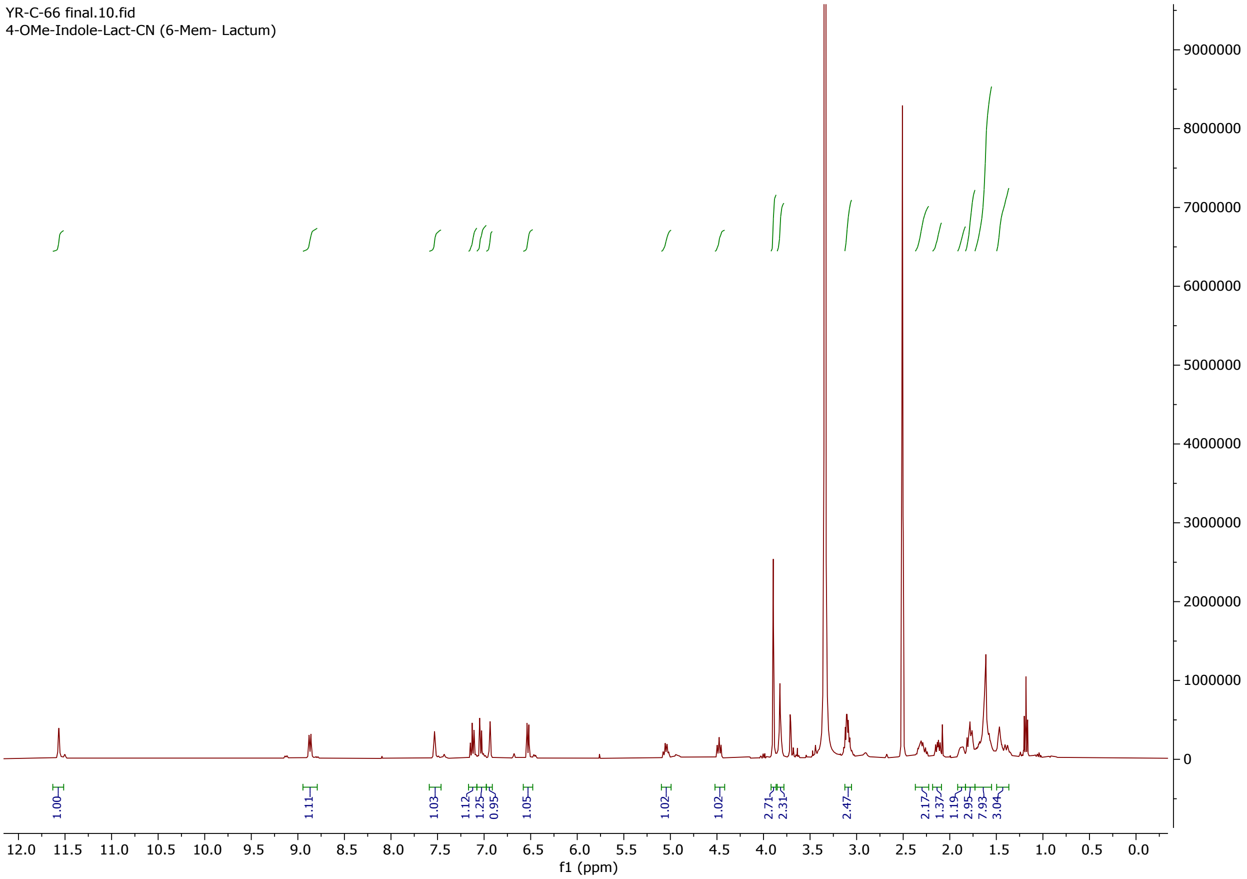

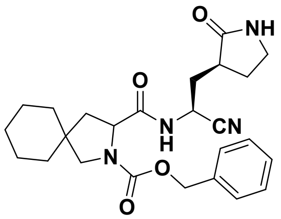
O
NH
O
N
H
CN
N
O
O
O

MPI67

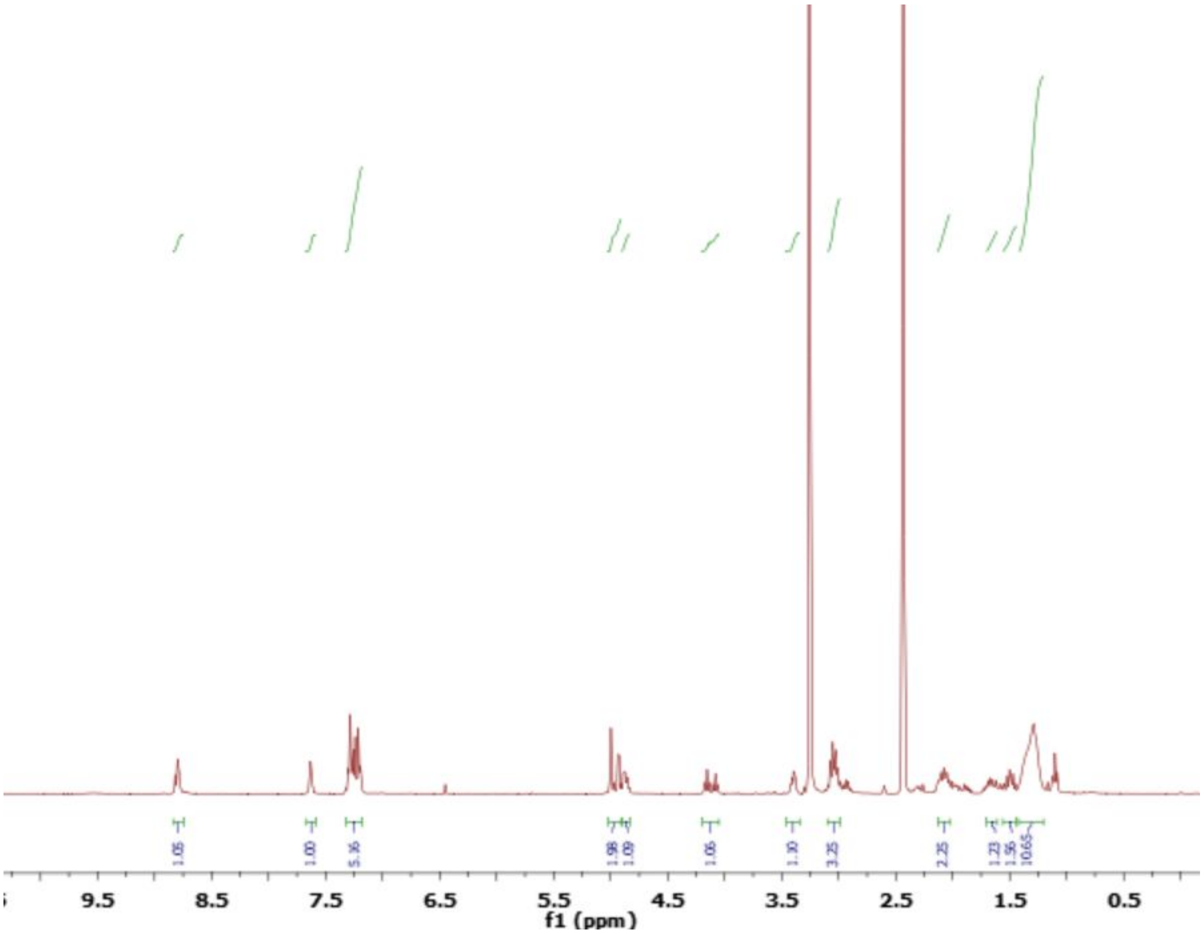
1.05
1.00
5.16
1.98
1.09
1.06
1.10
3.25
2.25
1.23
1.56
10.65
9.5
8.5
7.5
6.5
5.5
4.5
3.5
2.5
1.5
0.5
f1 (ppm)

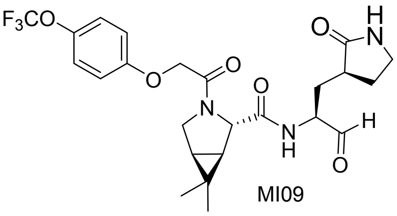
F3CO
O
O
N
O
N
H
O
H
H
N
O

MI09

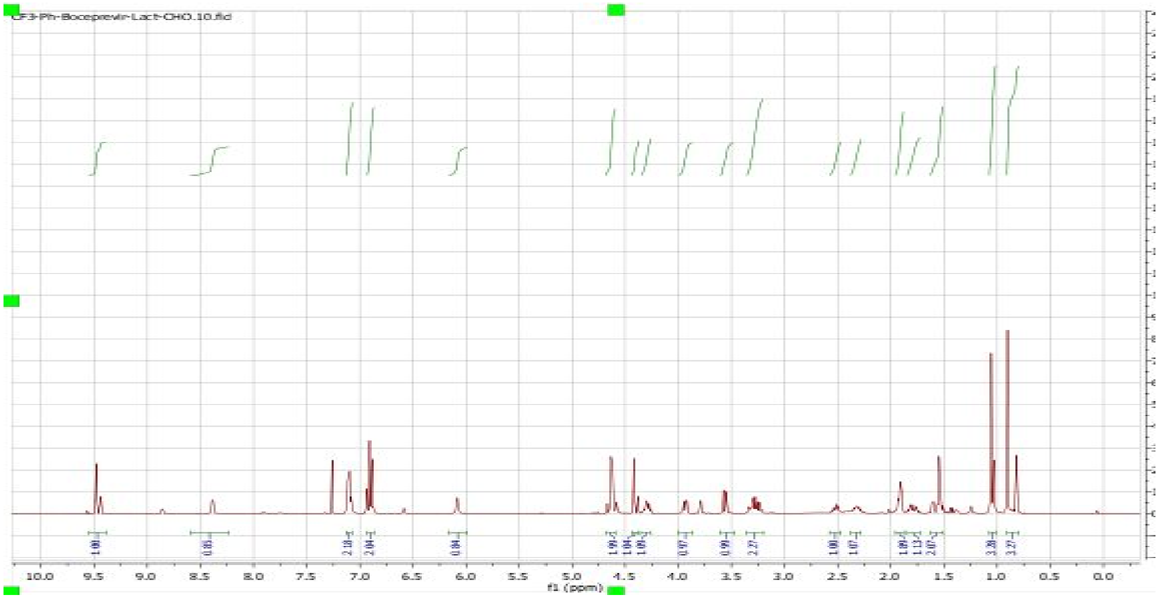
CF3-Ph-Boceprevir-Lact-CHO.10.fid
f1 (ppm)

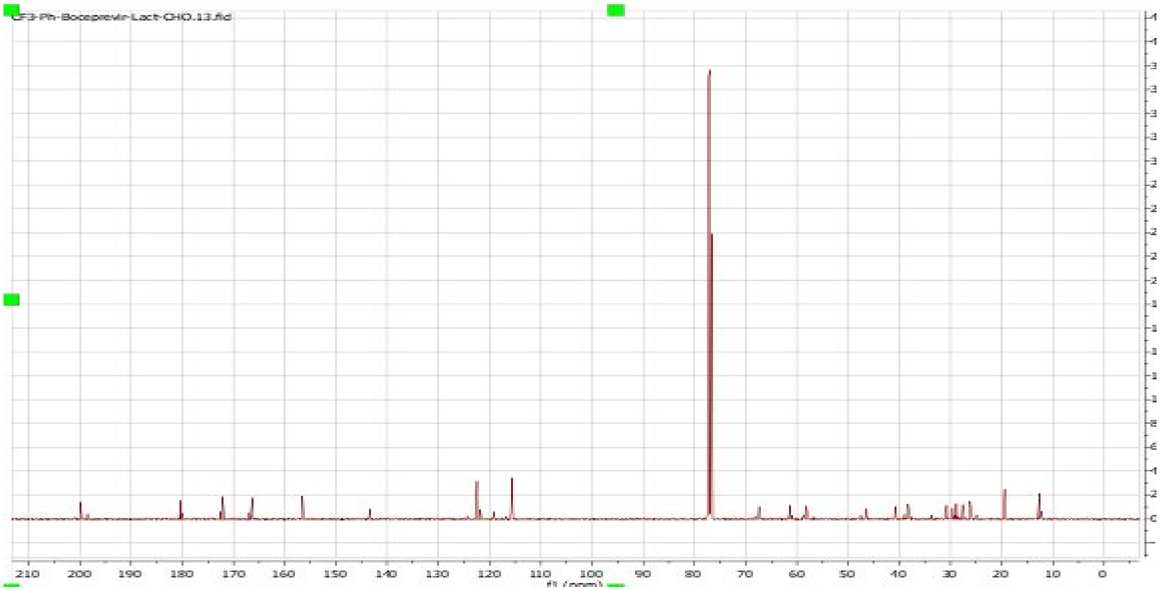
CF3-Ph-Boceprevir-Lact-CHO.13.fid
f1 (ppm)

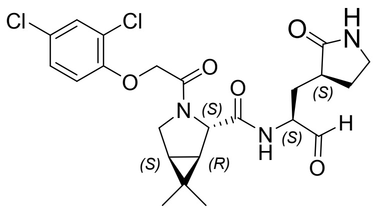

MI14

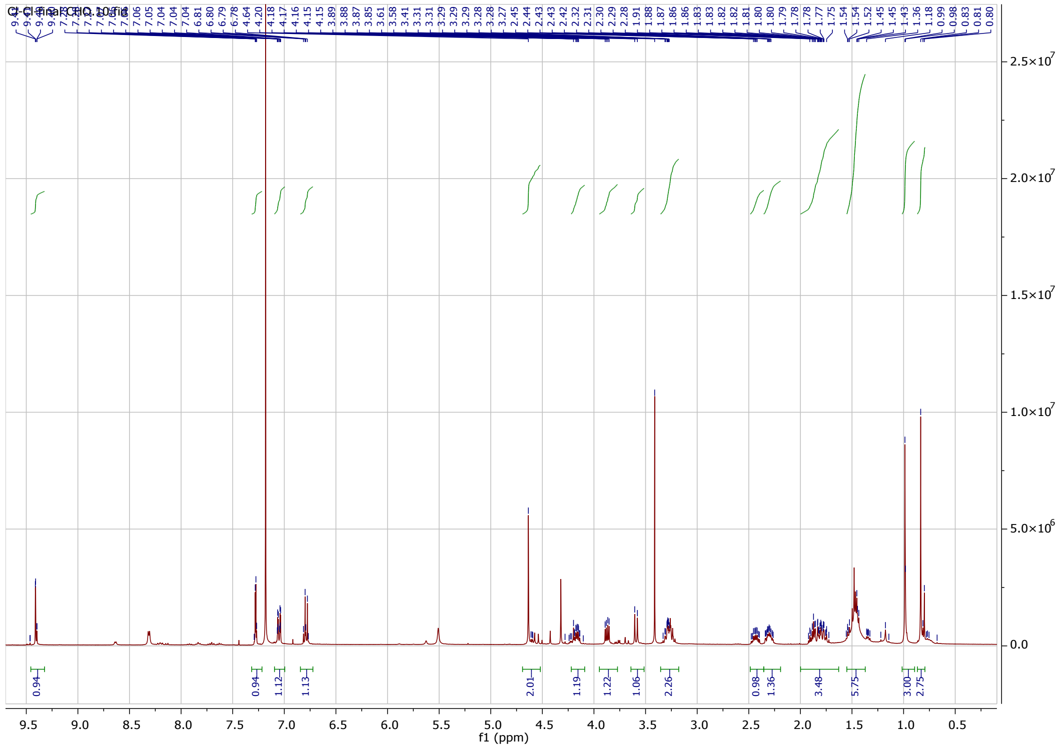

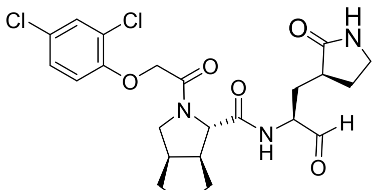

MI30

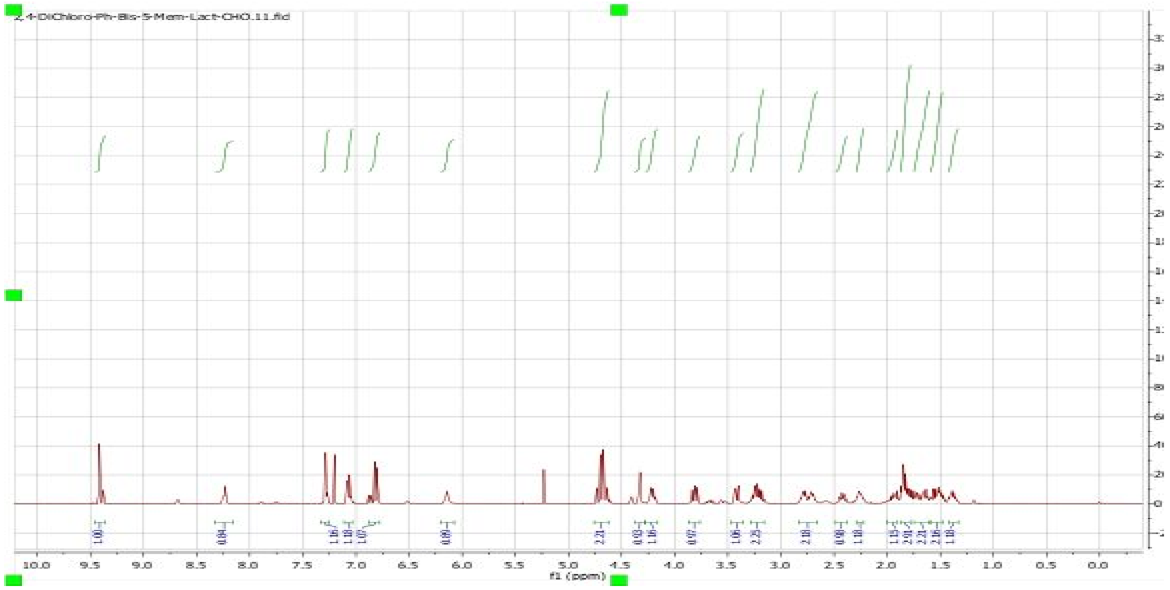
2,4-DiChloro-Ph-Bis-5-Mem-Lact-CHO.11.fid
f1 (ppm)

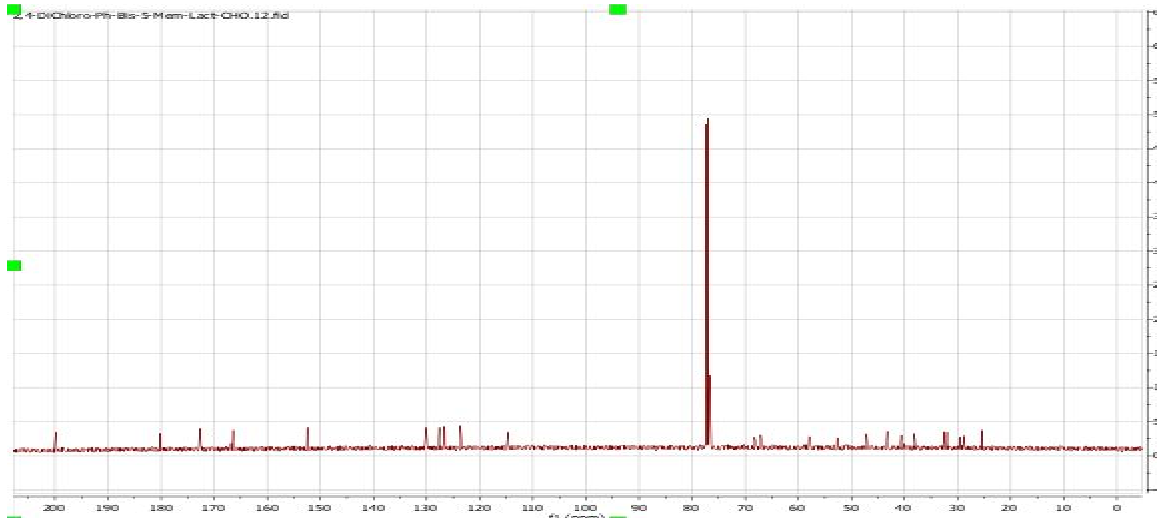
2,4-DiChloro-Ph-Bis-5-Mem-Lact-CHO.12.fid

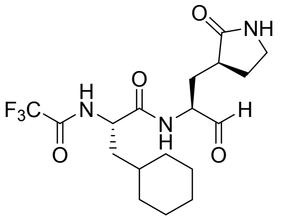

F3C
H
N
O
N
H
O
O
NH
H
O

VB-B-31

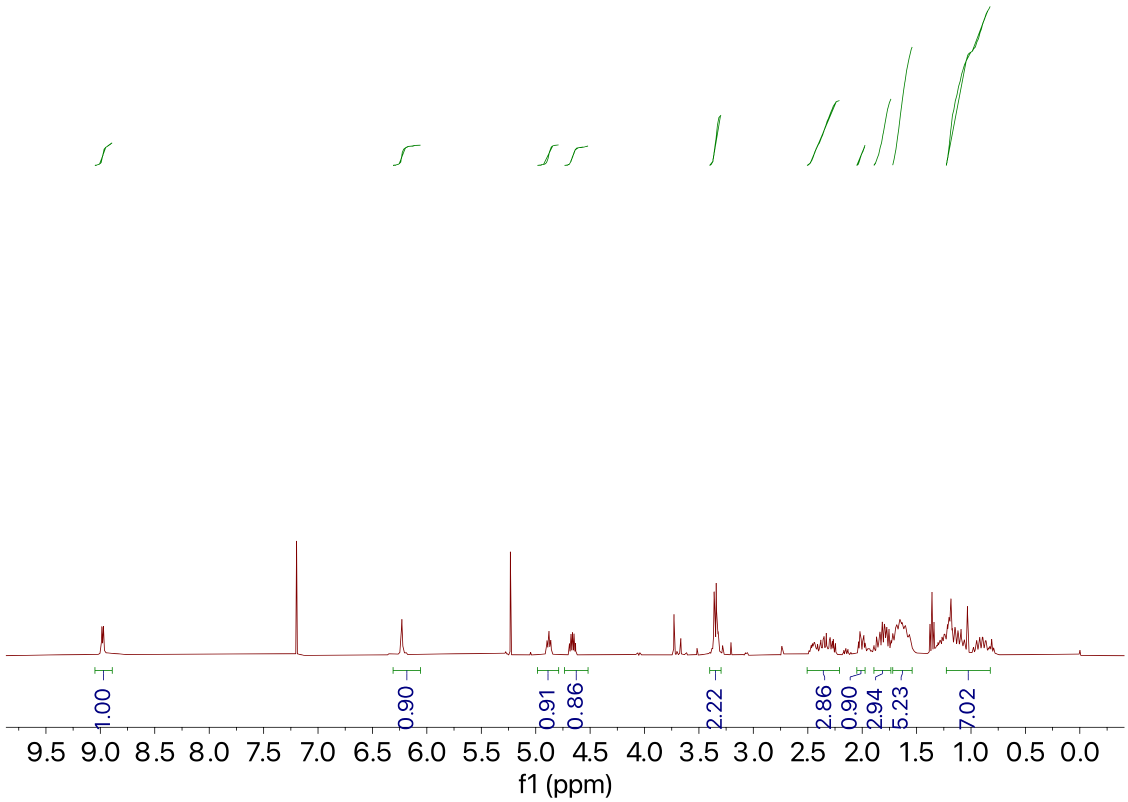

1.00
0.90
0.91
0.86
2.22
2.86
0.90
2.94
5.23
7.02
9.5 9.0 8.5 8.0 7.5 7.0 6.5 6.0 5.5 5.0 4.5 4.0 3.5 3.0 2.5 2.0 1.5 1.0 0.5 0.0
f1 (ppm)

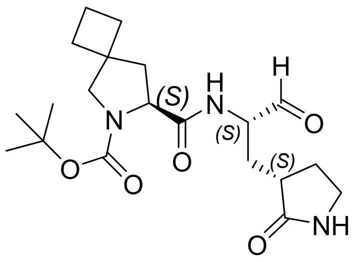
H
(S)
H
N
O
N
(S)
O
O
(S)
O
O
NH

YR-B-101

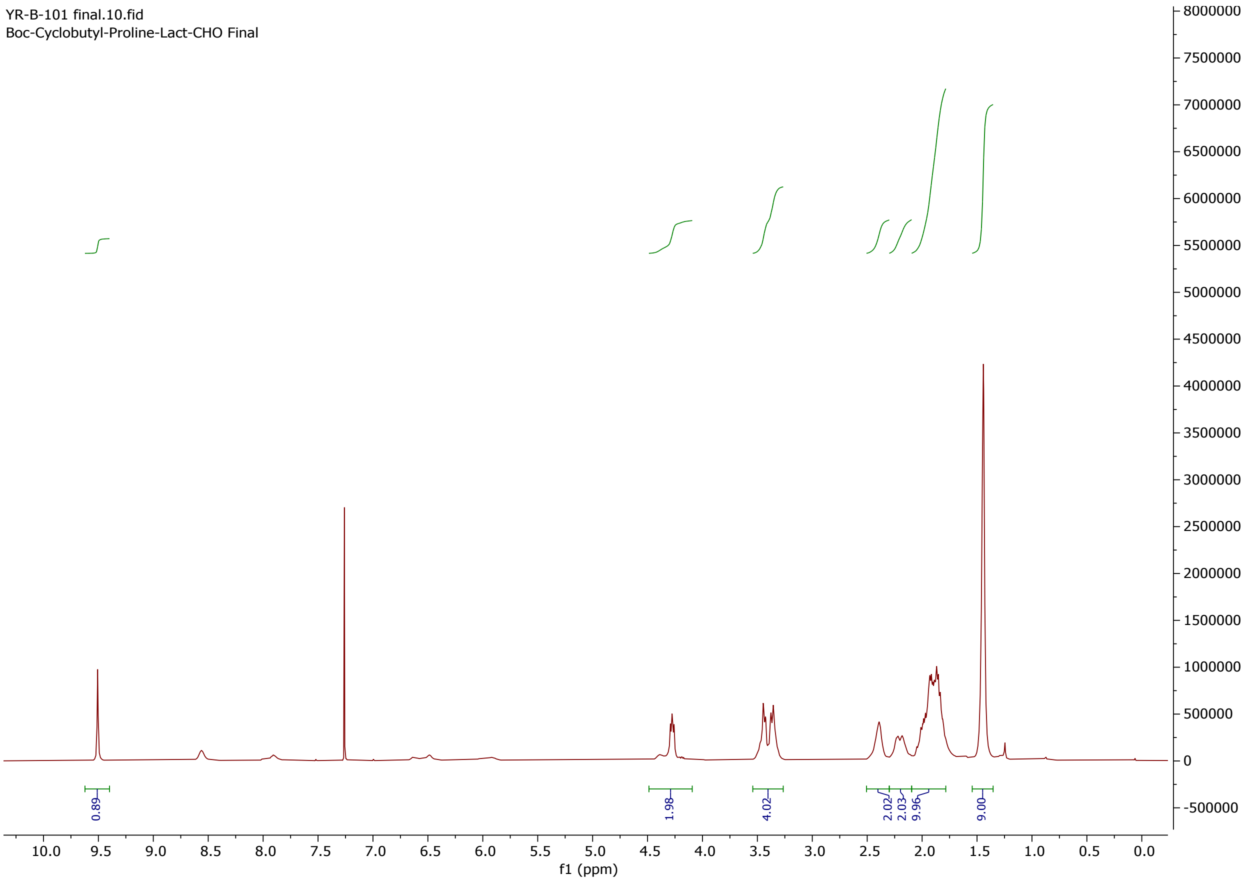
YR-B-101 final.10.fid
Boc-Cyclobutyl-Proline-Lact-CHO Final
0.89
1.98
4.02
2.02
2.03
9.96
9.00
f1 (ppm)

LC-MS

MPI60

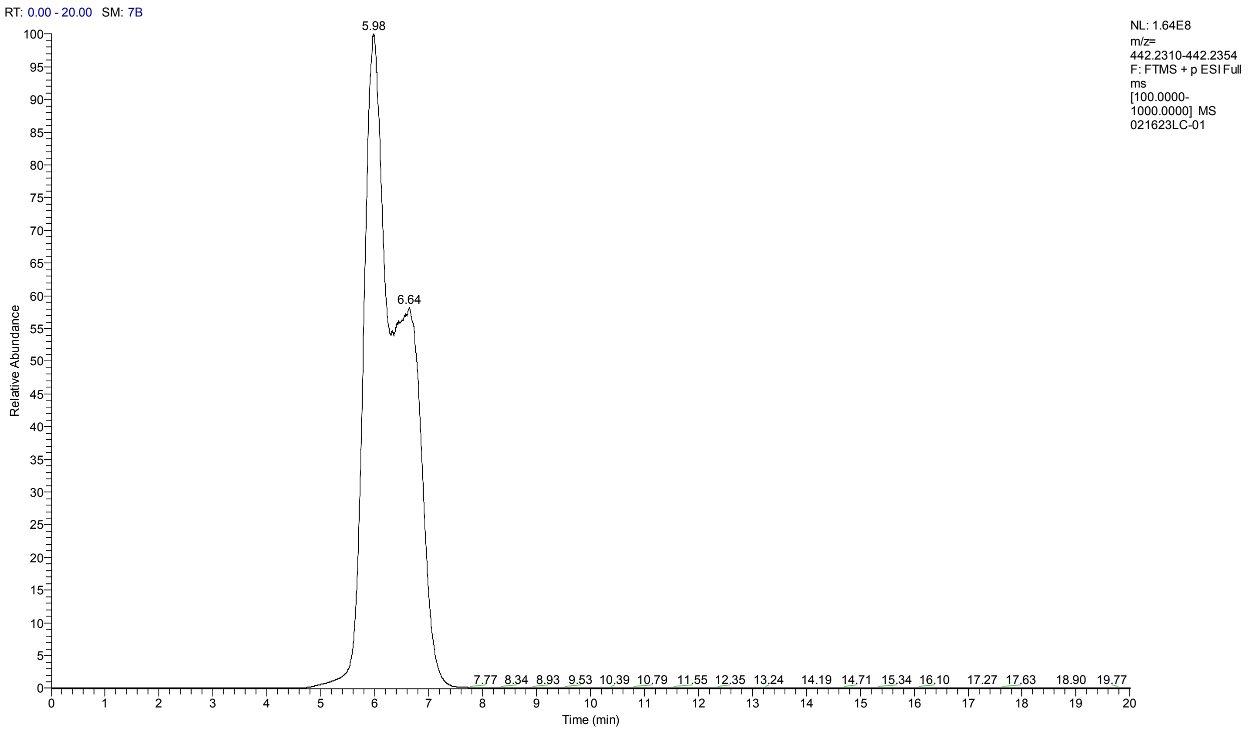

MPI61

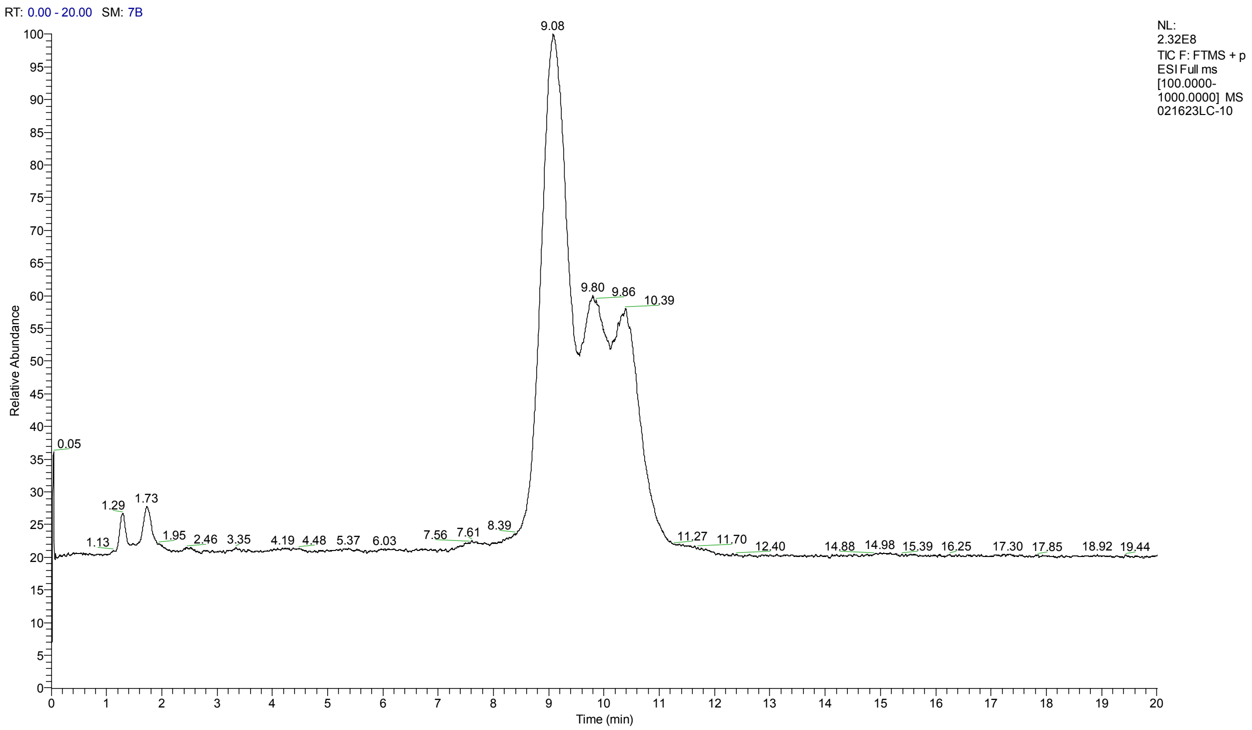

Supplement: Supplementary file 1 — jm3c00221_si_001.pdf [file jm3c00221_si_001.pdf]
